# Self-regulated co-assembly of soft and hard nanoparticles

**DOI:** 10.1038/s41467-021-25995-5

**Published:** 2021-09-28

**Authors:** Yan Cui, Hongyan Zhu, Jiandong Cai, Huibin Qiu

**Affiliations:** 1 School of Chemistry and Chemical Engineering, Shanghai Jiao Tong University, Shanghai, 200240 China; 2 Frontiers Science Center for Transformative Molecules, Shanghai Jiao Tong University, Shanghai, 200240 China; 3 State Key Laboratory of Metal Matrix Composites, Shanghai Jiao Tong University, Shanghai, 200240 China

**Keywords:** Self-assembly, Nanoparticles

## Abstract

Controlled self-assembly of colloidal particles into predetermined organization facilitates the bottom-up manufacture of artificial materials with designated hierarchies and synergistically integrated functionalities. However, it remains a major challenge to assemble individual nanoparticles with minimal building instructions in a programmable fashion due to the lack of directional interactions. Here, we develop a general paradigm for controlled co-assembly of soft block copolymer micelles and simple unvarnished hard nanoparticles through variable noncovalent interactions, including hydrogen bonding and coordination interactions. Upon association, the hairy micelle corona binds with the hard nanoparticles with a specific valence depending exactly on their relative size and feeding ratio. This permits the integration of block copolymer micelles with a diverse array of hard nanoparticles with tunable chemistry into multidimensional colloidal molecules and polymers. Secondary co-assembly of the resulting colloidal molecules further leads to the formation of more complex hierarchical colloidal superstructures. Notably, such colloidal assembly is processible on surface either through initiating the alternating co-assembly from a micelle immobilized on a substrate or directly grafting a colloidal oligomer onto the micellar anchor.

## Abstract (web summary)

Colloidal self-assembly enables bottom-up manufacture of materials with designed hierarchies and functions. Here the authors develop a facile method to construct multidimensional colloidal architectures via the association of soft block copolymer micelles with simple unvarnished hard nanoparticles.

## Introduction

**Fig. 1:**
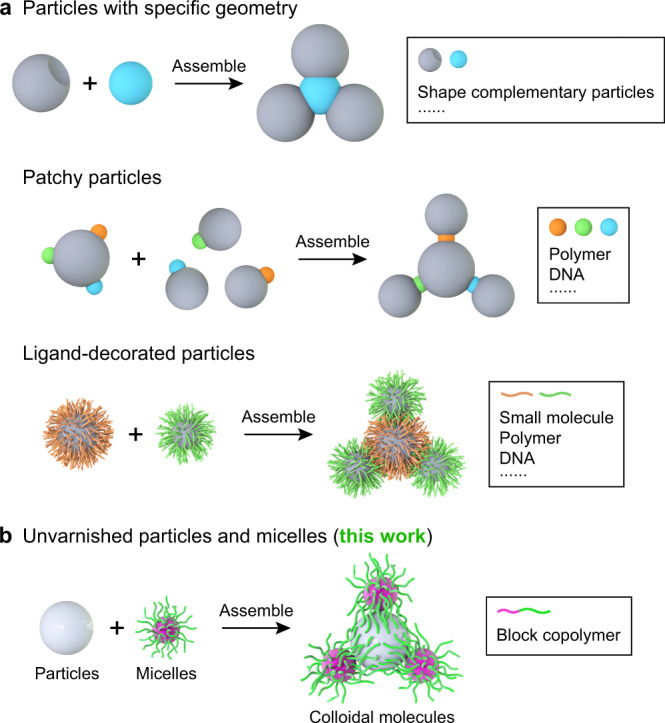
General synthetic strategies for colloidal molecules. **a** Previously reported synthetic strategies for colloidal molecules. **b** Soft-hard co-assembly strategy developed in this work by directly employing unvarnished hard NPs and block copolymer micelles.

Over the past few decades, solution self-assembly of nanoparticles (NPs) has created a series of fascinating colloidal architectures, such as low-dimensional clusters^1–4^, one-dimensional (1D) chains^5–7^, and higher-ordered 2D/3D superstructures^8–11^. Generally, directional modules are planted on the surface of individual NPs to refine the interparticle bonding and hence ensure that the self-organization proceeds in a controllable and predictable manner^12–14^. This normally involves the fabrication of patchy particles with regioselective binding characteristics^15–22^ and the creation of building blocks with specific geometries^23–25^ (Fig. 1a, top and middle panels). However, the enormous difficulties arising from the regioselective surface functionalization and shape-specific synthesis at the nanoscale substantially limit the extension and practical utilization of these guided colloidal self-assembly systems^14,26^. So far, controlled assembly of relatively simple NPs remains a tremendous challenge in spite of a couple of recent advances^27–29^ (Fig. 1a, bottom panel).

**Fig. 2:**
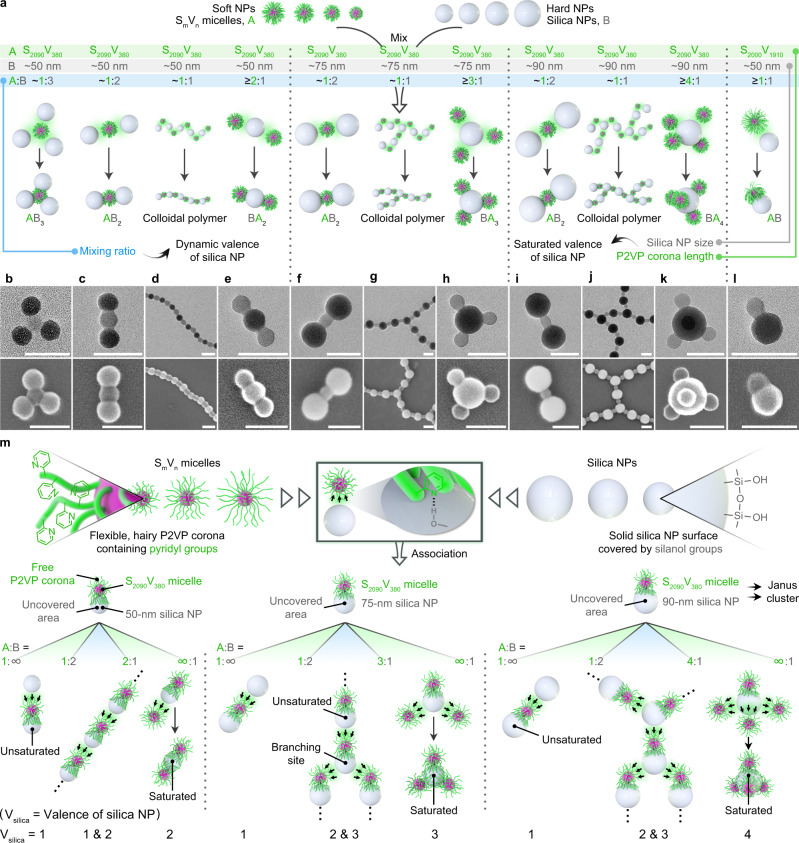
Primary co-assembly of SV micelles and silica NPs in tunable valences. The valence of the co-assembled structure can be precisely regulated by the relative size and feeding ratio of SV micelles and silica NPs. **a** Schematic illustration and **b**–**l** corresponding transmission electron microscopy (TEM) and scanning electron microscopy (SEM) images for typical colloidal architectures formed by diverse combinations of S_m_V_n_ micelles (S_m_V_n_ = PS_m_-*b*-P2VP_n_, denoted as A) and silica NPs (denoted as B) in different A:B feeding ratios. **m** Schematic illustration for the association process of S_2090_V_380_ micelles and 50-nm/75-nm/90-nm silica NPs, which probably involves the formation of an intermediate Janus cluster once a S_2090_V_380_ micelle binds to a silica NP and the subsequent association at the SV micelle pole and/or the silica NP pole. The relative size determines the area a micelle can cover on a silica NP surface, and the feeding ratio regulates the arrangement between micelles and silica NPs. The PS core-forming and the P2VP corona-forming blocks are indicated by pink and green colors, respectively. The valence of silica NP (V_silica_) means the number of surrounding, actually associated soft NPs. Scale bars = 100 nm.

Previous studies have demonstrated that block copolymer micelles with a poly(2-vinylpyridine) (P2VP) corona can adsorb onto the surface of silica particles to generate core-satellite structures via hydrogen bonding (H-bonding) interactions between the pyridyl groups of the P2VP corona and the silanol groups on the silica particles surface^30,31^. Here, we narrow down the size of silica particles (denoted as B, Fig. 2a and Supplementary Fig. 1b) to tens of nanometers and find that their simple association (Supplementary Fig. 3) with soft PS_m_-*b*-P2VP_n_ (PS = polystyrene, the subscripts denote the number-average degree of polymerization, the block copolymer is simply denoted as S_m_V_n_, S = PS, V = P2VP) spherical micelles (with a PS core and a P2VP corona, denoted as A, Fig. 2a and Supplementary Fig. 2) allows the formation of well-organized multidimensional colloidal architectures (Fig. 1b).

## Results and discussion

### Primary co-assembly of SV micelles and silica NPs

We first studied the association between S_2090_V_380_ micelles (ca. 40 nm in diameter, estimated by TEM, mainly reflecting the size of PS cores) and 50-nm (number-average diameter, estimated by TEM) silica NPs in various A:B mixing number ratios. With an increase in the A:B ratio, the assembled structures varied from silica-NPs-capped AB_3_ tetramers (Fig. 2b) and AB_2_ trimers (Fig. 2c) to linear and slightly branched colloidal copolymers (Fig. 2d) and further to micelles-capped BA_2_ trimers (Fig. 2e, see Supplementary Figs. 4 to 10 for more details). Subsequently, we employed relatively larger 75-nm silica NPs and also finely tuned the A:B ratio. Similarly, the resultant morphology evolved from silica-NPs-capped AB_2_ trimers (Fig. 2f) to highly branched colloidal copolymers (Fig. 2g) and relatively extended 2D networks (Supplementary Fig. 14) and further to micelles-capped BA_3_ tetramers (Fig. 2h) with an increase in A:B (see Supplementary Figs. 11–16 for more details). It seemed that larger silica NPs allowed the formation of higher-valence colloidal molecules. Indeed, when 90-nm silica NPs were used, the co-assembly eventually gave rise to micelles-capped BA_4_ pentamers (Fig. 2k) at a high A:B ratio, following the appearance of silica-NPs-capped AB_2_ trimers (Fig. 2i), highly branched colloidal copolymers (Fig. 2j), large-scale 3D stacked monoliths (Supplementary Fig. 20) and other intermediate species (see Supplementary Figs. 17–23 for more details).

Apparently, the valence of the silica NP varies with the initial A:B feeding ratio and ultimately reaches a maximum value depending on its actual size. For a 50-nm silica NP (Fig. 2m, left panel, and Supplementary Fig. 4), one of its hemispheres may be covered by the P2VP corona once it binds with a S_2090_V_380_ micelle. This spontaneously creates an intermediate Janus structure (Supplementary Figs. 24 and 26) which substantially provides the directional interaction for the subsequent co-assembly^28,29^. At a low A:B ratio (1:3~1:2), the free P2VP corona of the SV micelle pole of the intermediate Janus entity can further bind with another one or two silica NPs to generate silica-NPs-capped AB_2_ trimer or AB_3_ tetramer (Supplementary Figs. 5 and 6). The situation dramatically changes once the A:B ratio exceeds 1:2 as the continuous additive co-assembly at both the SV micelle and silica NP poles leads to linear colloidal oligomers and polymers. Similar to a typical alternating condensation polymerization, the degree of such colloidal polymerization varied with the initial mixing ratio of SV micelles and silica NPs (Supplementary Fig. 4), and reached a maximum at A:B = 1:1 (Supplementary Fig. 8). Moreover, the ends of the oligomers were found to be capped with silica NPs at 1:2 < A:B < 1:1 and SV micelles at 1:1 < A:B < 2:1 (Supplementary Fig. 7). Ultimately, at A:B ≥ 2:1, the excess SV micelles terminate the silica NP pole of the Janus intermediate and render the formation of BA_2_ trimers (Supplementary Fig. 9). In this case, the surface of the central silica NP is saturatedly covered by the P2VP coronas and becomes inert to further added SV micelles (Supplementary Fig. 10). Consequently, the maximum valence of the 50-nm silica NP turns to be 2 against the S_2090_V_380_ micelle at the presently studied condition.

For 75-nm and 90-nm silica NPs (Fig. 2m, middle and right panels, and Supplementary Figs. 11 and 17), their binding with S_2090_V_380_ micelles also generates Janus intermediates (Supplementary Figs. 25 and 26) at the initial association stage. However, the saturated valence of the silica NP increases to 3 and 4, respectively, as a result that larger silica NPs offer more accommodating sites for P2VP attachment. Notably, the BA_3_ tetramers and BA_4_ pentamers adopt regular triangular (Supplementary Fig. 16) and tetrahedral (Supplementary Fig. 22) shapes, respectively, which is probably to maximize the H-bonding interactions and to minimize the repulsion between the adjacent P2VP coronas. On the other hand, the enriched binding vacancy of the silica NP and the relatively flexible and reversible interactions between the soft and hard NPs also facilitate the construction of various dynamically-trapped colloidal architectures. For example, at A:B ≈ 1:1, the higher-valence silica NP poles allowed the association with more than one SV micelles, leading to the formation of highly branched colloidal chains (Supplementary Figs. 13 and 19). At a higher A:B ratio (3:2 for 75-nm silica NPs and 2:1 for 90-nm silica NPs), the Janus intermediates and the remaining micelles were linked into relatively extended 2D networks (Supplementary Fig. 14) and large-scale 3D stacked monoliths (Supplementary Fig. 20). Such dynamic valence is predominantly regulated by the A:B feeding ratio and appears to be a consequence of the balance of the adhesive force from the H-bonding interactions and the repulsive and dispersion forces from the inter-corona and corona/solvent interactions. It should be noted that a larger P2VP corona will lower the saturated valence of the silica NP since longer P2VP chains cover a larger surface area of the silica NP (Fig. 2l and Supplementary Figs. 27 and 28).

### Secondary co-assembly of SV micelles and silica NPs

**Fig. 3:**
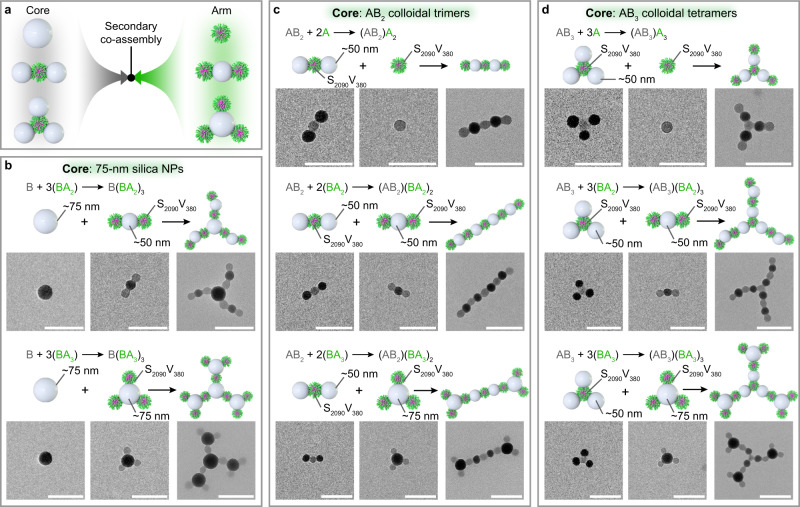
Secondary co-assembly of SV micelles and silica NPs. Hierarchical assembly of primary colloidal molecules favors the construction of core-arm-structured colloidal conjugates. **a** Schematic illustration of secondary co-assembly of core and arm species to generate core-arm-structured colloidal conjugates. **b** Co-assembly of 75-nm silica NPs with S_2090_V_380_-micelles-capped BA_2_ (B = 50-nm silica NP) trimers and BA_3_ (B = 75-nm silica NP) tetramers into branched colloidal conjugates. **c** Co-assembly of silica-NPs-capped AB_2_ (A = S_2090_V_380_ micelle, B = 50-nm silica NP) trimers with S_2090_V_380_ micelles, S_2090_V_380_-micelles-capped BA_2_ (B = 50-nm silica NP) trimers, and S_2090_V_380_-micelles-capped BA_3_ (B = 75-nm silica NP) tetramers into linear colloidal conjugates. **d** Co-assembly of silica-NPs-capped AB_3_ (A = S_2090_V_380_ micelle, B = 50-nm silica NP) tetramers with S_2090_V_380_ micelles, S_2090_V_380_-micelles-capped BA_2_ (B = 50-nm silica NP) trimers, and S_2090_V_380_-micelles-capped BA_3_ (B = 75-nm silica NP) tetramers into branched colloidal conjugates. Scale bars = 200 nm.

Subsequently, we explored the secondary, hierarchical assembly of the above primary colloidal molecules (Fig. 3a). The combination of 75-nm silica NPs with S_2090_V_380_-micelles-capped BA_2_ (B = 50-nm silica NP) trimers and BA_3_ (B = 75-nm silica NP) tetramers generated core-arm-structured branched B(BA_2_)_3_ and B(BA_3_)_3_ colloidal conjugates, respectively (Fig. 3b and Supplementary Figs. 29 and 30). The association of CO_2_-like AB_2_ (A = S_2090_V_380_ micelle, B = 50-nm silica NP) trimers with S_2090_V_380_ micelles, S_2090_V_380_-micelles-capped BA_2_ (B = 50-nm silica NP) trimers, and S_2090_V_380_-micelles-capped BA_3_ (B = 75-nm silica NP) tetramers allowed the formation of linear (AB_2_)A_2_, (AB_2_)(BA_2_)_2_, and (AB_2_)(BA_3_)_2_ colloidal conjugates (Fig. 3c and Supplementary Figs. 31 to 33). We also used BH_3_-like AB_3_ (A = S_2090_V_380_ micelle, B = 50-nm silica NP) colloidal tetramers to assemble with S_2090_V_380_ micelles, S_2090_V_380_-micelles-capped BA_2_ (B = 50-nm silica NP) trimers, and S_2090_V_380_-micelles-capped BA_3_ (B = 75-nm silica NP) tetramers. The triangular AB_3_ core provided a branching junction for hierarchical construction of more complex (AB_3_)A_3_, (AB_3_)(BA_2_)_3_, and (AB_3_)(BA_3_)_3_ colloidal conjugates (Fig. 3d and Supplementary Figs. 34–36).

### Tunable co-assembly of SV micelles and functional hard NPs

**Fig. 4:**
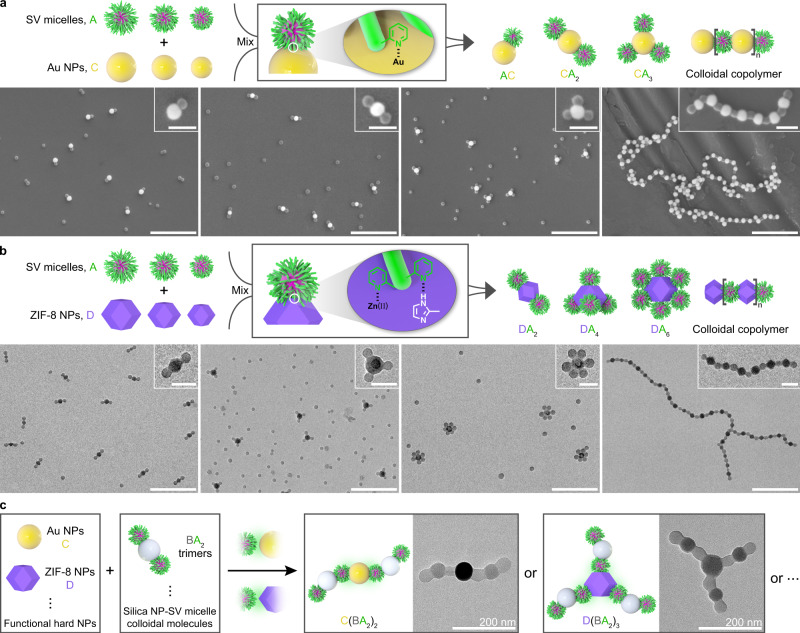
Tunable co-assembly of SV micelles and various functional hard NPs. A diverse array of hard NPs can be assembled with SV micelles in a controlled manner via noncovalent interactions, such as H-bonding and/or coordination interactions. **a** Colloidal molecules and copolymers assembled from 47-nm Au NPs and SV micelles. SEM images show resultant structures formed by the association of 47-nm Au NPs with (from left to right) S_2040_V_1590_, S_1520_V_370_, S_460_V_190_, and S_1220_V_440_ micelles, respectively. Scale bars = 500 nm; inset scale bars = 100 nm. **b** Colloidal molecules and copolymers generated from ZIF-8 NPs and SV micelles. TEM images display resultant architectures formed by (from left to right) 53-nm ZIF-8 NPs and S_2090_V_380_ micelles, 70-nm ZIF-8 NPs and S_1220_V_440_ micelles, 70-nm ZIF-8 NPs and S_1030_V_150_ micelles, 53-nm ZIF-8 NPs and S_2090_V_380_ micelles, respectively. Scale bars = 500 nm; inset scale bars = 100 nm. **c** Core-arm-structured multicompartment colloidal conjugates assembled from functional hard NPs (Au NPs and ZIF-8 NPs) and colloidal molecules derived from silica NPs and SV micelles. TEM images present C(BA_2_)_2_ and D(BA_2_)_3_ colloidal conjugates formed by S_2090_V_380_-micelles-capped BA_2_ (B = 50-nm silica NP) trimers with 57-nm Au NPs and 70-nm ZIF-8 NPs, respectively.

The hairy P2VP corona also favored the association of SV micelles with a variety of other hard NPs. The association between S_2040_V_1590_, S_1520_V_370_, S_460_V_190_, S_1220_V_440_ micelles and 47-nm gold (Au) NPs (denoted as C, Supplementary Fig. 1c) through coordination between the P2VP pyridyl groups and the Au NP surface generated well-defined AC dimers, CA_2_ trimers, CA_3_ tetramers, and colloidal copolymers (Fig. 4a and Supplementary Figs. 37–40). Such metal species can also be encapsulated in silica NPs (core/shell structured silica-coated Au NPs, Supplementary Fig. 1d) before associating with SV micelles, which led to the formation of micelles-capped colloidal trimers and alternating colloidal copolymers (Supplementary Fig. 41a). Beside to inorganic NPs, polymeric NPs such as polydopamine (PDA) NPs were also utilized to fabricate micelles-surrounded core-satellite nanostructures (Supplementary Fig. 41b). We further prepared metal-organic framework NPs such as zeolitic imidazolate framework-8 (ZIF-8) NPs (denoted as D, Supplementary Fig. 1e) and they also interacted with the P2VP corona presumably due to the H-bonding between the 2-methylimidazolate linker in ZIF-8 and the pyridyl groups and the coordination of Zn(II) to the pyridyl groups. Consequently, DA_2_ trimers, DA_4_ pentamers, DA_6_ heptamers, and colloidal copolymers were constructed by mixing 53-nm and 70-nm ZIF-8 NPs with S_2090_V_380_, S_1220_V_440_, and S_1030_V_150_ micelles, respectively (Fig. 4b and Supplementary Figs. 42–46). Subsequently, the colloidal molecules derived from silica NPs and SV micelles were conjugated together with Au NPs and ZIF-8 NPs to fabricate multicompartment colloidal superstructures. For example, core-arm-structured C(BA_2_)_2_ and D(BA_2_)_3_ colloidal conjugates were constructed by mixing S_2090_V_380_-micelles-capped BA_2_ (B = 50-nm silica NP) trimers with 57-nm Au NPs and 70-nm ZIF-8 NPs, respectively (Fig. 4c and Supplementary Figs. 47 and 48).

### Co-assembly of SV micelles and silica NPs on a surface

**Fig. 5:**
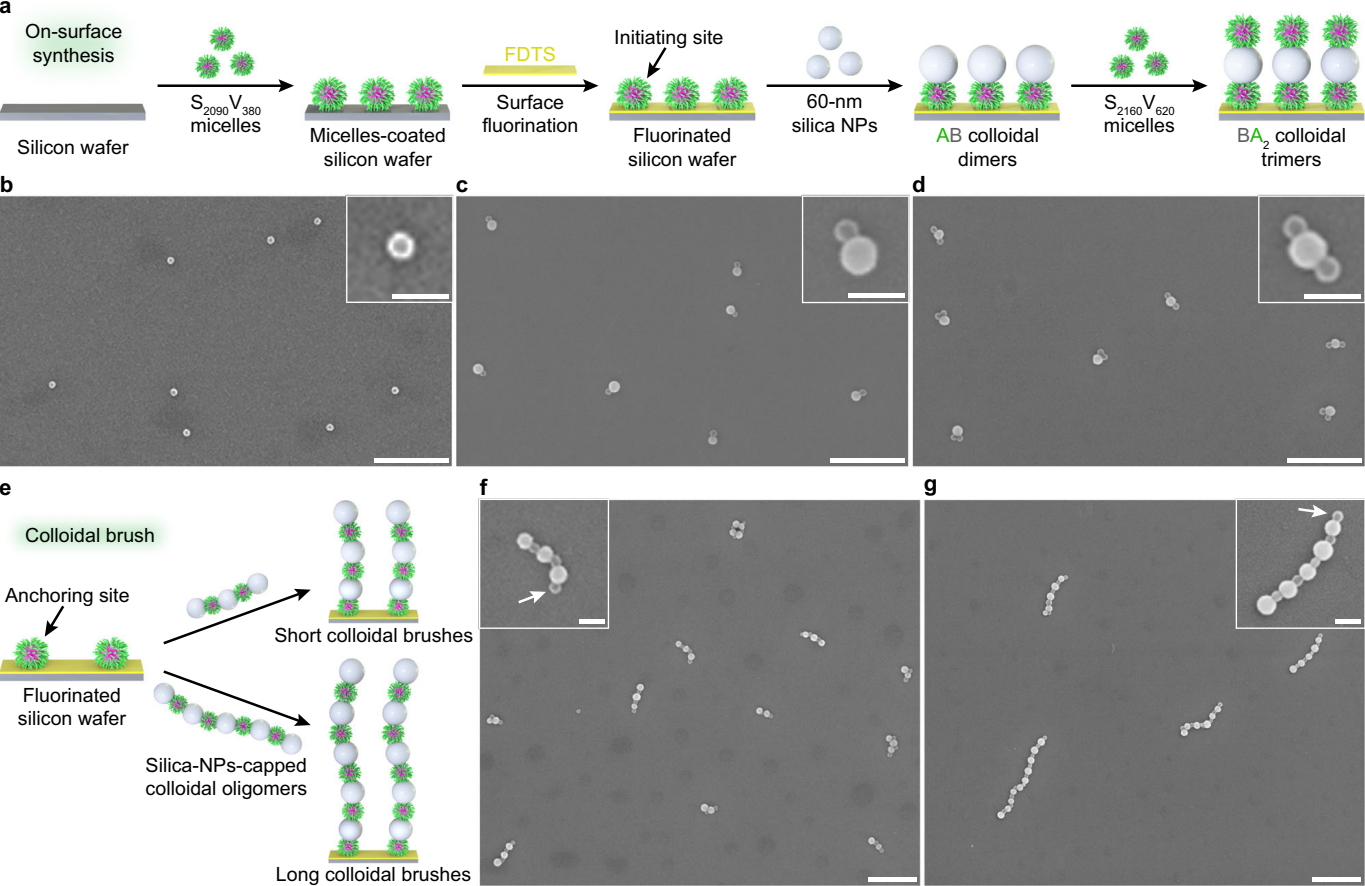
Programmable co-assembly of SV micelles and silica NPs on surface. **a** On-surface synthesis of AB dimers and BA_2_ trimers via step-wise, alternative addition of 60-nm silica NPs and S_2160_V_620_ micelles onto S_2090_V_380_ micelles preformatively immobilized on a silicon wafer. The S_2090_V_380_-micelles-coated silicon wafer was subjected to surface fluorination treatment via exposure to a FDTS vapor in a closed environment to block the association of SV micelles with the bare silicon wafer surface in the subsequent co-assembly process. **b**–**d** SEM images of silicon wafers decorated with S_2090_V_380_ micelles (**b**), AB dimers (**c**), and BA_2_ trimers (**d**), respectively. **e** Preparation of colloidal brushes via a “grafting-to” method simply accomplished by dipping the S_2090_V_380_-micelles-coated, fluorinated silicon wafer into an ethanol solution of silica-NPs-capped colloidal oligomers formed by S_2090_V_380_ micelles and 67-nm silica NPs. **f**–**g** SEM images of silicon wafers decorated with short (**f**) and long (**g**) colloidal oligomers, respectively. The white arrows denote the micellar anchors preformatively immobilized on the silicon wafer surface. Scale bars = 500 nm; inset scale bars = 100 nm.

Solid phase synthesis^32^ has been widely used for automated batch manufacturing of sequence-controlled polymers. Inspired by this technique, a silicon wafer was selected as a solid support on which to firmly immobilize the S_2090_V_380_ micelles^33^ to initiate the on-surface growth of colloidal molecules (Fig. 5a). Subsequently, the S_2090_V_380_-micelles-coated silicon wafer was treated with a heptadecafluoro-1,1,2,2-tetrahydrodecyltrichlorosilane (FDTS) vapor to eliminate the free silanol groups on the vacant silicon wafer surface and thus to block the further adsorption of SV micelles in following procedures (Fig. 5b and Supplementary Fig. 49). The S_2090_V_380_-micelles-coated, fluorinated silicon wafer was then directly immersed in an ethanol solution of 60-nm silica NPs. It was found that the silica NPs selectively bound to the preformatively immobilized S_2090_V_380_ micelles, generating AB dimers (Fig. 5c and Supplementary Fig. 50). Further immersion in an ethanol solution of S_2160_V_620_ micelles resulted in the attachment of S_2160_V_620_ micelles onto the silica NP pole of the AB dimers, yielding BA_2_ trimers (Fig. 5d and Supplementary Fig. 50). Although the subsequent fabrication of more advanced oligomers is so far limited due to the distortion of the BA_2_ trimers (majorly in a “v” shape), this on-surface sequential colloidal co-assembly shines lights on the precise synthesis of colloidal molecules and polymers with a desirable composition and sequence. In addition, we also used a “grafting-to” method to decorate the silicon wafer surface with colloidal brushes (Fig. 5e). When the S_2090_V_380_-micelles-coated, fluorinated silicon wafer was immersed in an ethanol solution of silica-NPs-capped colloidal oligomers (formed by S_2090_V_380_ micelles and 67-nm silica NPs, Supplementary Fig. 51), the colloidal chains were grafted onto the silicon wafer surface through the association of the silica NP end with a S_2090_V_380_ micellar anchor (Fig. 5f, g and Supplementary Fig. 52). The length of the colloidal brushes was roughly tuned via the selection of colloidal oligomers with variable degree of polymerization.

The soft-hard co-assembly strategy developed in this work provides a versatile and tunable platform for controlled construction of colloidal molecules and polymers in solution and on surface. The unique self-regulated association behavior simplifies the sculpting of building blocks and enriches the topologies of assembled architectures. We envision this facile and distinctive assembly approach would facilitate the controlled bonding of arbitrary soft and hard NPs given the existence of effective complementary interparticle interactions, thereby enabling the creation of colloidal superstructures with high-level complexities and on-demand functionalities.

## Methods

### Co-assembly of soft NPs and hard NPs in solution

The soft and hard NPs were prepared based on literature reports with slight modifications (more details available in Supplementary Information). A general protocol for the soft-hard co-assembly was as follows. Varying amounts of soft NPs (S_m_V_n_ micelles) in ethanol and hard NPs (such as silica NPs, Au NPs, silica-coated Au NPs, PDA NPs, and ZIF-8 NPs) in ethanol or deionized water were mixed under vigorous stirring to ensure uniformity. After aging for a period of time, the resultant solution was taken out for TEM, SEM, and AFM characterizations. Taking the formation of micelles-capped BA_2_ trimers for example: an ethanol solution of 50-nm silica NPs were instantly injected into an ethanol solution of S_2090_V_380_ micelles under vigorous stirring; subsequently, the mixture was kept under mild stirring at room temperature for 12 h. It should be noted that for the fabrication of relatively extended 2D networks, large-scale 3D stacked monoliths, and micelles-capped nanoclusters the co-assembly was performed at 80 °C in a sealed vial in order to weaken the H-bonding and retard the rapid association of soft and hard NPs^34,35^.

### Preparation of micelles-coated, fluorinated silicon wafers

A silicon wafer (0.5 cm × 0.5 cm) was sonicated in acetone and water for 30 min, respectively, and dried under a gentle stream of nitrogen. Then, the silicon wafer was subjected to O_2_-plasma treatment for 10 min to enrich the surface silanol groups. Subsequently, 10 μL of a 20-fold diluted ethanol solution of S_2090_V_380_ micelles was spin-coated onto the silicon wafer using a spin coater (6000 rpm, 1 min). The resulting silicon wafer was allowed to age at room temperature for 12 h. Subsequent fluorination treatment was conducted as previously reported with slight modifications^36^. Typically, the micelles-coated silicon wafer was placed in a Petri dish and then the Petri dish was placed in a desiccator, together with another smaller Petri dish containing 100 μL of heptadecafluoro-1,1,2,2-tetrahydrodecyltrichlorosilane (FDTS). The desiccator was connected to the vacuum line of the fume hood to evacuate the air. After 2 h, the fluorinated silicon wafer was taken out and rinsed with ethanol. The surface of the resultant silicon wafer transformed from hydrophilic to hydrophobic, as revealed by the static water CA values (increased from 45° to 112°, Supplementary Fig. 49).

### On-surface synthesis of AB dimers and BA_2_ trimers

The micelles-coated, fluorinated silicon wafer was immersed into an ethanol solution of 60-nm silica NPs. After 2 h, the silicon wafer was taken out and rinsed with ethanol several times. Subsequently, the silicon wafer was immersed into an ethanol solution of S_2160_V_620_ micelles. After 2 h, the silicon wafer was taken out and rinsed with ethanol several times, and dried under a gentle stream of nitrogen.

### Preparation of colloidal brushes through a “grafting-to” approach

The micelles-coated, fluorinated silicon wafer was immersed into an ethanol solution of silica-NPs-capped colloidal oligomers (formed by 67-nm silica NPs and S_2090_V_380_ micelles, with length roughly controlled by tuning the mixing number ratio of S_2090_V_380_ micelles and 67-nm silica NPs, Supplementary Fig. 51) for 15 h. The resultant silicon wafer was taken out and rinsed with ethanol several times, and dried under a gentle stream of nitrogen.

## Supplementary information

Supplementary Information

## Data Availability

The authors declare that all the data supporting the findings of this study are available within the paper and Supplementary Information files, and are available from the corresponding author upon reasonable request.
