# Supplementary material for: Self-regulated co-assembly of soft and hard nanoparticles
Source: Nat Commun. 2021 Sep 28;12:5682. doi: 10.1038/s41467-021-25995-5 (PMC8479080; doi:10.1038/s41467-021-25995-5)
Supplement: Supplementary file 1 — Supplementary Information [file 41467_2021_25995_MOESM1_ESM.pdf]

Supplementary Information

Self-regulated co-assembly of soft and hard nanoparticles

Yan Cui[1], Hongyan Zhu[1], Jiandong Cai[1], Huibin Qiu[1,2,3,*]

[1]School of Chemistry and Chemical Engineering, [2]Frontiers Science Center for Transformative Molecules, [3]State Key Laboratory of Metal Matrix Composites, Shanghai Jiao Tong University, Shanghai 200240, China. These authors contributed equally: Yan Cui, Hongyan Zhu. *email: hbqiu@sjtu.edu.cn

This PDF file includes:

Supplementary Figures 1 to 52

Supplementary Methods

Supplementary References 1 to 6

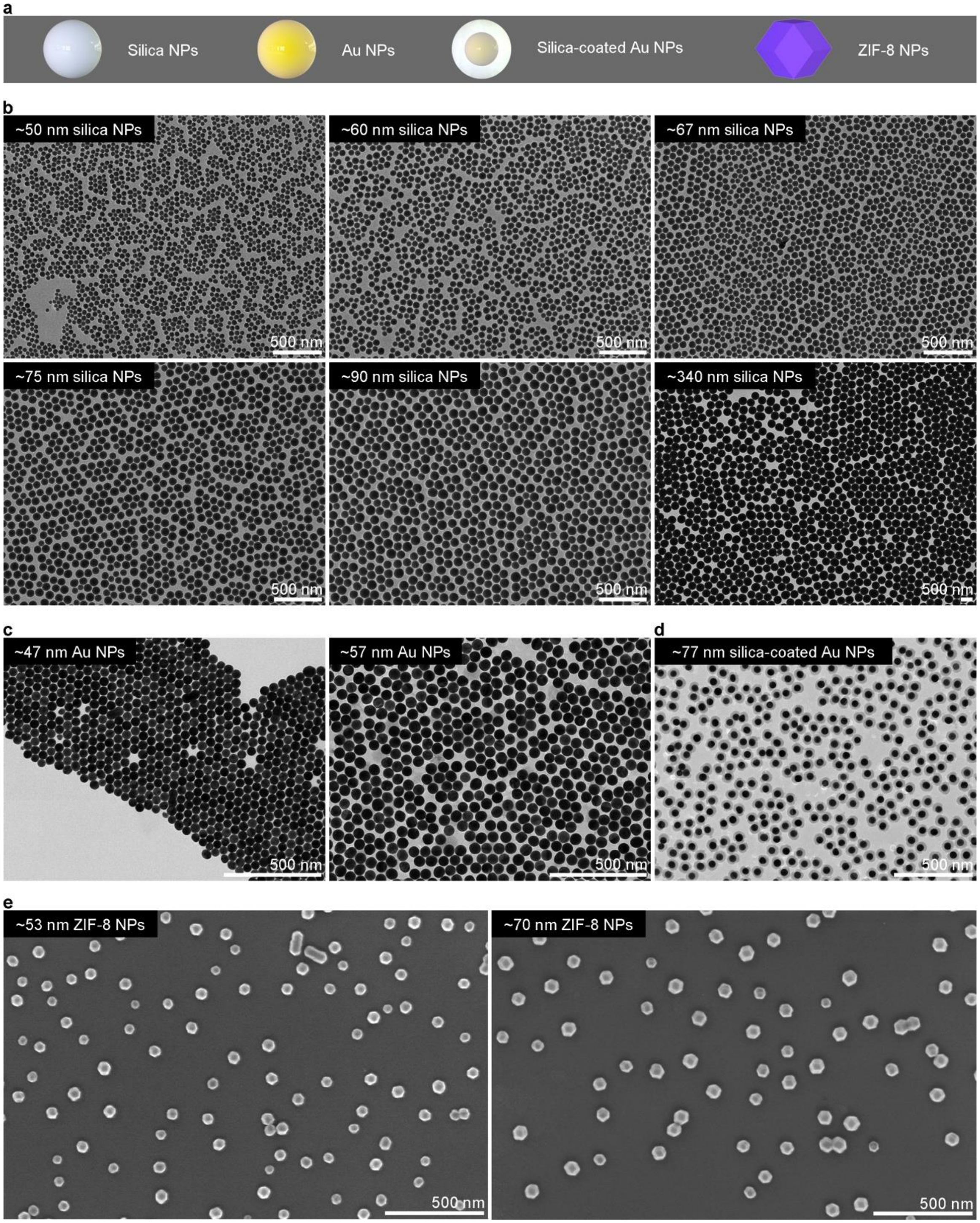

Supplementary Figure 1. Illustration and morphology of hard NPs. (**a**) Schematic illustration of various hard NPs. (**b**) TEM images of silica NPs with number-average diameters of ~50 nm, ~60 nm, ~67 nm, ~75 nm, ~90 nm, and ~340 nm. (**c**) TEM images of Au NPs with number-average diameters of ~47 nm and ~57 nm. (**d**) TEM image of core/shell structured silica-coated Au NPs with overall number-average diameter of ~77 nm. (**e**) SEM images of ZIF-8 NPs with number-average diameters of ~53 nm and ~70 nm.

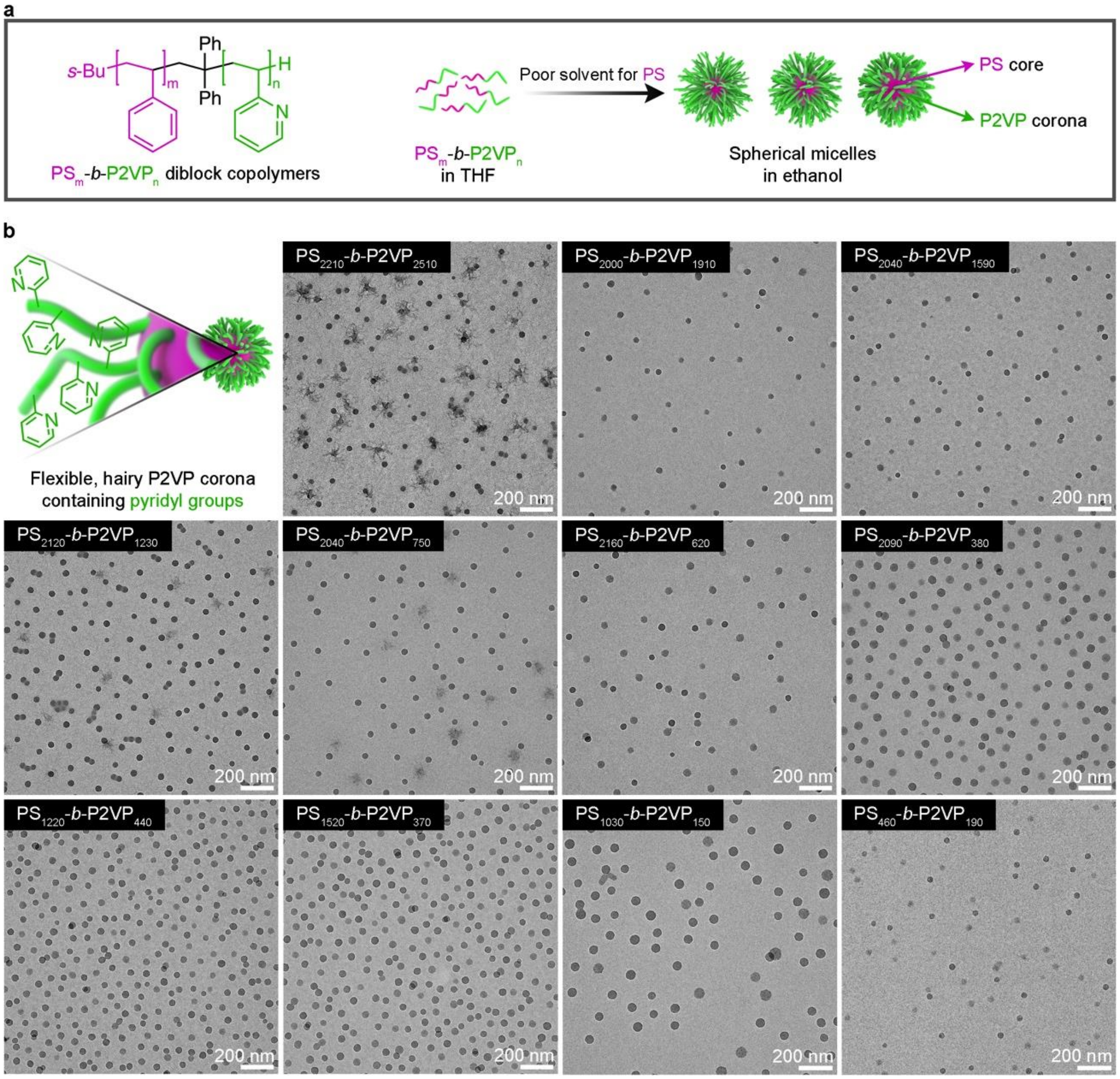

Supplementary Figure 2. Preparation and characterization of SV micelles. (**a**) Molecular structure of PS_m-*b*-$P2VP_n$ (S_mV_n) diblock copolymers and schematic illustration of the preparation process of S_mV_n spherical micelles, which involves the slow introduction of methanol into a THF solution of S_mV_n followed by redispersion in ethanol. The PS core-forming and the P2VP corona-forming blocks are indicated by pink and green colors, respectively. (**b**) Schematic illustration and TEM images of the resultant spherical micelles of various SV diblock copolymers.

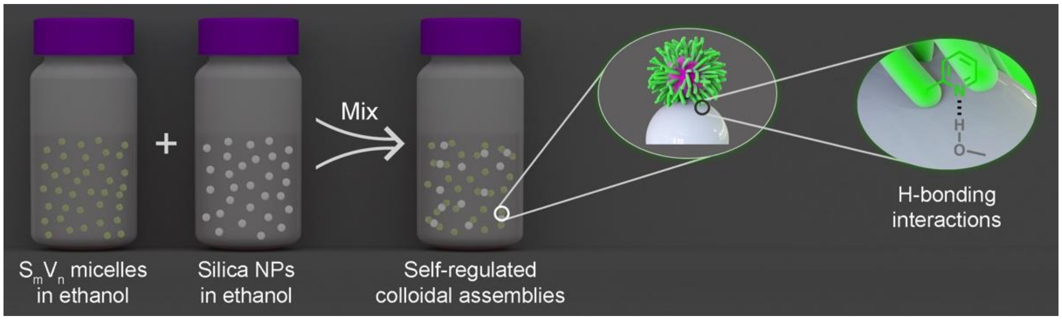

Supplementary Figure 3. Schematic illustration of a typical procedure for the co-assembly of soft and hard NPs. Soft NPs (SV micelles) and hard NPs (such as silica NPs), serving as the building blocks, were directly employed without further pre-describing treatment. The fabrication of colloidal molecules and polymers was achieved by simply mixing the soft and hard NPs together in alcoholic solutions.

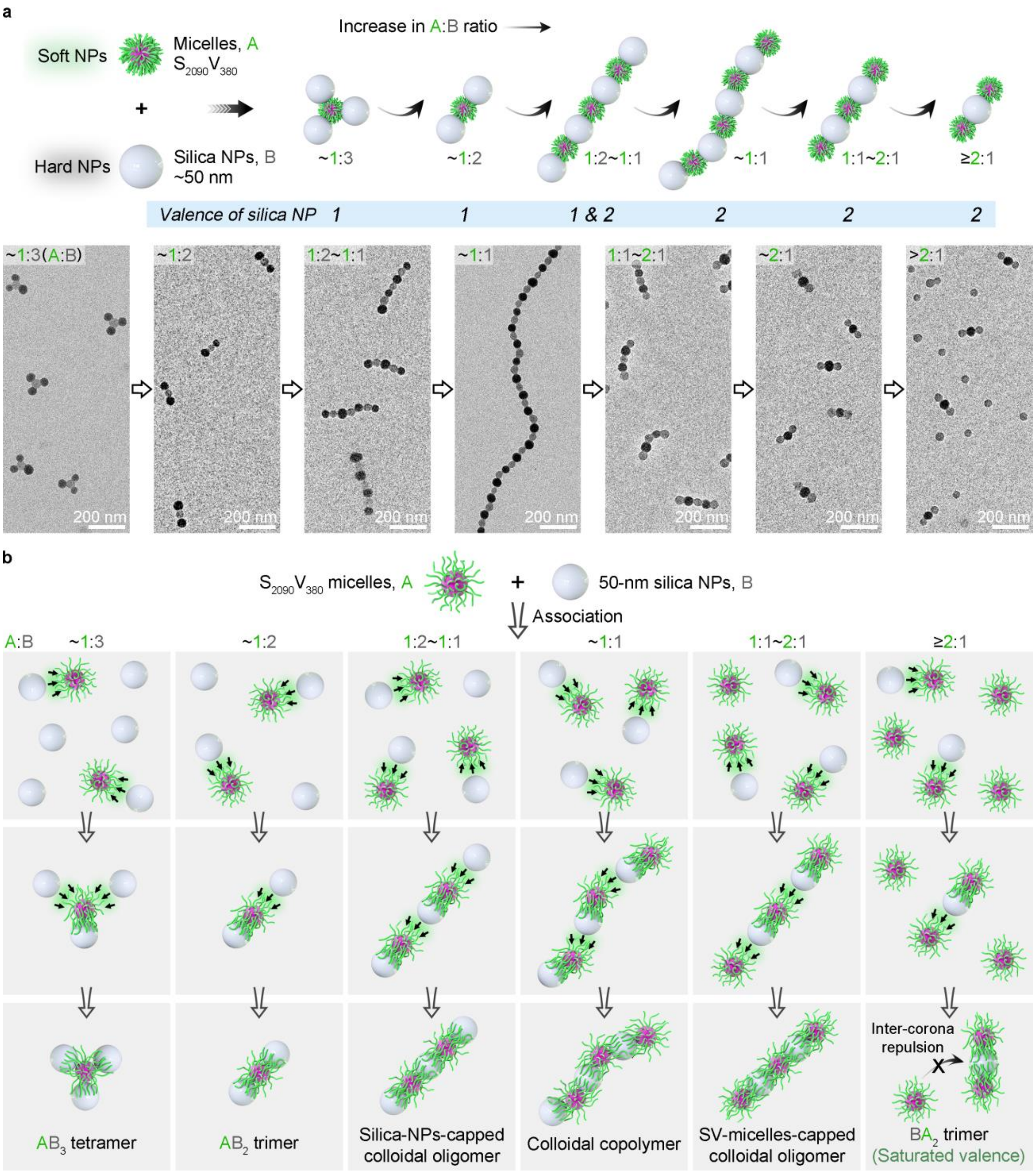

Supplementary Figure 4. Association of $S_{2090}V_{380}$ micelles and 50-nm silica NPs in various A:B feeding ratios. (**a**) General trend of the co-assembly of $S_{2090}V_{380}$ micelles and 50-nm silica NPs in various A:B ratios. The valence of silica NP means the number of surrounding, actually associated soft NPs. TEM images show the assembled colloidal architectures in different A:B ratios. (**b**) Schematic illustration for the possible association process of $S_{2090}V_{380}$ micelles and 50-nm silica NPs in various A:B ratios.

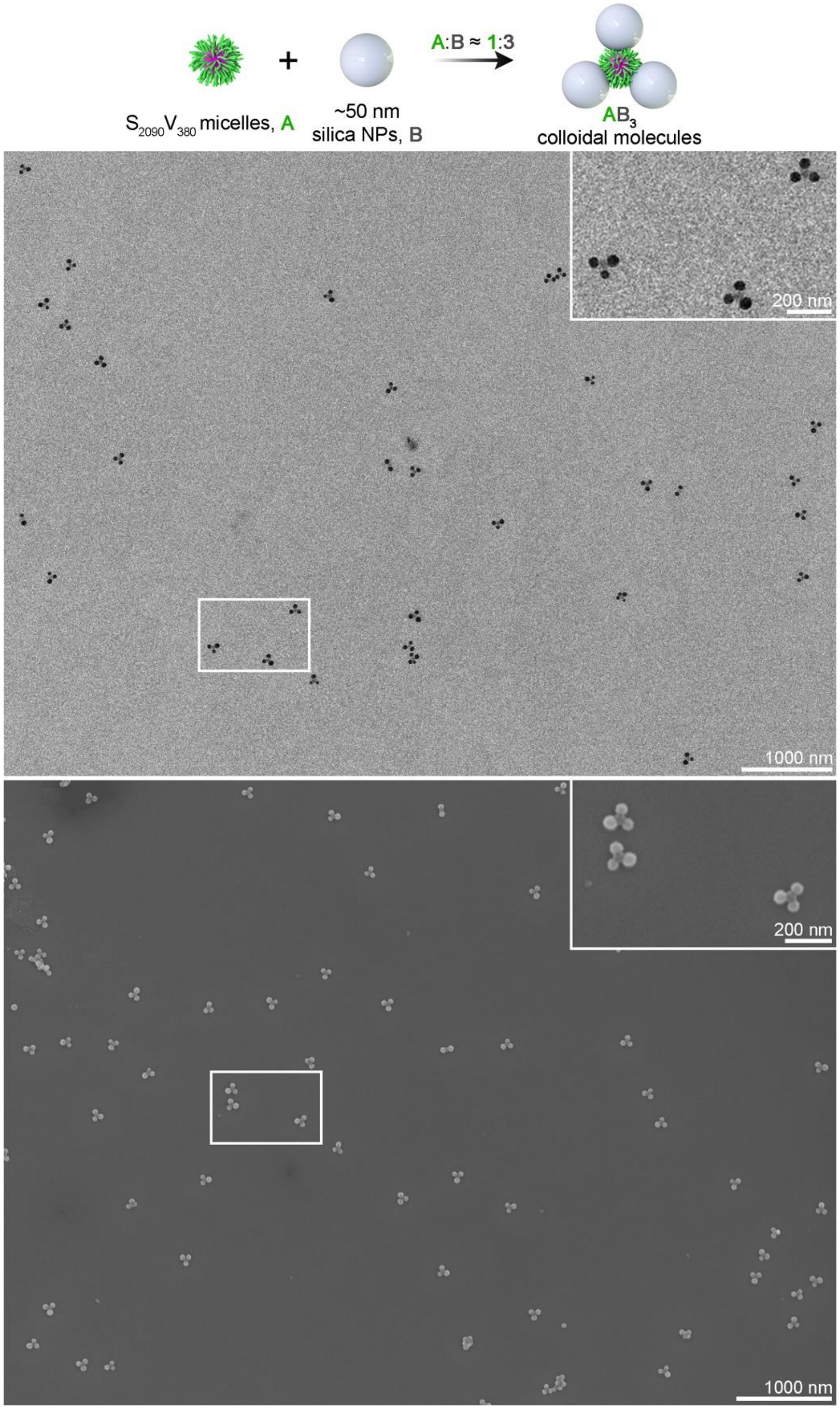

Supplementary Figure 5. Wide-field TEM and SEM images of silica-NPs-capped AB_3 tetramers assembled by $S_{2090}V_{380}$ micelles (A) and 50-nm silica NPs (B) in A:B ≈ 1:3.

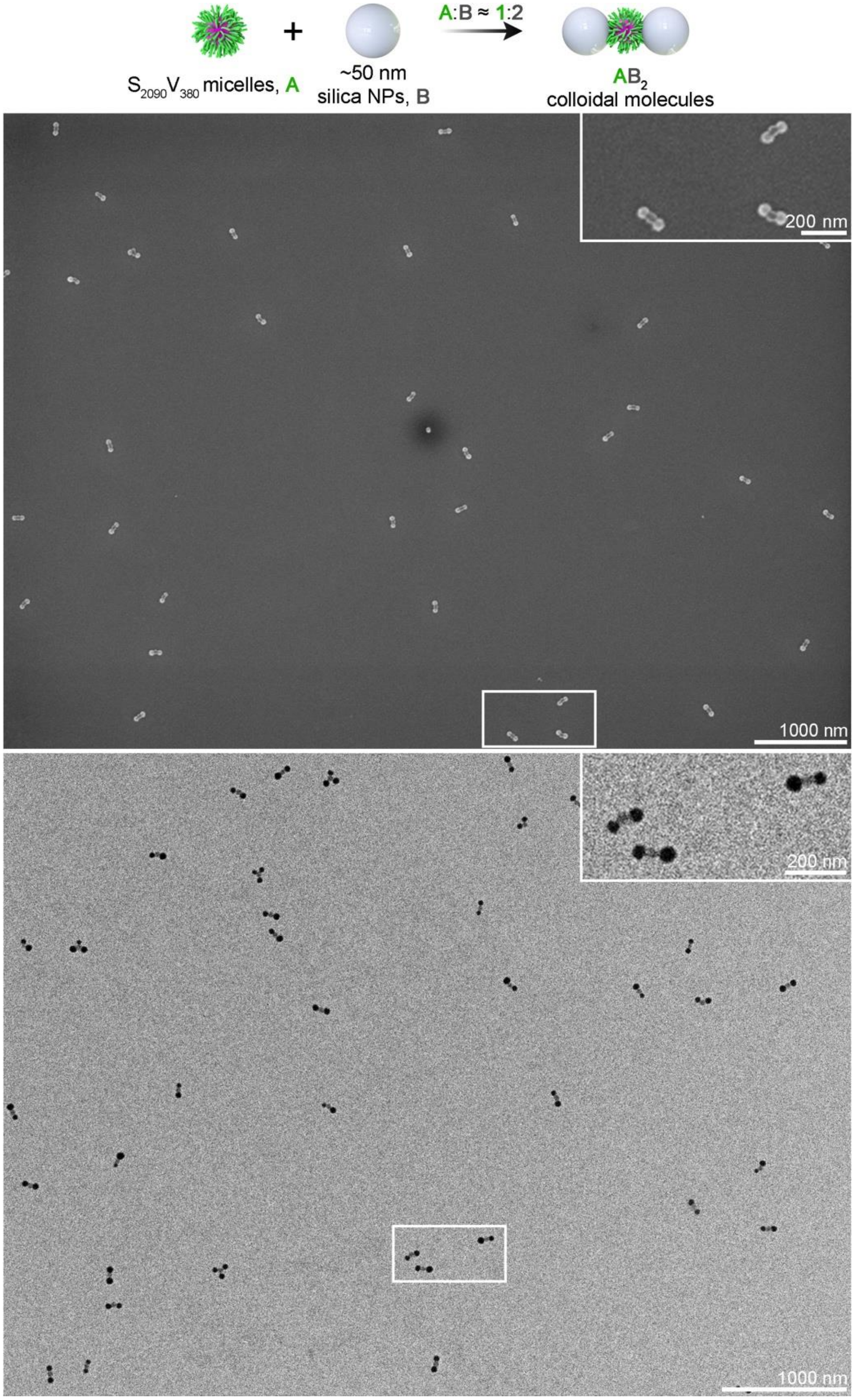

Supplementary Figure 6. Wide-field SEM and TEM images of silica-NPs-capped AB_2 trimers assembled by $S_{2090}V_{380}$ micelles (A) and 50-nm silica NPs (B) in A:B ≈ 1:2.

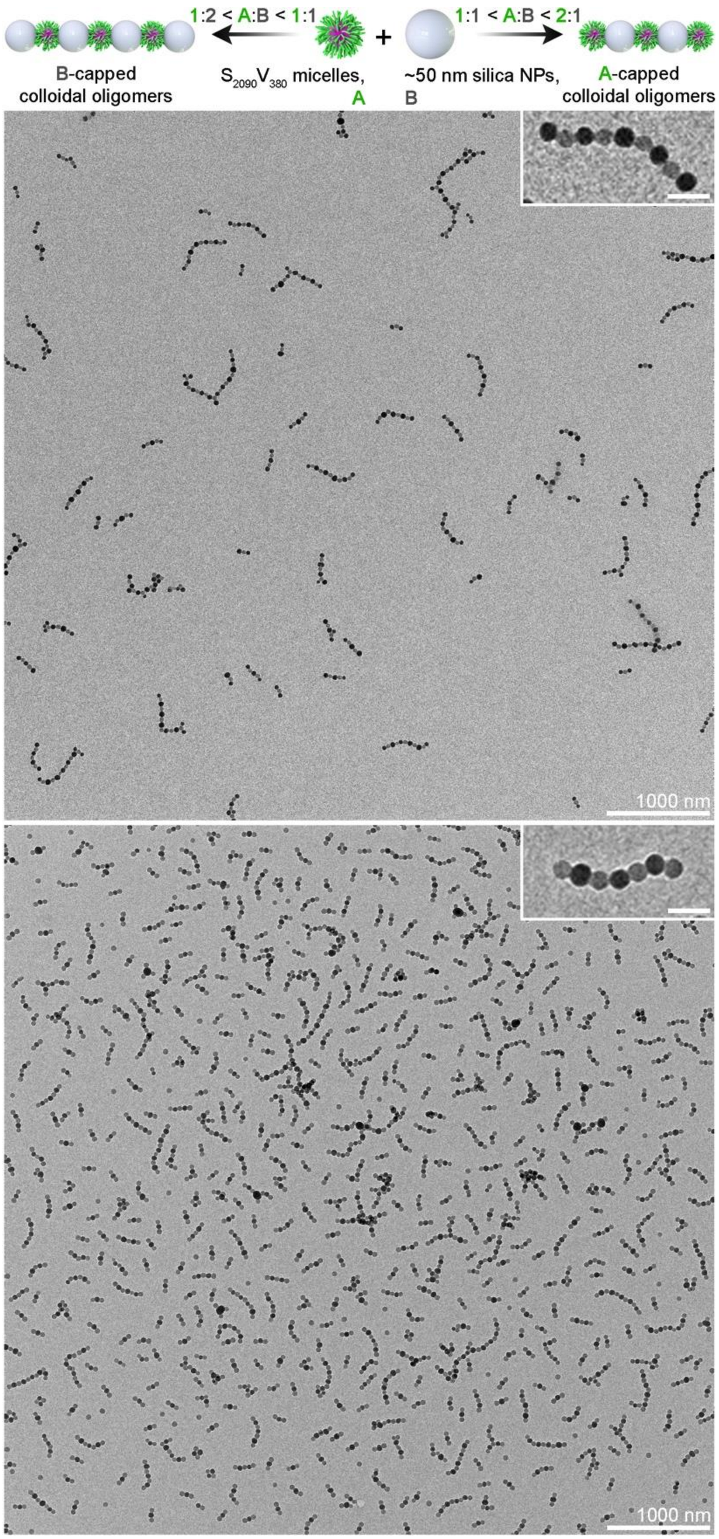

Supplementary Figure 7. Switchable end-capping of 1D alternating colloidal copolymers. Wide-field TEM images of colloidal oligomers end-capped by 50-nm silica NPs (1:2 < A:B < 1:1, top) and $S_{2090}V_{380}$ micelles (1:1 < A:B < 2:1, bottom). Inset scale bars = 100 nm.

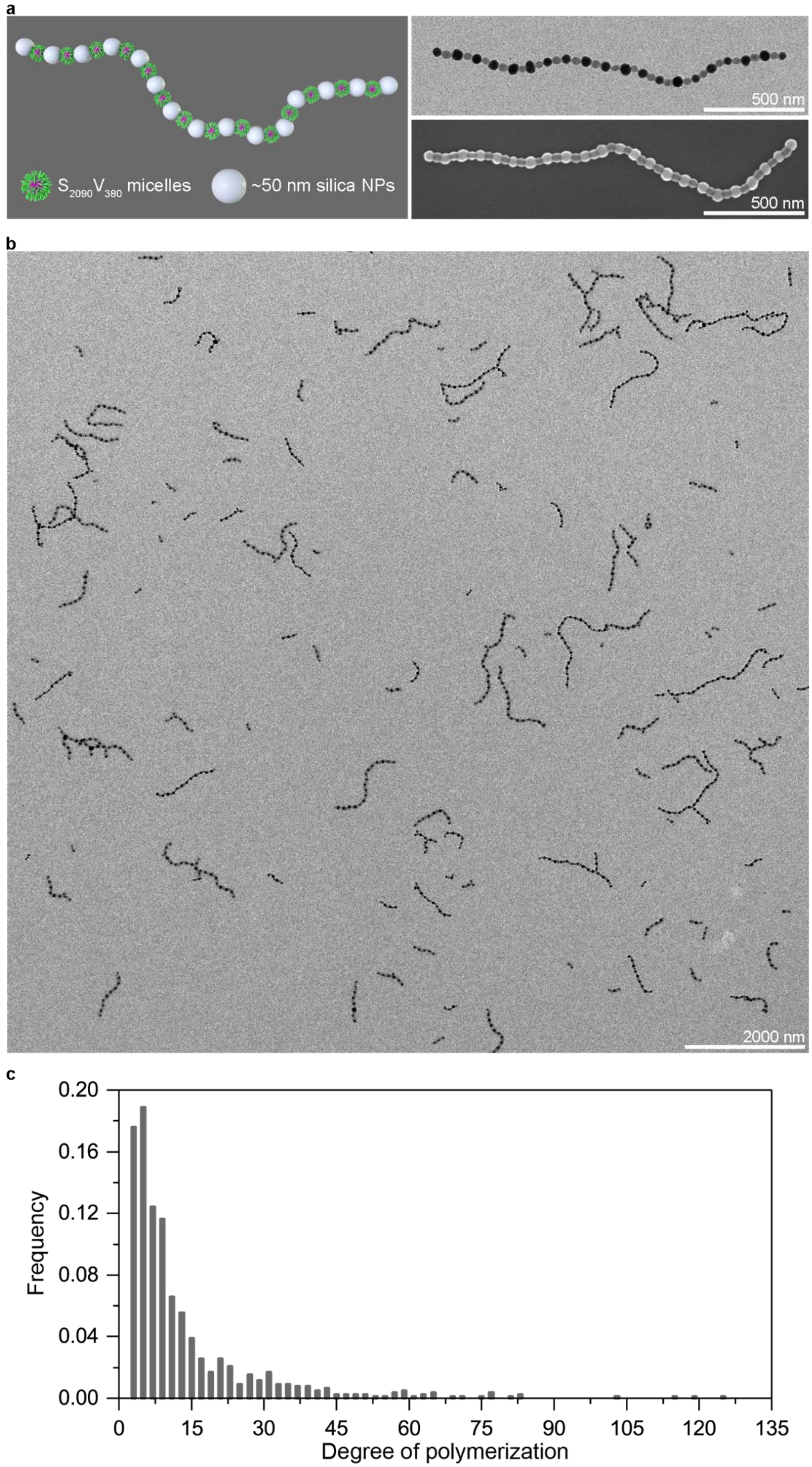
a
S2090V380 micelles
~50 nm silica NPs
500 nm
500 nm
b
2000 nm
c
Frequency
Degree of polymerization
0.00
0.04
0.08
0.12
0.16
0.20
0
15
30
45
60
75
90
105
120
135

Supplementary Figure 8. 1D alternating colloidal copolymers assembled by $S_{2090}V_{380}$ micelles (A) and 50-nm silica NPs (B) in A:B ≈ 1:1. (**a**) Schematic illustration and representative TEM and SEM images of the alternating colloidal copolymers. (**b**) Wide-field TEM image of the assembled alternating colloidal copolymers. It should be noted that a considerable number of the resultant colloidal polymers are slightly branched with arms emanating from some $S_{2090}V_{380}$ micelle units. (**c**) Frequency distributions (gray bars) of the degree of polymerization of the assembled colloidal copolymers.

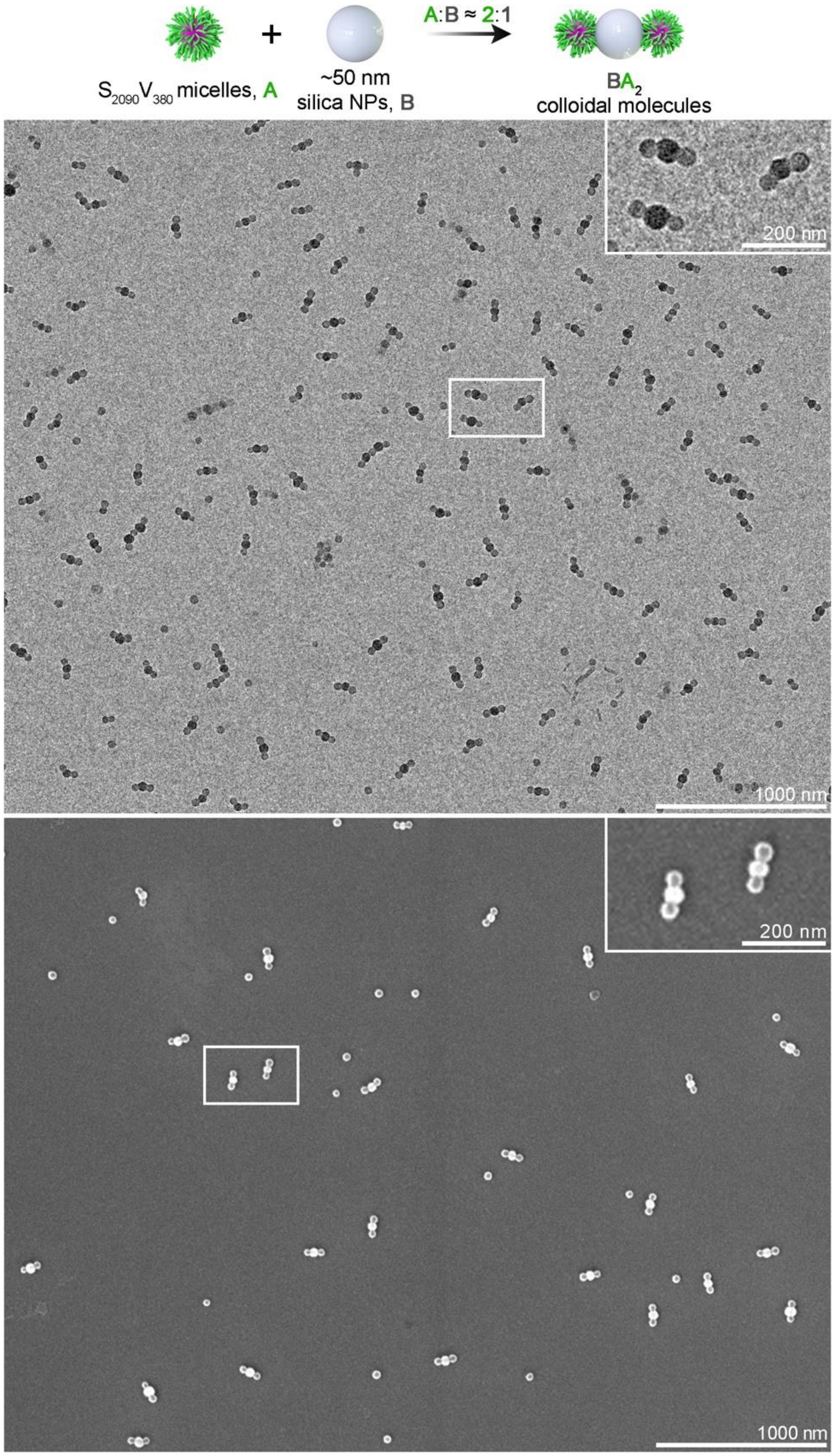

Supplementary Figure 9. Wide-field TEM and SEM images of micelles-capped BA_2 trimers assembled by $S_{2090}V_{380}$ micelles (A) and 50-nm silica NPs (B) in A:B ≈ 2:1.

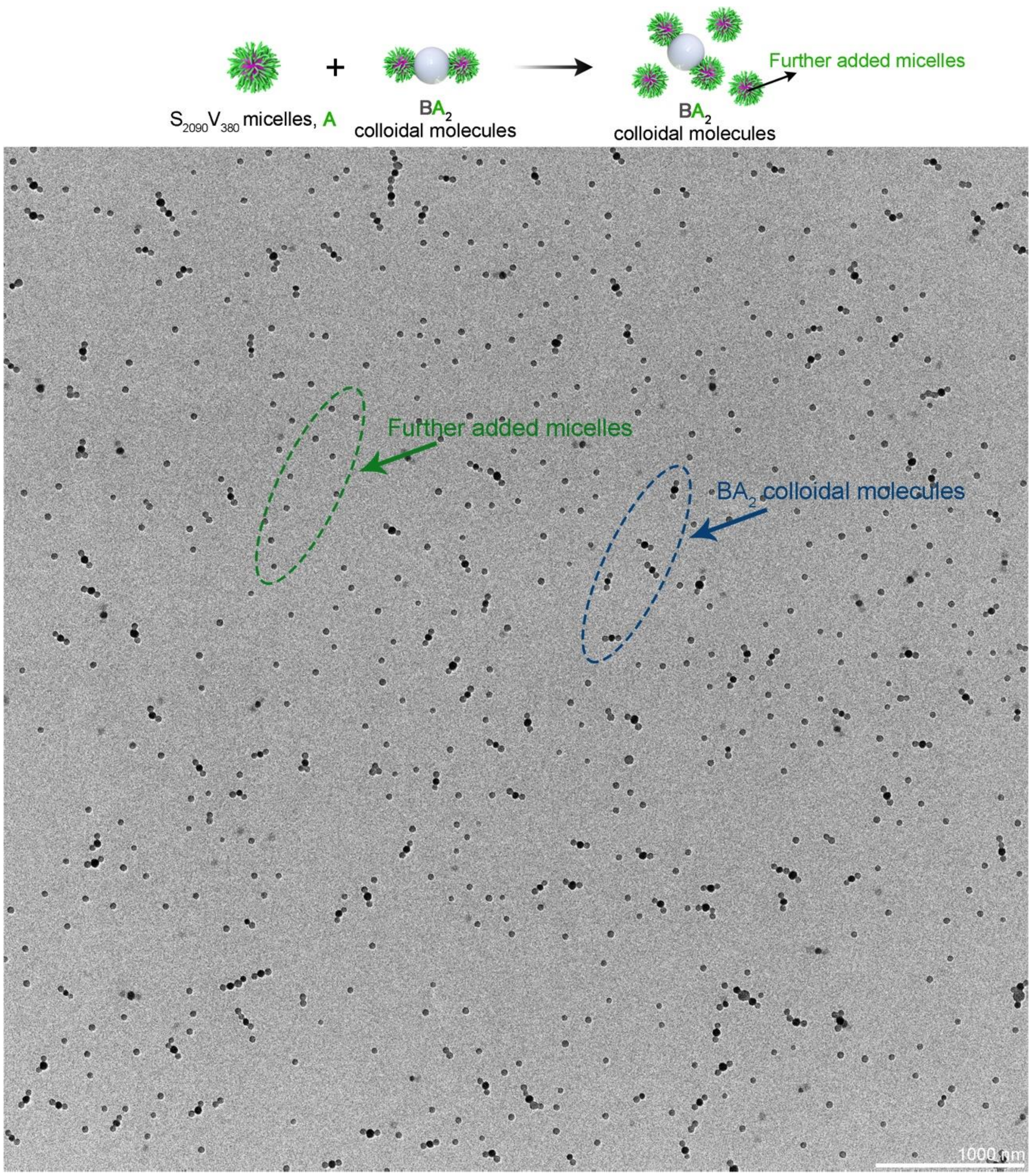

Supplementary Figure 10. Wide-field TEM image of a mixture of assembled BA_2 trimers and further added $S_{2090}V_{380}$ micelles. The experiment was performed by the further addition of 50 μL of an ethanol solution of $S_{2090}V_{380}$ micelles into 360 μL of an ethanol solution of the as-prepared micelles-capped BA_2 trimers (assembled by $S_{2090}V_{380}$ micelles and 50-nm silica NPs, Supplementary Fig. 9). The preformed BA_2 trimers retained intact and the further added $S_{2090}V_{380}$ micelles failed to associate with them, indicating that the surface of the central silica NP is saturated due to the coverage of the P2VP coronas.

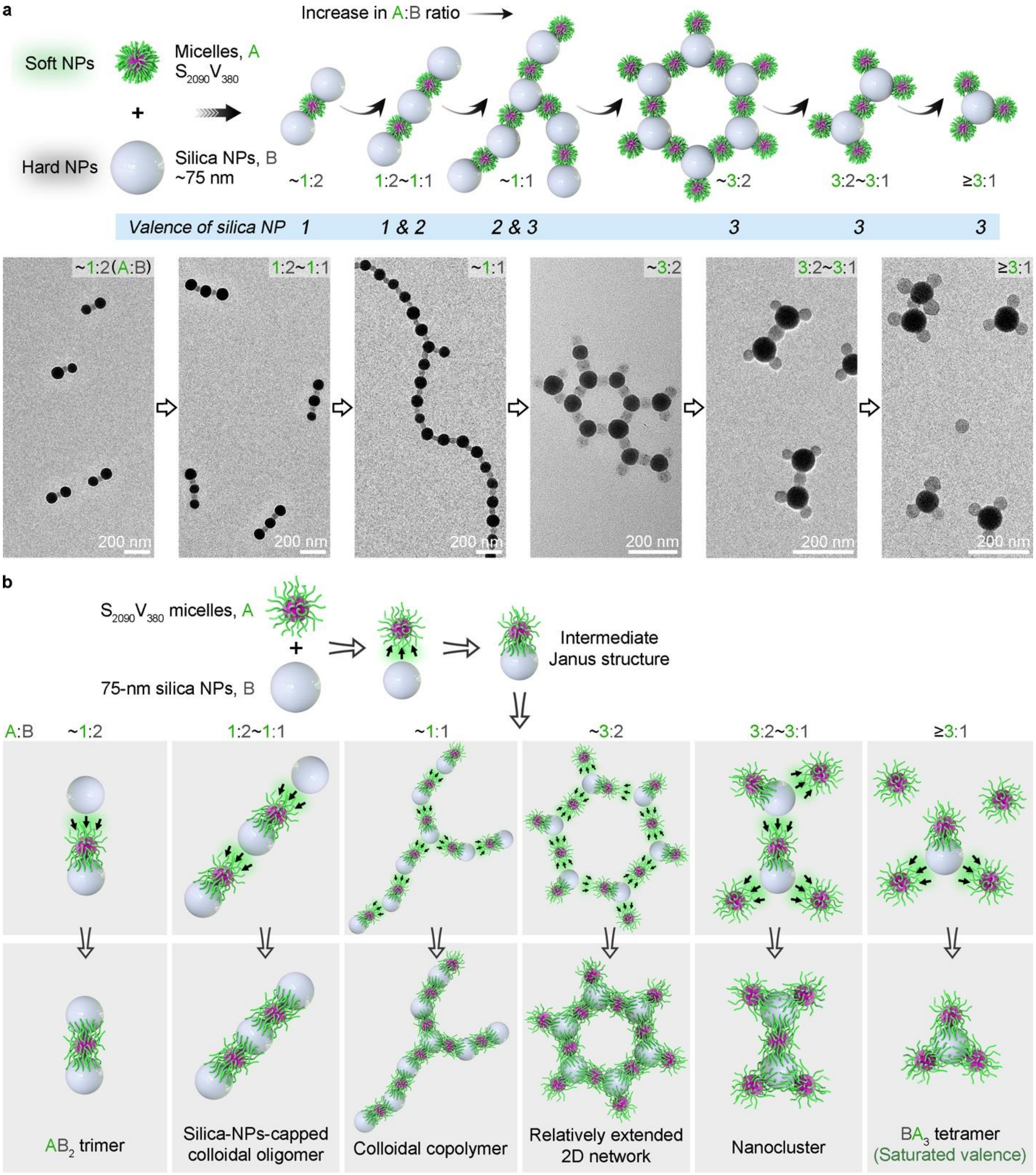

Supplementary Figure 11. Association of $S_{2090}V_{380}$ micelles and 75-nm silica NPs in various A:B feeding ratios. (**a**) General trend of the co-assembly of $S_{2090}V_{380}$ micelles and 75-nm silica NPs in various A:B ratios. The valence of silica NP means the number of surrounding, actually associated soft NPs. TEM images show the assembled colloidal architectures in different A:B ratios. (**b**) Schematic illustration for the possible association process of $S_{2090}V_{380}$ micelles and 75-nm silica NPs in various A:B ratios.

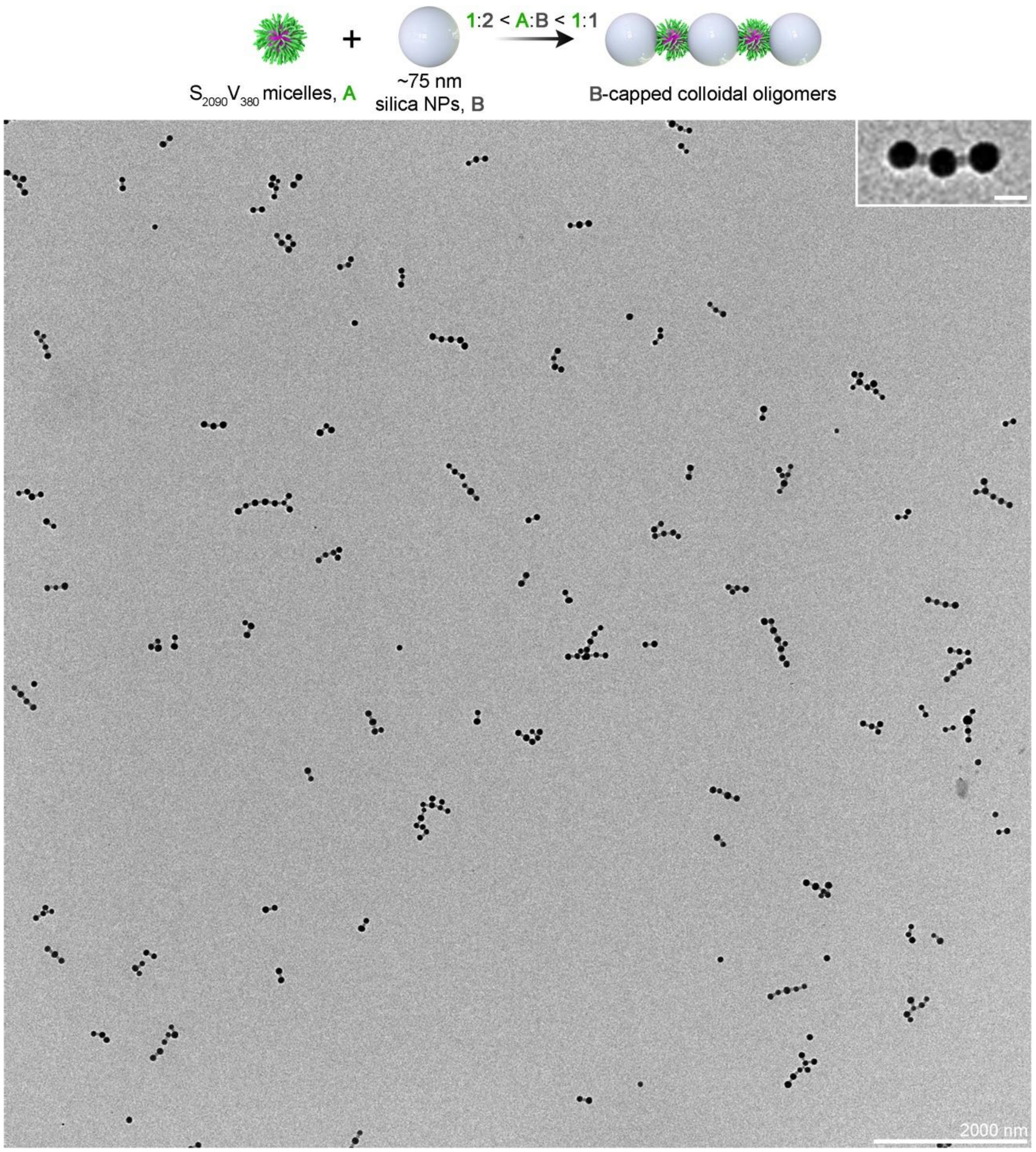

Supplementary Figure 12. Wide-field TEM image of silica-NPs-capped colloidal oligomers assembled by $S_{2090}V_{380}$ micelles (A) and 75-nm silica NPs (B) in 1:2 < A:B < 1:1. Inset scale bars = 100 nm.

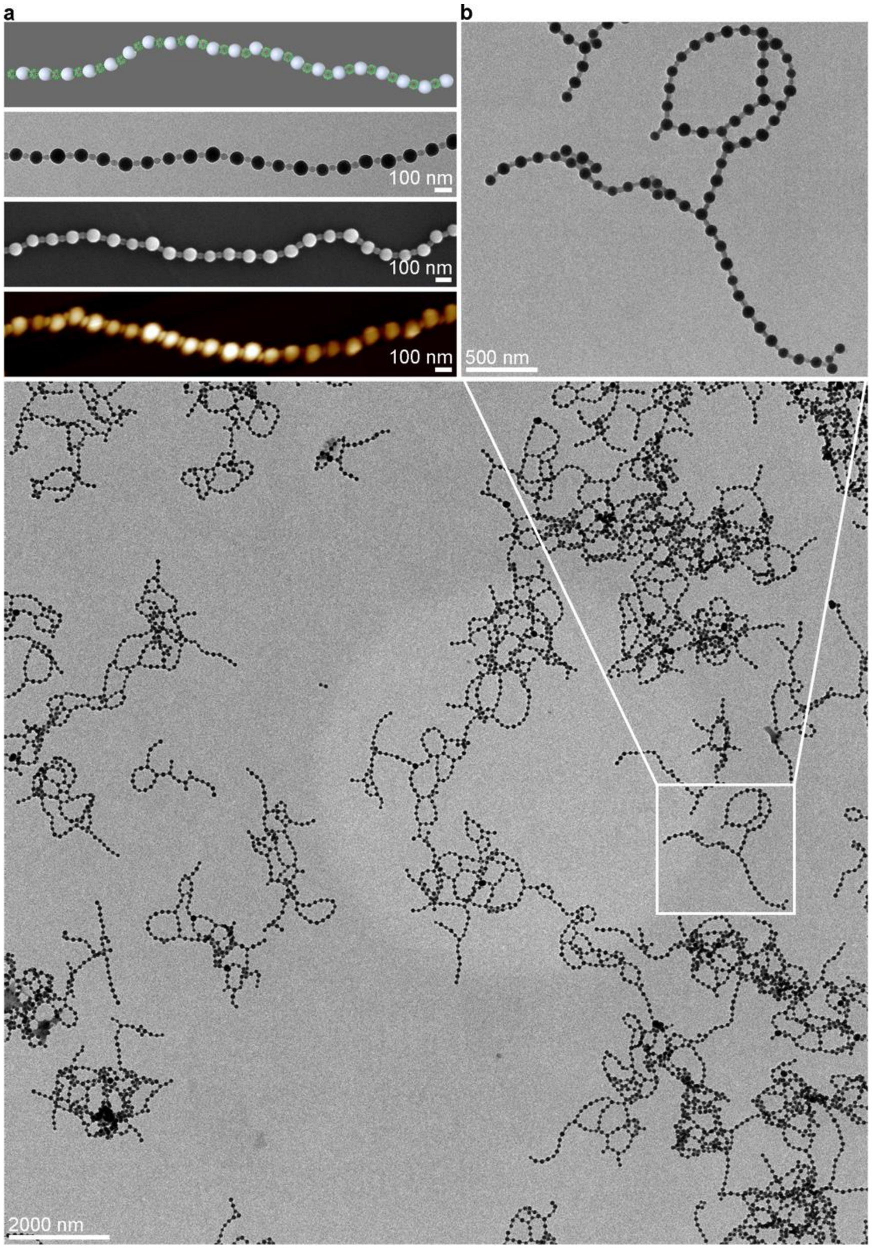

Supplementary Figure 13. 1D alternating colloidal copolymers assembled by $S_{2090}V_{380}$ micelles (A) and 75-nm silica NPs (B) in A:B ≈ 1:1. (**a**) Schematic illustration and representative TEM, SEM and AFM height images of alternating colloidal copolymers. (**b**) Wide-field TEM image of the assembled alternating colloidal copolymers. It should be noted that the resultant colloidal polymers are predominantly branched with arms emanating from the 75-nm silica NP units.

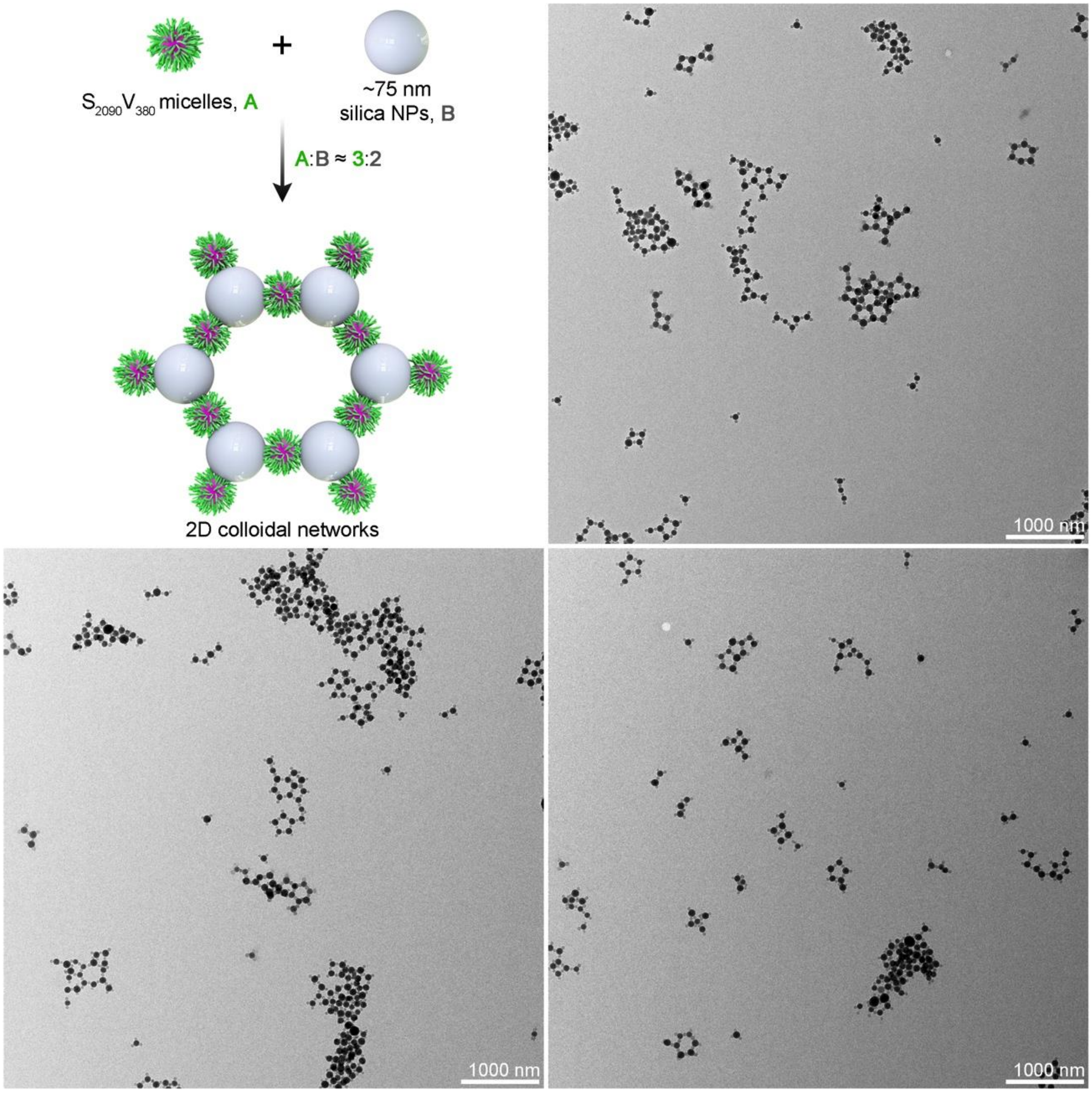

Supplementary Figure 14. Wide-field TEM images of relatively extended 2D networks assembled by $S_{2090}V_{380}$ micelles (A) and 75-nm silica NPs (B) in A:B ≈ 3:2.

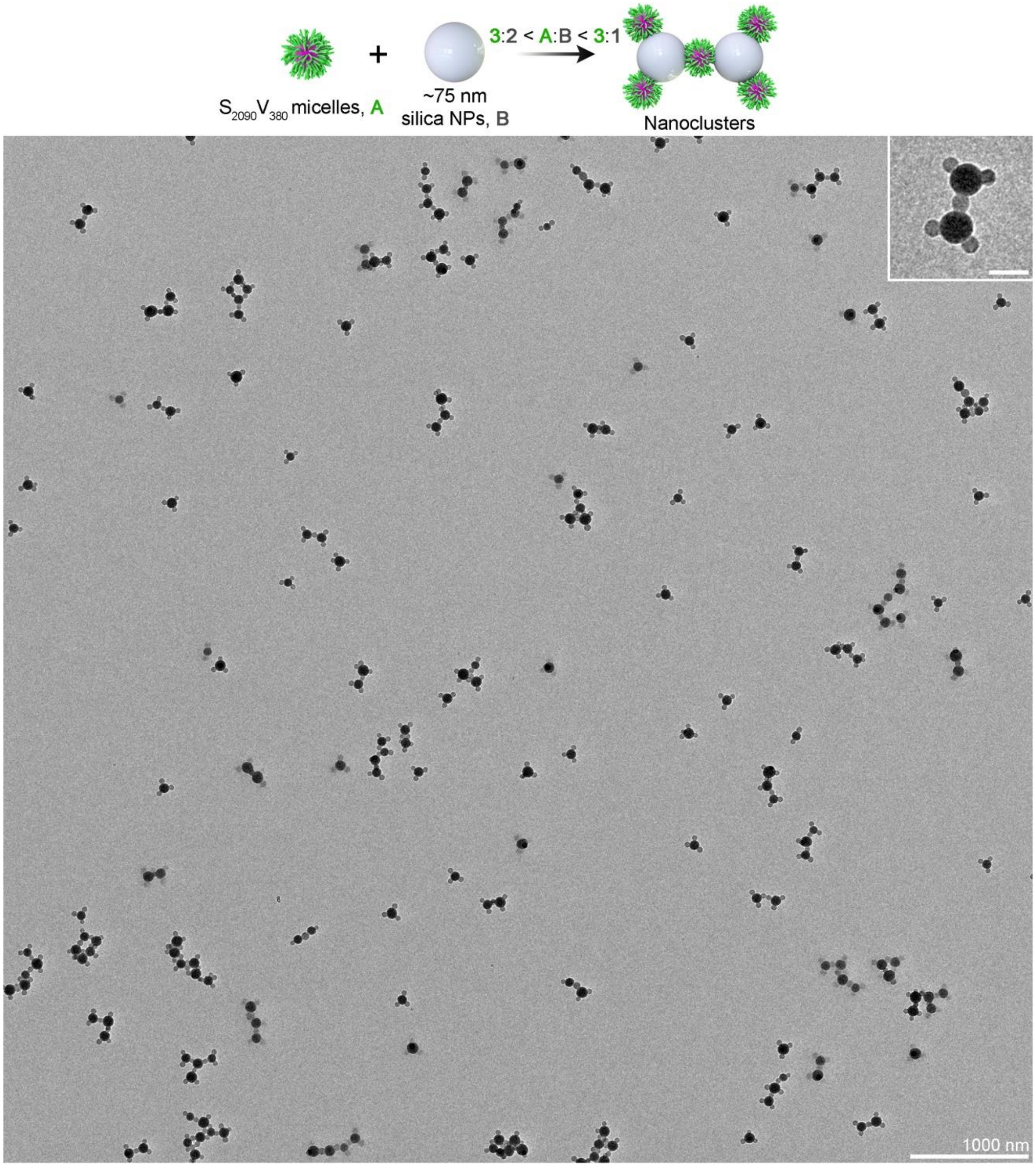

Supplementary Figure 15. Wide-field TEM image of micelles-capped nanoclusters assembled by $S_{2090}V_{380}$ micelles (A) and 75-nm silica NPs (B) in 3:2 < A:B < 3:1. Inset scale bars = 100 nm.

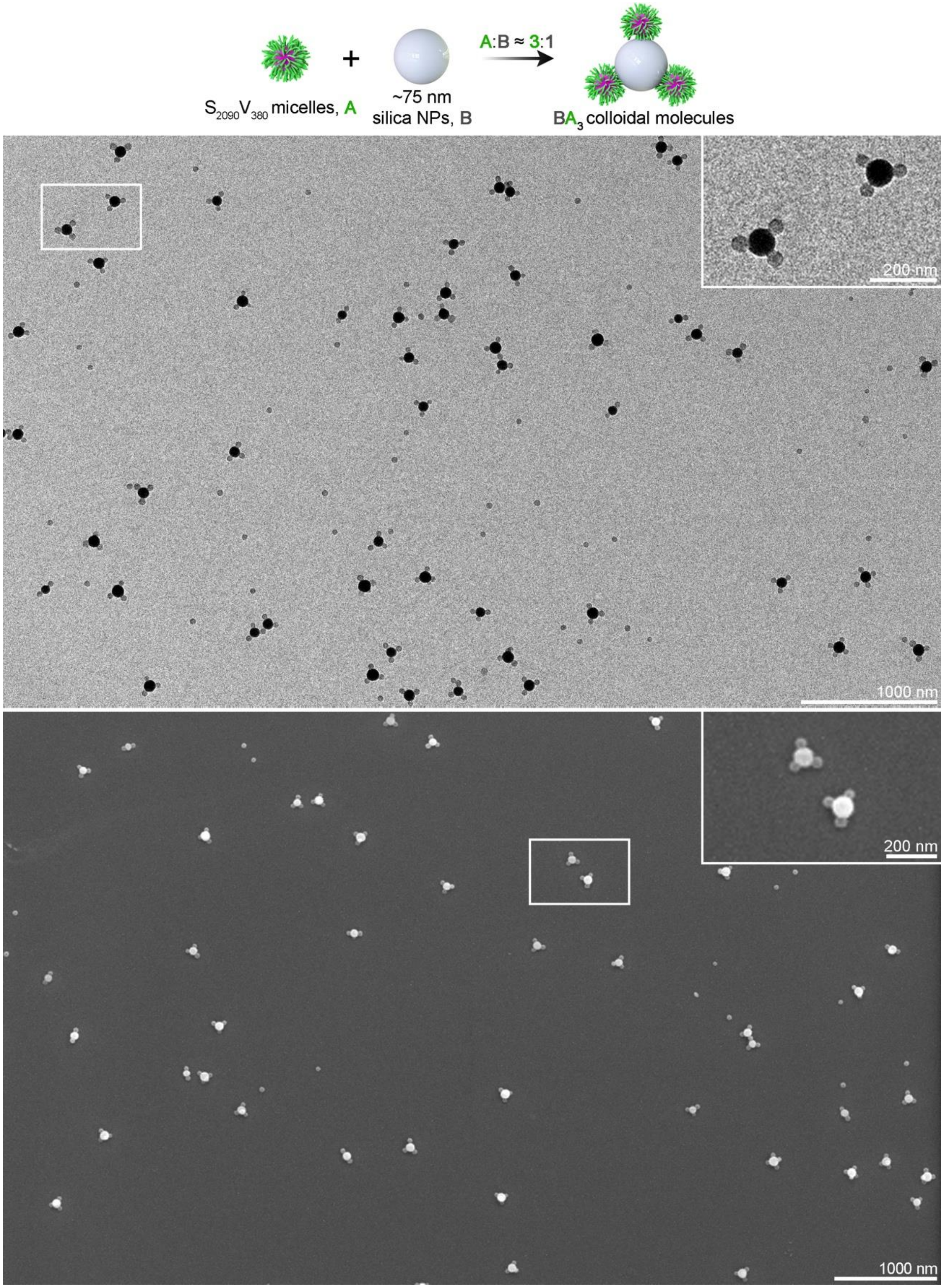

Supplementary Figure 16. Wide-field TEM and SEM images of micelles-capped BA_3 tetramers assembled by $S_{2090}V_{380}$ micelles (A) and 75-nm silica NPs (B) in A:B ≈ 3:1.

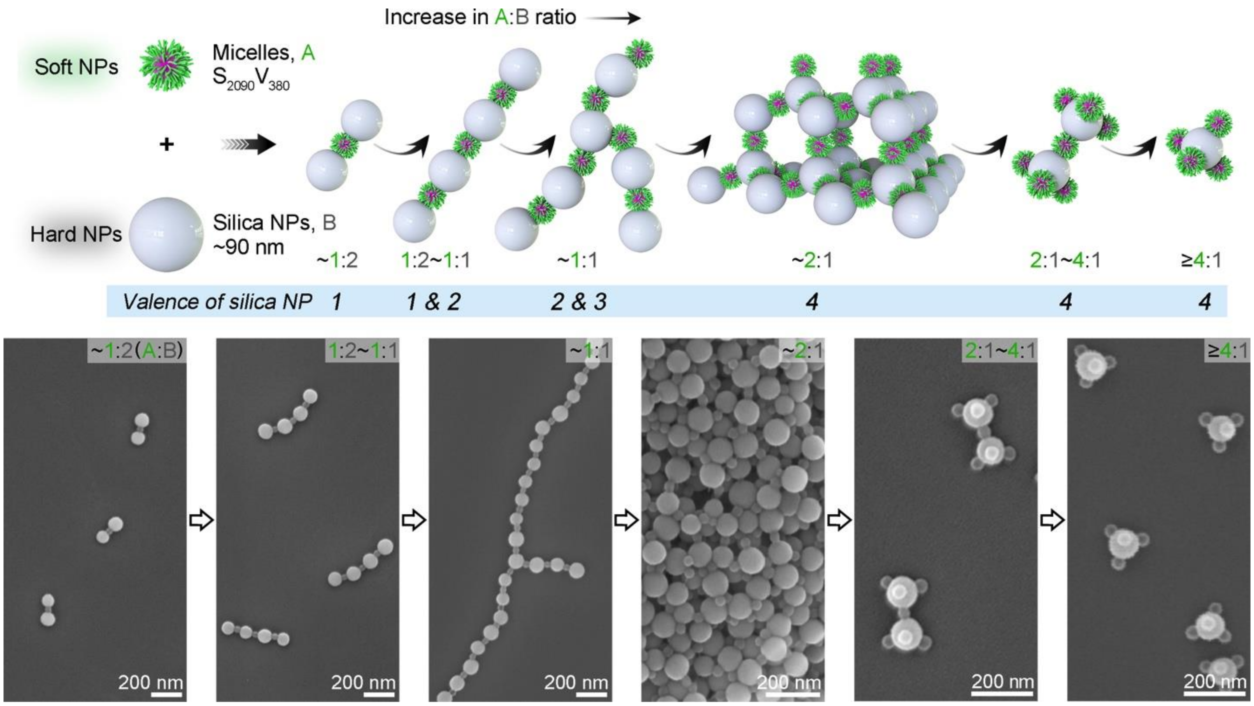

Supplementary Figure 17. Association of $S_{2090}V_{380}$ micelles and 90-nm silica NPs in various A:B feeding ratios. Top: Schematic illustration of the general trend of the co-assembly of $S_{2090}V_{380}$ micelles and 90-nm silica NPs in various A:B ratios. The valence of silica NP means the number of surrounding, actually associated soft NPs. **Below:** SEM images of the assembled colloidal architectures in different A:B ratios.

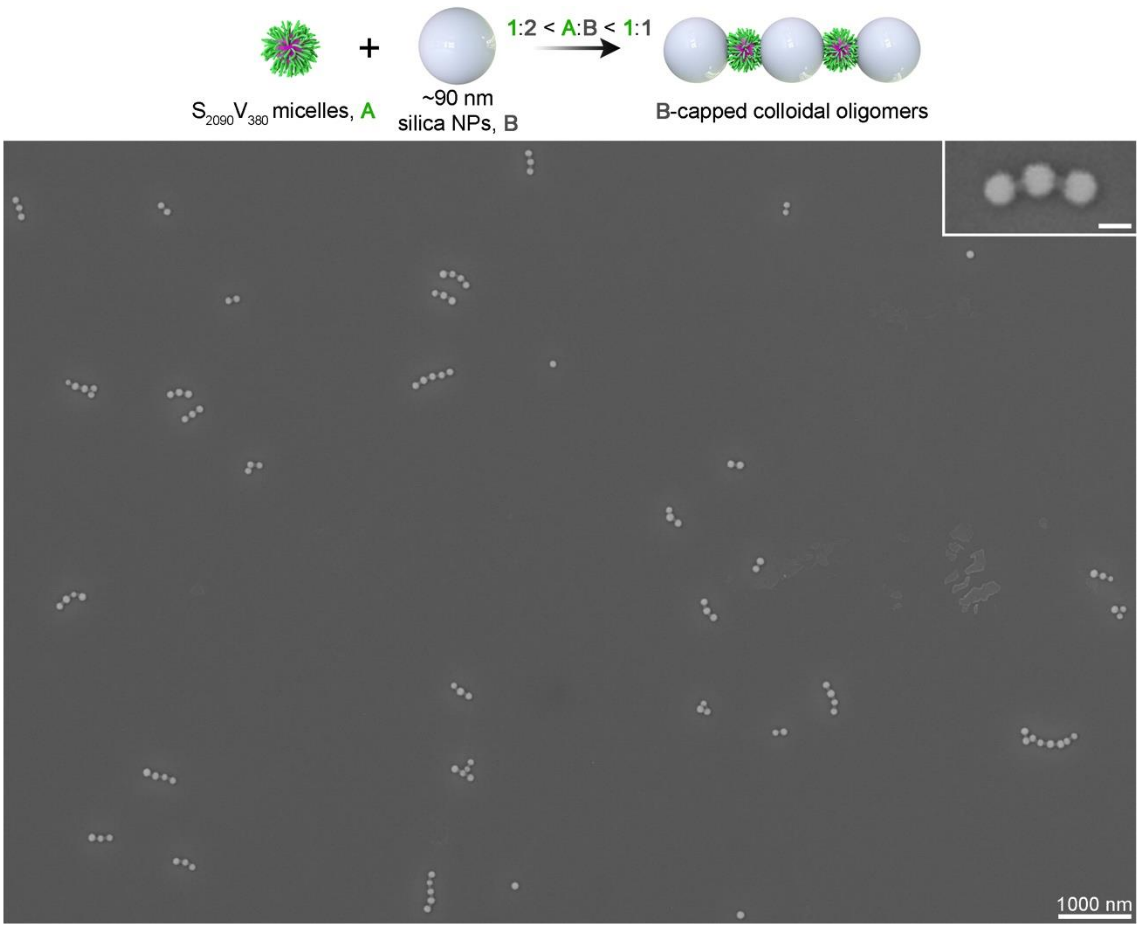

Supplementary Figure 18. Wide-field SEM image of silica-NPs-capped colloidal oligomers assembled by $S_{2090}V_{380}$ micelles (A) and 90-nm silica NPs (B) in 1:2 < A:B < 1:1. Inset scale bars = 100 nm.

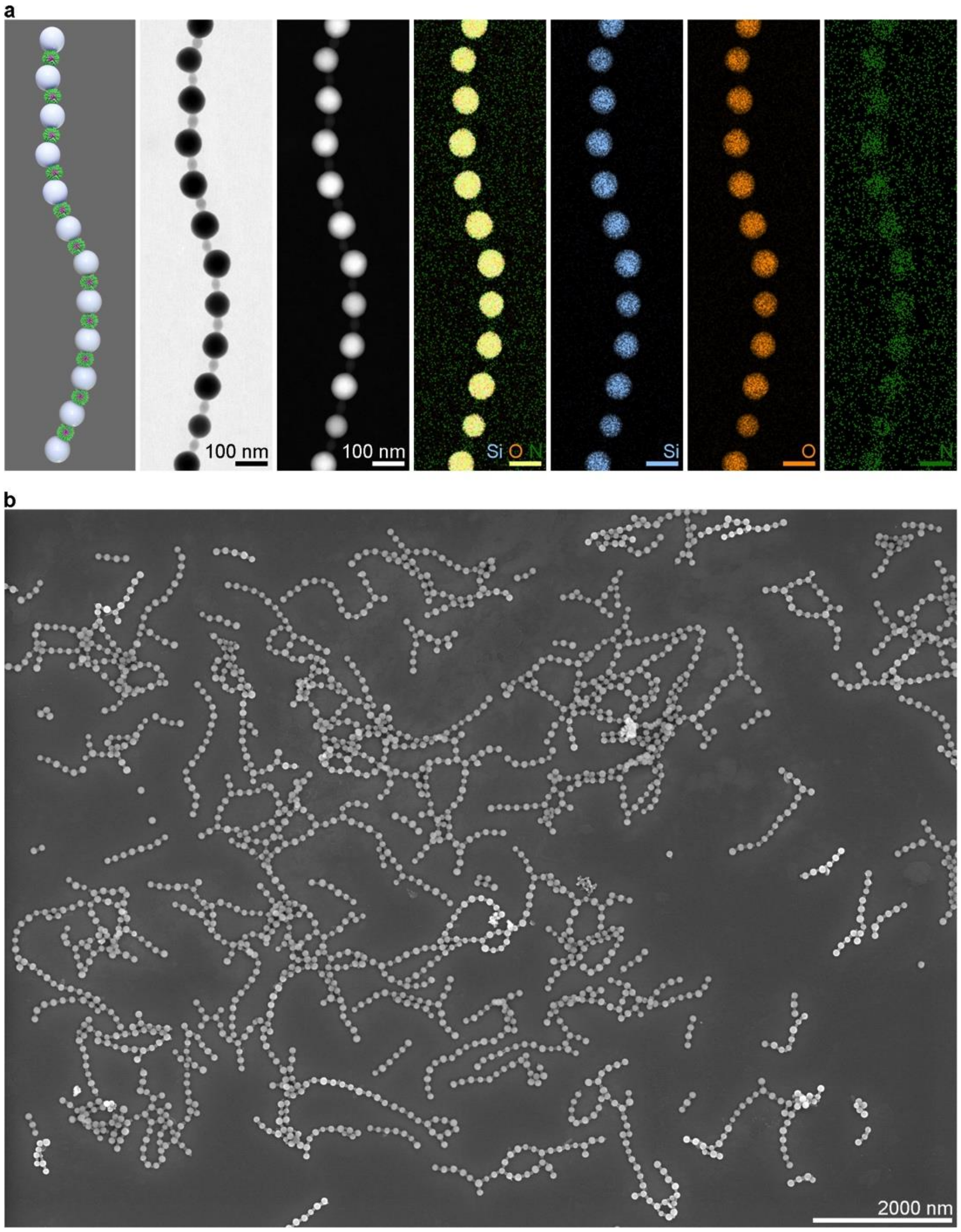

Supplementary Figure 19. 1D alternating colloidal copolymers assembled by $S_{2090}V_{380}$ micelles (A) and 90-nm silica NPs (B) in A:B ≈ 1:1. (**a**) Schematic illustration and representative BF-STEM, HAADF-STEM and corresponding EDS elemental mapping images (Si is colored in blue, O in orange, and N in green) of alternating colloidal copolymers. (**b**) Wide-field SEM image of the assembled alternating colloidal copolymers. It should be noted that the resultant colloidal polymers are predominantly branched with arms emanating from the 90-nm silica NP units.

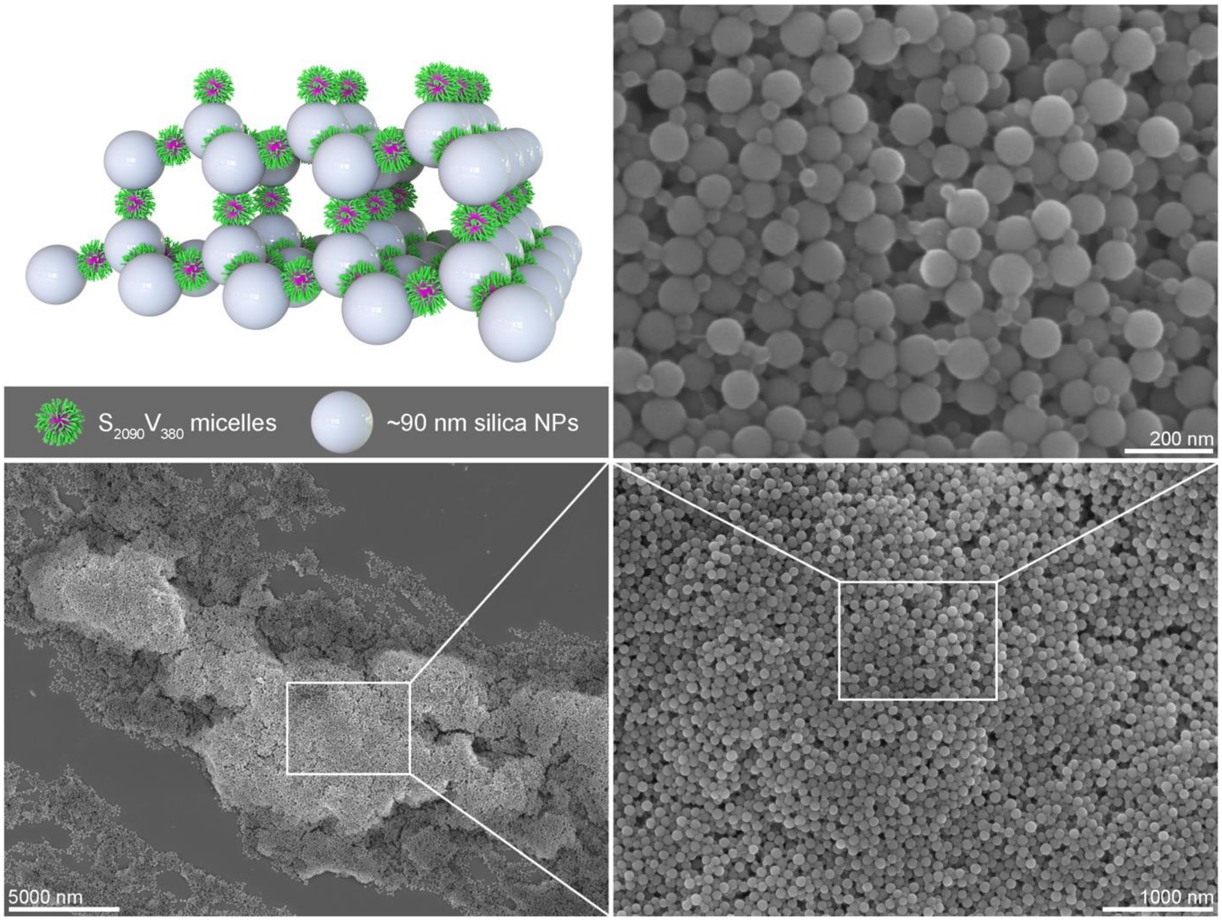

Supplementary Figure 20. Wide-field SEM images of large-scale 3D stacked monoliths assembled by $S_{2090}V_{380}$ micelles (A) and 90-nm silica NPs (B) in A:B ≈ 2:1.

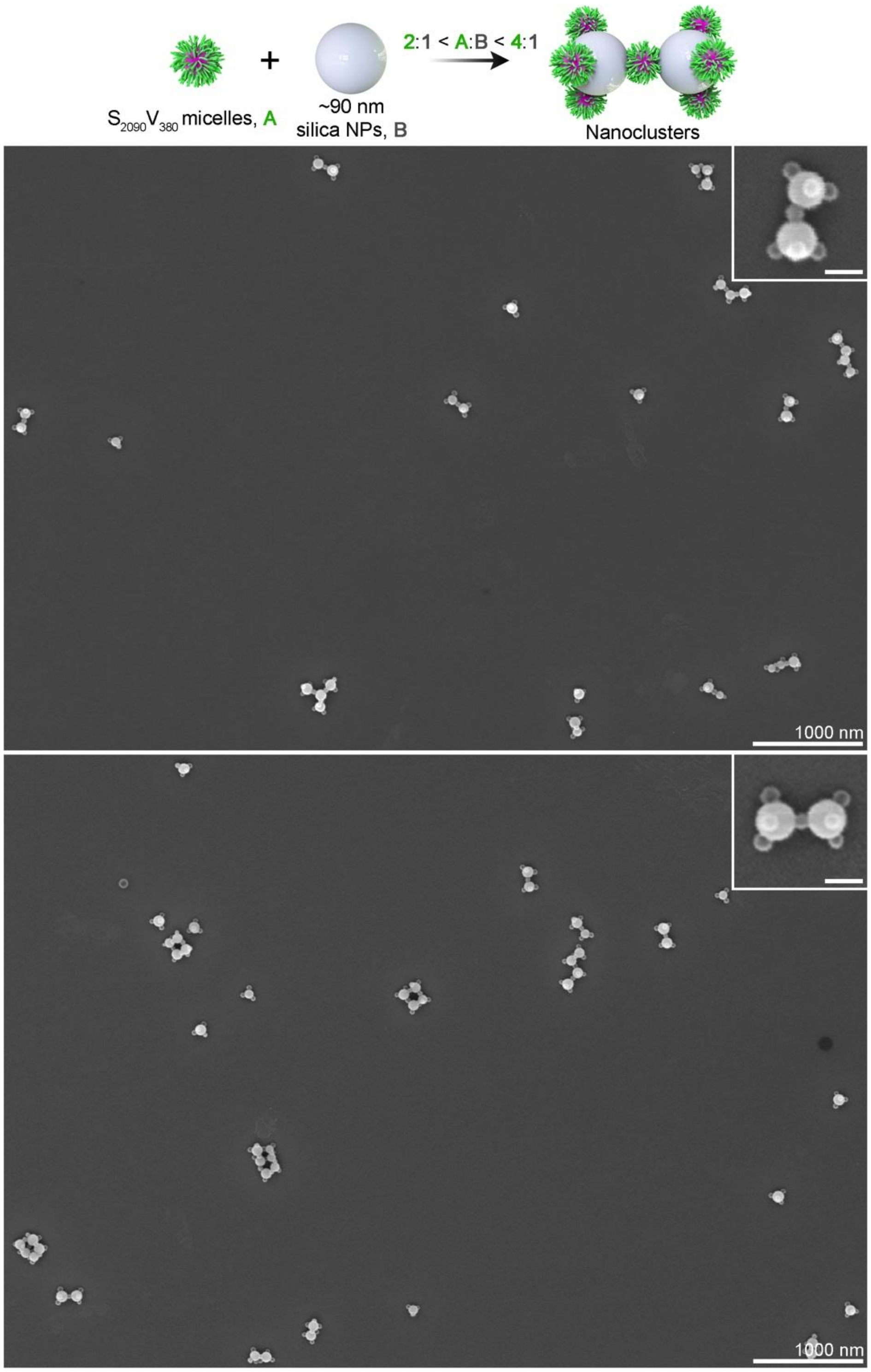

Supplementary Figure 21. Wide-field SEM images of micelles-capped nanoclusters assembled by $S_{2090}V_{380}$ micelles (A) and 90-nm silica NPs (B) in 2:1 < A:B < 4:1. Inset scale bars = 100 nm.

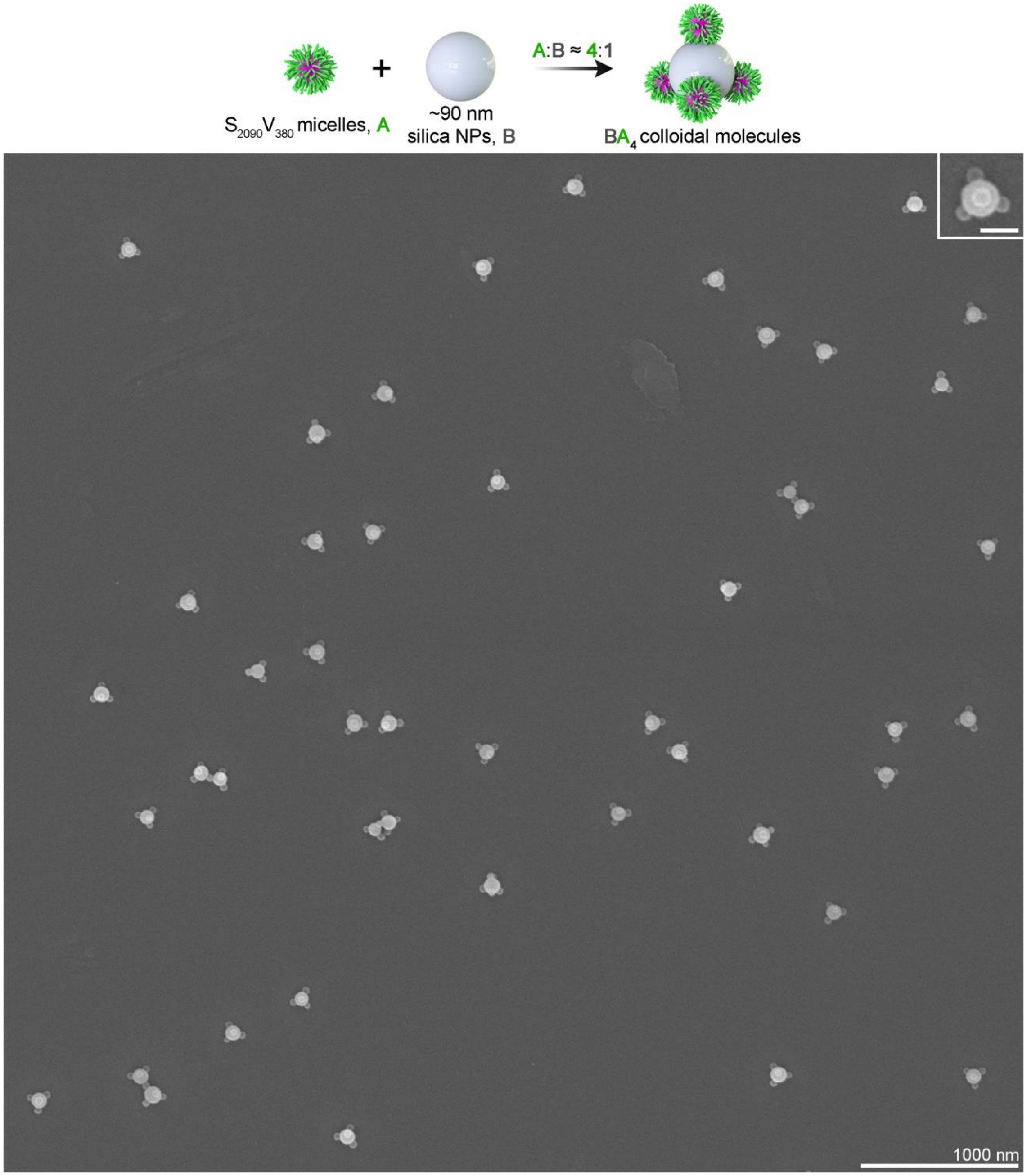

Supplementary Figure 22. Wide-field SEM image of micelles-capped BA_4 pentamers assembled by $S_{2090}V_{380}$ micelles (A) and 90-nm silica NPs (B) in A:B ≈ 4:1. Inset scale bars = 100 nm.

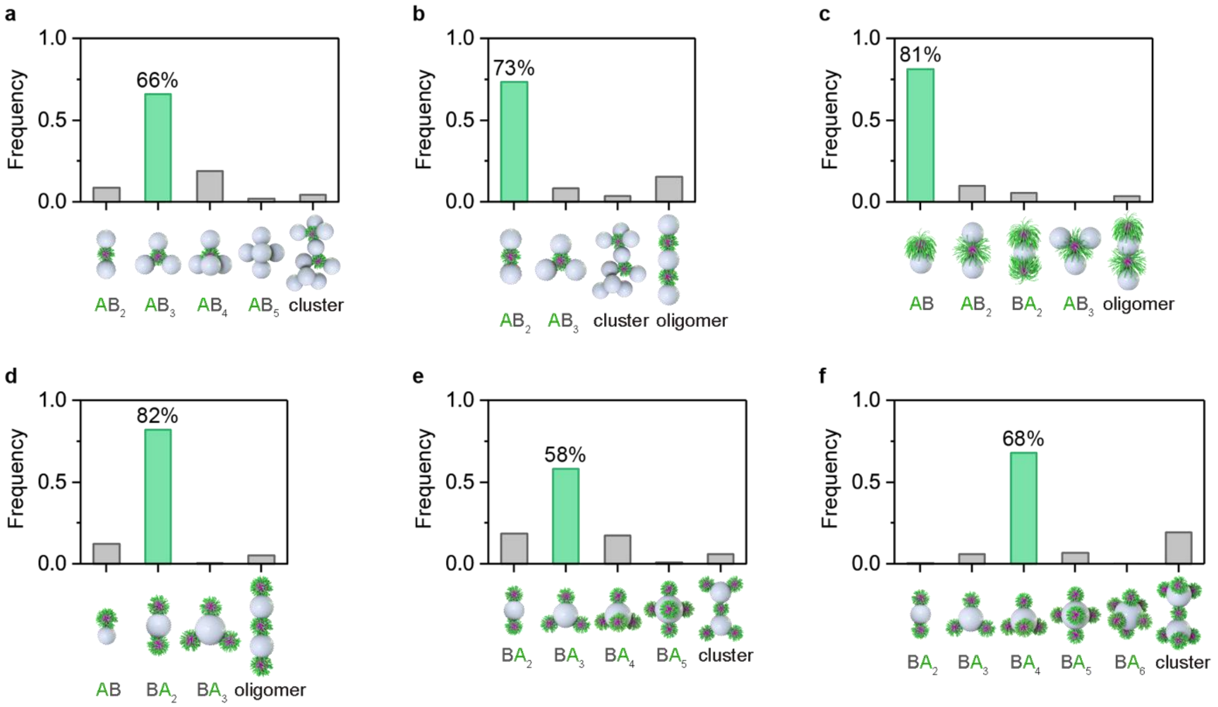

Supplementary Figure 23. Statistical analysis of the assembled colloidal architectures. (**a**) Silica-NPs-capped AB_3 tetramers assembled by $S_{2090}V_{380}$ micelles and 50-nm silica NPs (corresponding to Supplementary Fig. 5). (**b**) Silica-NPs-capped AB_2 trimers assembled by $S_{2090}V_{380}$ micelles and 50-nm silica NPs (corresponding to Supplementary Fig. 6). (**c**) AB dimers assembled by $S_{2000}V_{1910}$ micelles and 50-nm silica NPs (corresponding to Supplementary Fig. 28). (**d**) Micelles-capped BA_2 trimers assembled by $S_{2090}V_{380}$ micelles and 50-nm silica NPs (corresponding to Supplementary Fig. 9). (**e**) Micelles-capped BA_3 tetramers assembled by $S_{2090}V_{380}$ micelles and 75-nm silica NPs (corresponding to Supplementary Fig. 16). (**f**) Micelles-capped BA_4 pentamers assembled by $S_{2090}V_{380}$ micelles and 90-nm silica NPs (corresponding to Supplementary Fig. 22).

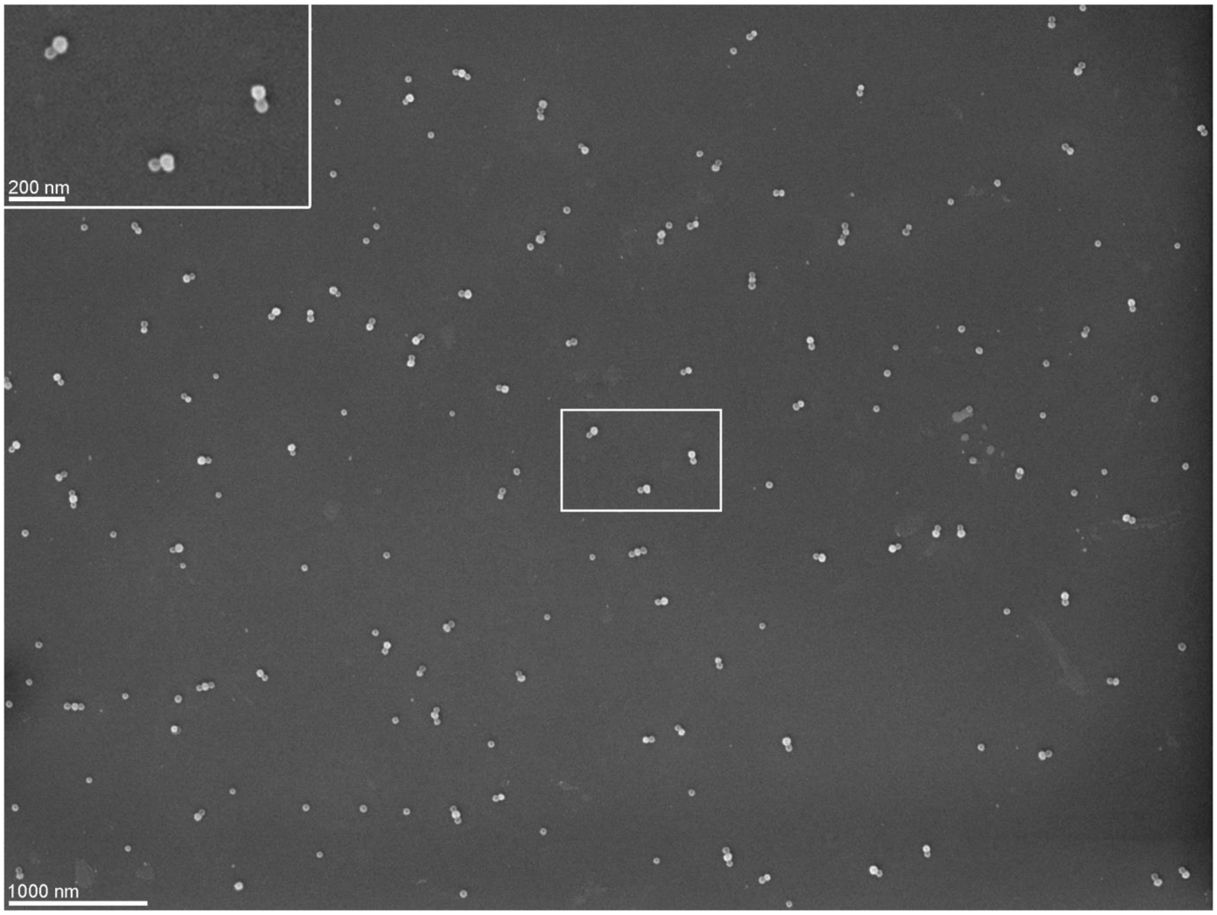

Supplementary Figure 24. Wide-field SEM image of intermediate Janus structures formed at the initial stage of association. $S_{2090}V_{380}$ micelles (A) and 50-nm silica NPs (B) was mixed in A:B ≈ 2:1, and after 30 s one drop (ca. 10 μL) of the mixture was quickly placed on a clean silicon wafer and blown dry under a stream of nitrogen to capture the intermediate products at the initial stage of assembly.

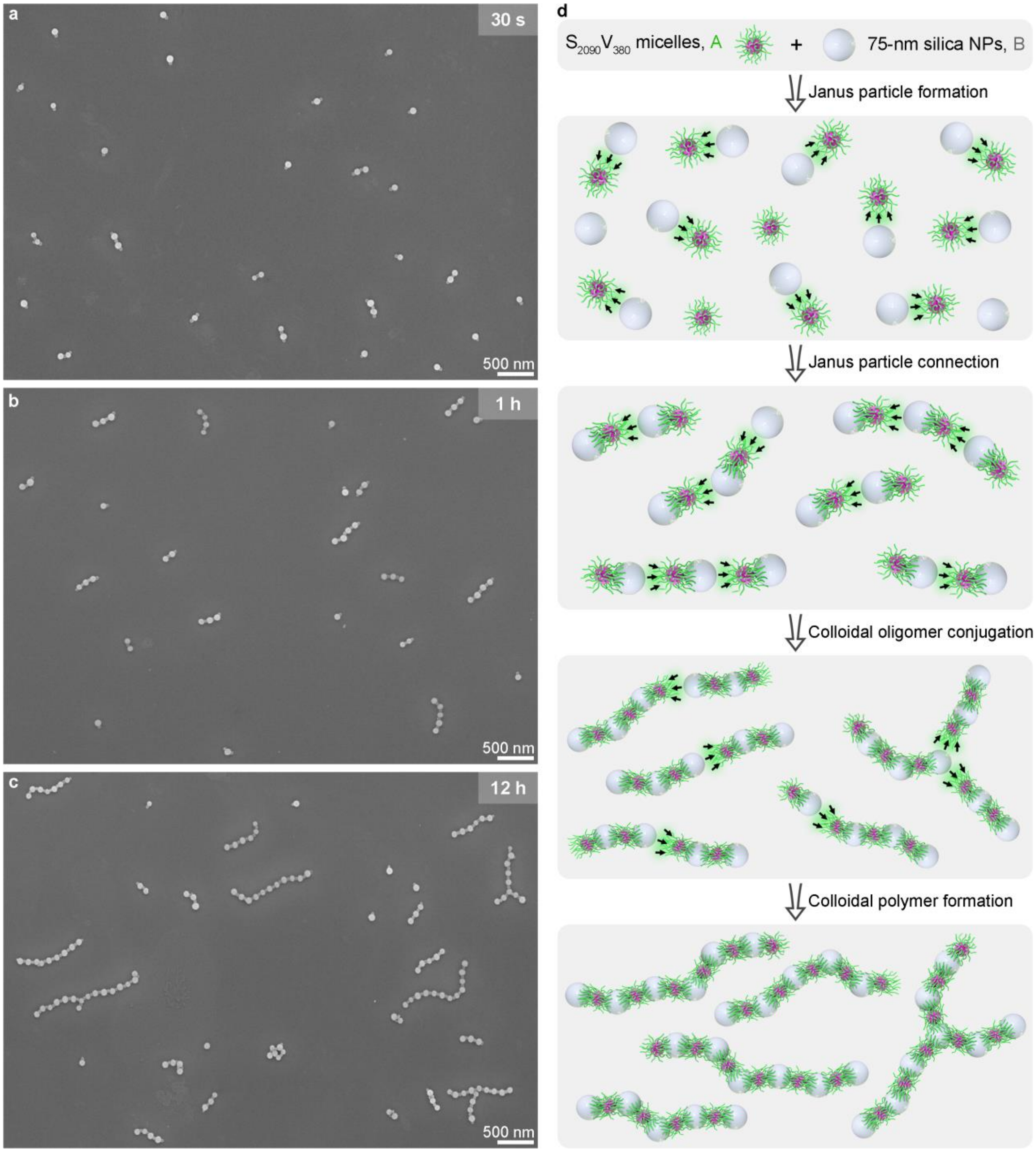

Supplementary Figure 25. Formation process of colloidal copolymers. (**a** to **c**) SEM images of time-dependent growth of colloidal polymers composed of $S_{2090}V_{380}$ micelles and 75-nm silica NPs at different times of (**a**) 30 s, (**b**) 1 h, and (**c**) 12 h. (**d**) Proposed formation mechanism of colloidal polymers which involves rapid formation of intermediate Janus structures, subsequent connection of Janus intermediates, and further conjugation of colloidal oligomers. It seems that the growth of colloidal polymers takes a step-wise mode. After the rapid consuming of SV micelles and silica NPs to generate intermediate Janus particles (within 30 s), Janus particles connect with each other to form colloidal oligomers and these oligomers can further conjugate with each other to achieve higher degree of polymerization (like typical alternating condensation polymerization).

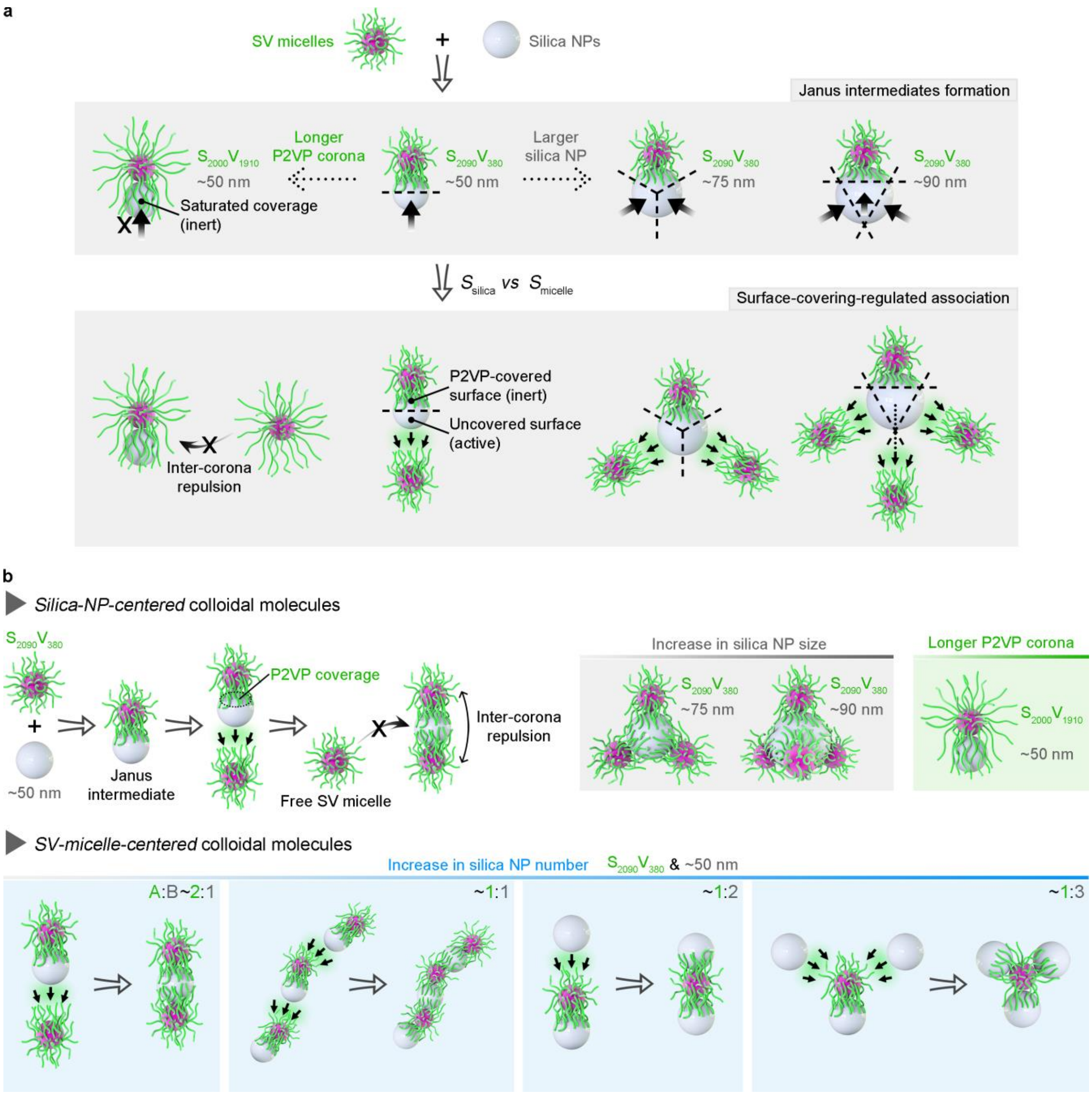

Supplementary Figure 26. Proposed mechanism and design principle for the determination of different colloidal architectures. (**a**) Proposed mechanism for the determination of saturated valence of silica-NP-centered colloidal molecules. (**b**) Additional insights to the formation of silica-NP-centered and SV-micelle-centered colloidal molecules. The structure (valence) of the colloidal molecules is generally regulated by the relative size and feeding ratio of SV micelles (denoted as **A**) and hard nanoparticles (NPs, denoted as **B**) (Fig. 2). Taking silica-NP-centered colloidal molecules as an example, the saturated valence ($\boldsymbol{V}_{\mathbf{max}}$) of a silica NP is determined by the surface area of a silica NP ($\boldsymbol{S}_{\mathbf{silica}}$) and the area that a SV micelle can cover on the silica NP ($\boldsymbol{S}_{\mathbf{micelle}}$) (Supplementary Fig. 26a). For example, the saturated valence of a 50-nm silica NP is 2 when $S_{2090}V_{380}$ micelles are employed as evidenced by the co-existence of $\mathbf{BA_2}$ molecules and excessive $S_{2090}V_{380}$ micelles (Supplementary Fig. 10). However, when $S_{2000}V_{1910}$ micelles are used the saturated valence of a 50-nm silica NP turns to be 1 since longer P2VP coronas can cover a larger surface area (Supplementary Figs.

27 and 28). Similarly, the increase in silica NP size (75-nm/90-nm silica NPs, with larger surface area) can increase its saturated valence (Supplementary Figs. 16 and 22). Ideally, the saturated (maximum) valence of a silica NP $\boldsymbol{V}_{\mathbf{max}}$ can be calculated by

$$V_{\text{max}} = \left[\frac{S_{\text{silica}}}{S_{\text{micelle}}} \right]$$

The actual valence of a silica NP ($\boldsymbol{V}_{\mathbf{actual}}$, the number of surrounding, actually associated SV micelles) is an integrative consequence of the saturated valence $\boldsymbol{V}_{\mathbf{max}}$ and the feeding ratio of micelles to silica NPs $\boldsymbol{N}_{\mathbf{micelle}}/\boldsymbol{N}_{\mathbf{silica}}$. As a result, we can conclude that

$$\begin{cases} \dfrac{N_{\text{micelle}}}{N_{\text{silica}}} \geq V_{\text{max}} \Rightarrow V_{\text{actual}} = V_{\text{max}} \\ \dfrac{N_{\text{micelle}}}{N_{\text{silica}}} < V_{\text{max}} \Rightarrow V_{\text{actual}} = V_{\text{dynamic}} = 1 \sim V_{\text{max}} \end{cases}$$

where $\boldsymbol{N}_{\mathbf{micelle}}$ and $\boldsymbol{N}_{\mathbf{silica}}$ are the numbers of micelles and silica NPs added in the co-assembly system, respectively; $\boldsymbol{V}_{\mathbf{dynamic}}$ is the dynamic valence of a silica NP in the assembled dynamic structures (such as 1D colloidal chains and 2D/3D superstructures). When the feeding ratio $\boldsymbol{N}_{\mathbf{micelle}}/\boldsymbol{N}_{\mathbf{silica}}$ is greater than or equal to the saturated valence $\boldsymbol{V}_{\mathbf{max}}$, the number of micelles in the system can meet the silica NP demand of achieving a saturated valence and micelles-capped silica-NP-centered colloidal molecules will be generated. However, when $\boldsymbol{N}_{\mathbf{micelle}}/\boldsymbol{N}_{\mathbf{silica}}$ is less than $\boldsymbol{V}_{\mathbf{max}}$, the number of micelles is insufficient and various dynamic structures will be formed. For example, for $S_{2090}V_{380}$ micelles and 50-nm silica NPs at **A**:**B** ≈ **1**:**1**, micelles and silica NPs can connect with each other to form 1D colloidal chains (Supplementary Fig. 8) in which the valence of a silica NP remains to be 2 in order to achieve H-bonding maximization in spite of the insufficient number of micelles. Similar situation occurs for 2D/3D superstructures assembled by 75-nm/90-nm silica NPs (Supplementary Figs. 14 and 20).

For silica-NP-centered clusters, the surface of the silica NP was saturatedly covered by the P2VP coronas and the repulsion from the solvent-swollen P2VP coronas may cause the distance between the surrounding micelles. For SV-micelle-centered clusters, similarly, the electrostatic repulsion between the silica surface may render a specific distance between the surrounding NPs.

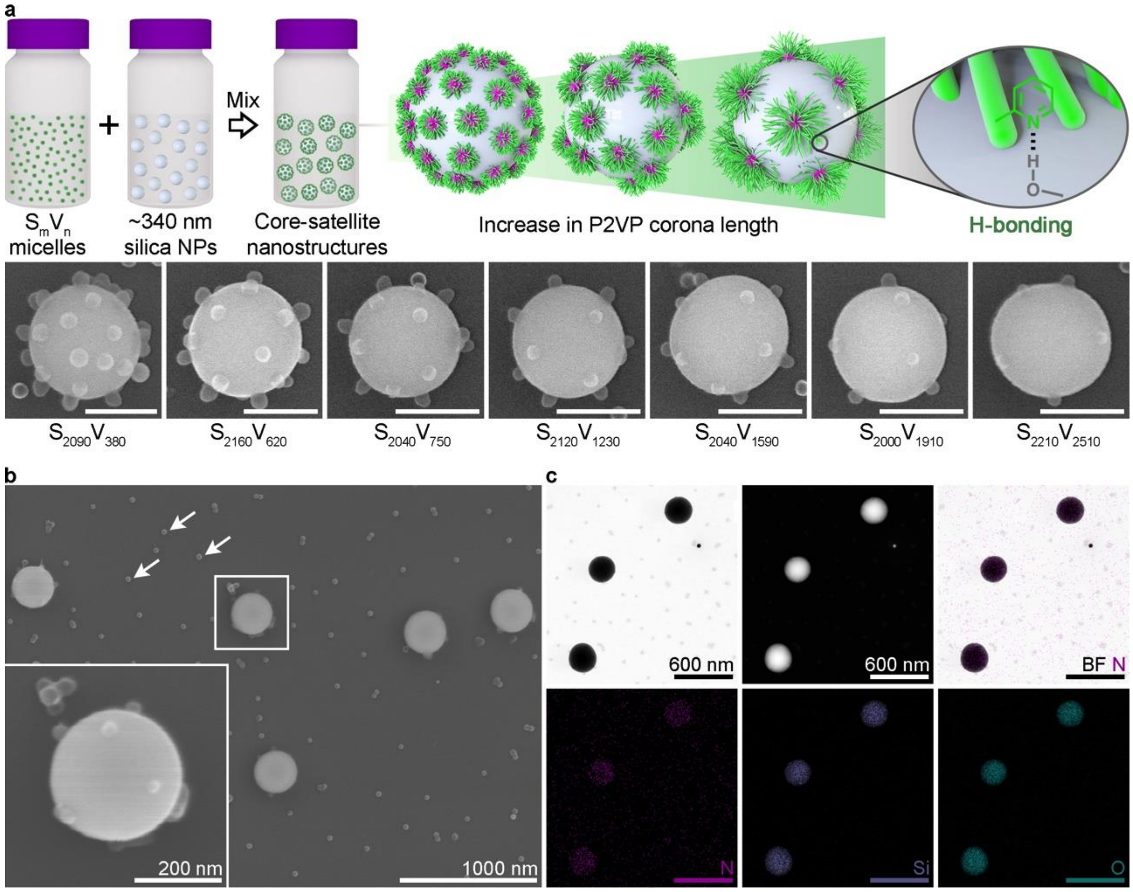

Supplementary Figure 27. Co-assembly of 340-nm silica NPs and SV micelles. The saturated valence of the central silica NP varies with the P2VP corona length of SV micelles. (**a**) Schematic illustration and representative SEM images of micelles-surrounded core-satellite nanostructures assembled by 340-nm silica NPs and $S_{2090}V_{380}$, $S_{2160}V_{620}$, $S_{2040}V_{750}$, $S_{2120}V_{1230}$, $S_{2040}V_{1590}$, $S_{2000}V_{1910}$, $S_{2210}V_{2510}$ micelles (from left to right), respectively. An increase in P2VP corona length results in a decrease in the number of surface-attached SV micelles. Scale bars = 200 nm. (**b**) Wide-field SEM image of core-satellite nanostructures assembled by 340-nm silica NPs and $S_{2210}V_{2510}$ micelles. The white arrows denote the excessive, unassociated $S_{2210}V_{2510}$ micelles. The presence of excess unbound $S_{2210}V_{2510}$ micelles indicates the saturation of the central silica NP surface due to the coverage of P2VP coronas. (**c**) Representative BF-STEM, HAADF-STEM and corresponding EDS elemental mapping images of core-satellite nanostructures assembled by 340-nm silica NPs and $S_{2210}V_{2510}$ micelles. For the EDS mapping images, the purple features represent N existing in the P2VP coronas of $S_{2210}V_{2510}$ micelles.

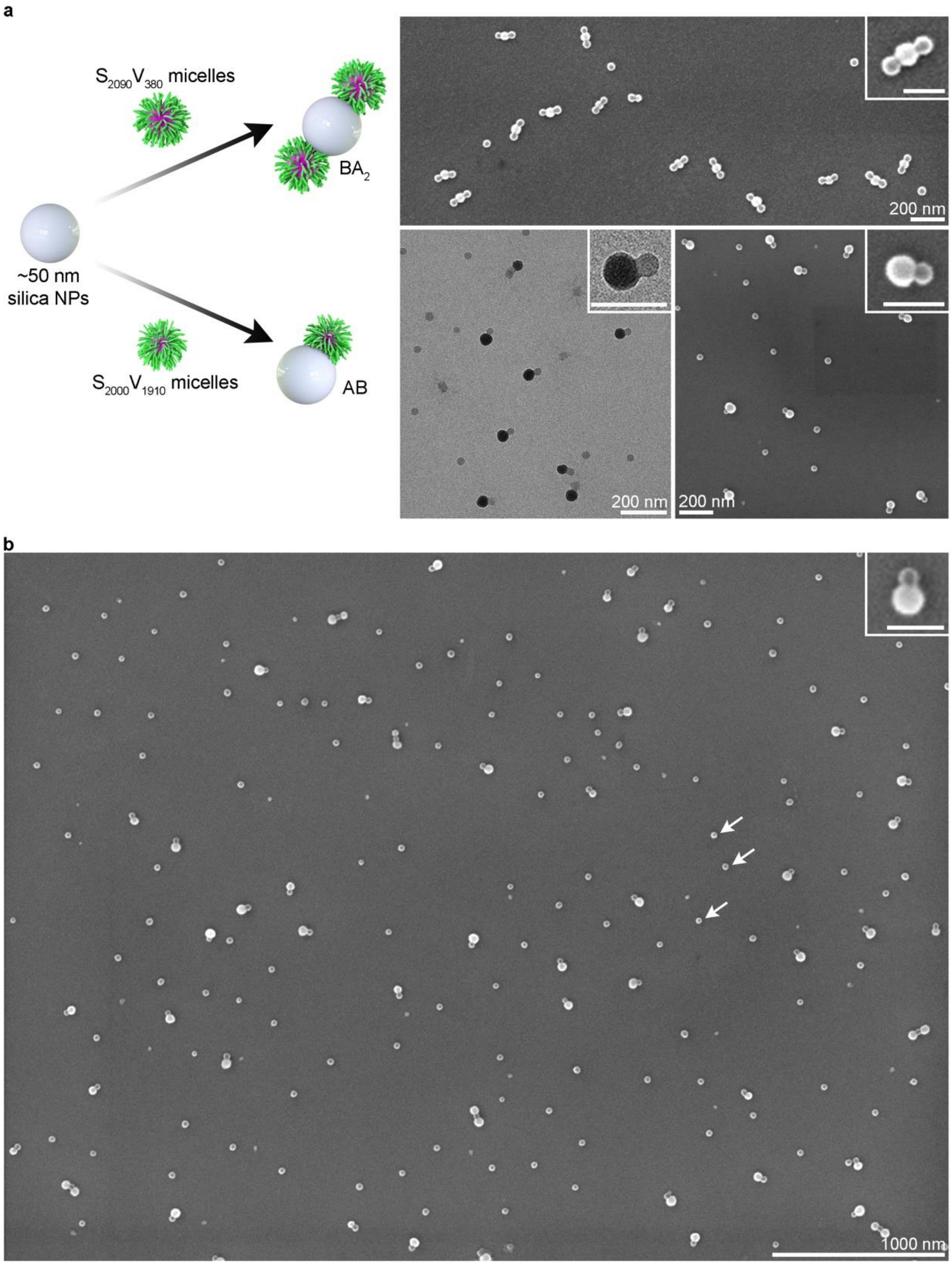

Supplementary Figure 28. Co-assembly of 50-nm silica NPs and $S_{2000}V_{1910}$ micelles. (**a**) Schematic illustration and representative TEM and SEM images of AB dimers assembled by 50-nm silica NPs and $S_{2000}V_{1910}$ micelles. (**b**) Wide-field SEM image of the assembled AB dimers. The white arrows denote the excessive, unassociated $S_{2000}V_{1910}$ micelles. The presence of excess unbound $S_{2000}V_{1910}$ micelles indicates that the 50-nm silica NP is saturated. Inset scale bars = 100 nm.

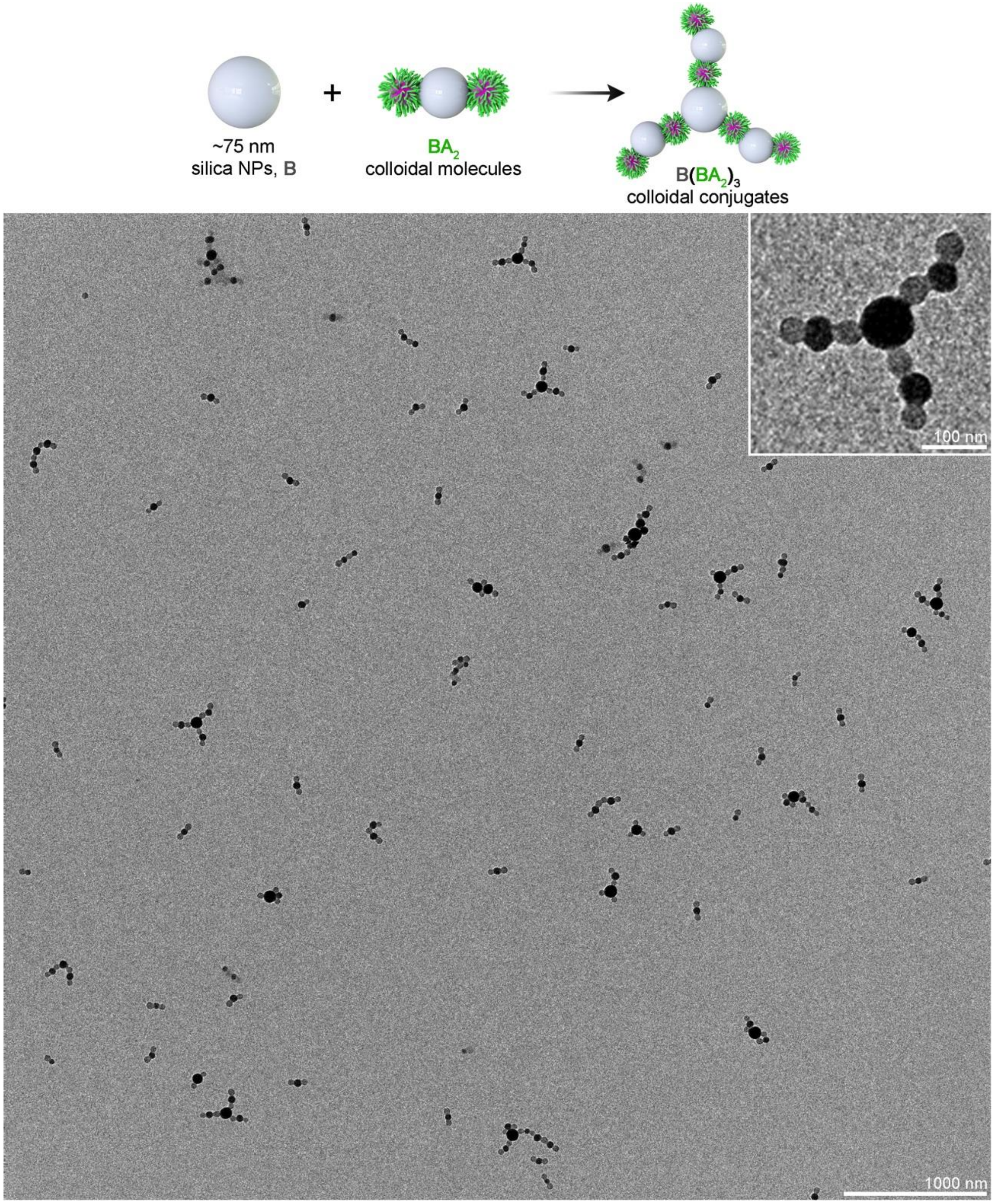

Supplementary Figure 29. Wide-field TEM image of $B(BA_2)_3$ colloidal conjugates. $B(BA_2)_3$ colloidal conjugates were assembled by 75-nm silica NPs and BA_2 colloidal molecules (assembled by $S_{2090}V_{380}$ micelles and 50-nm silica NPs).

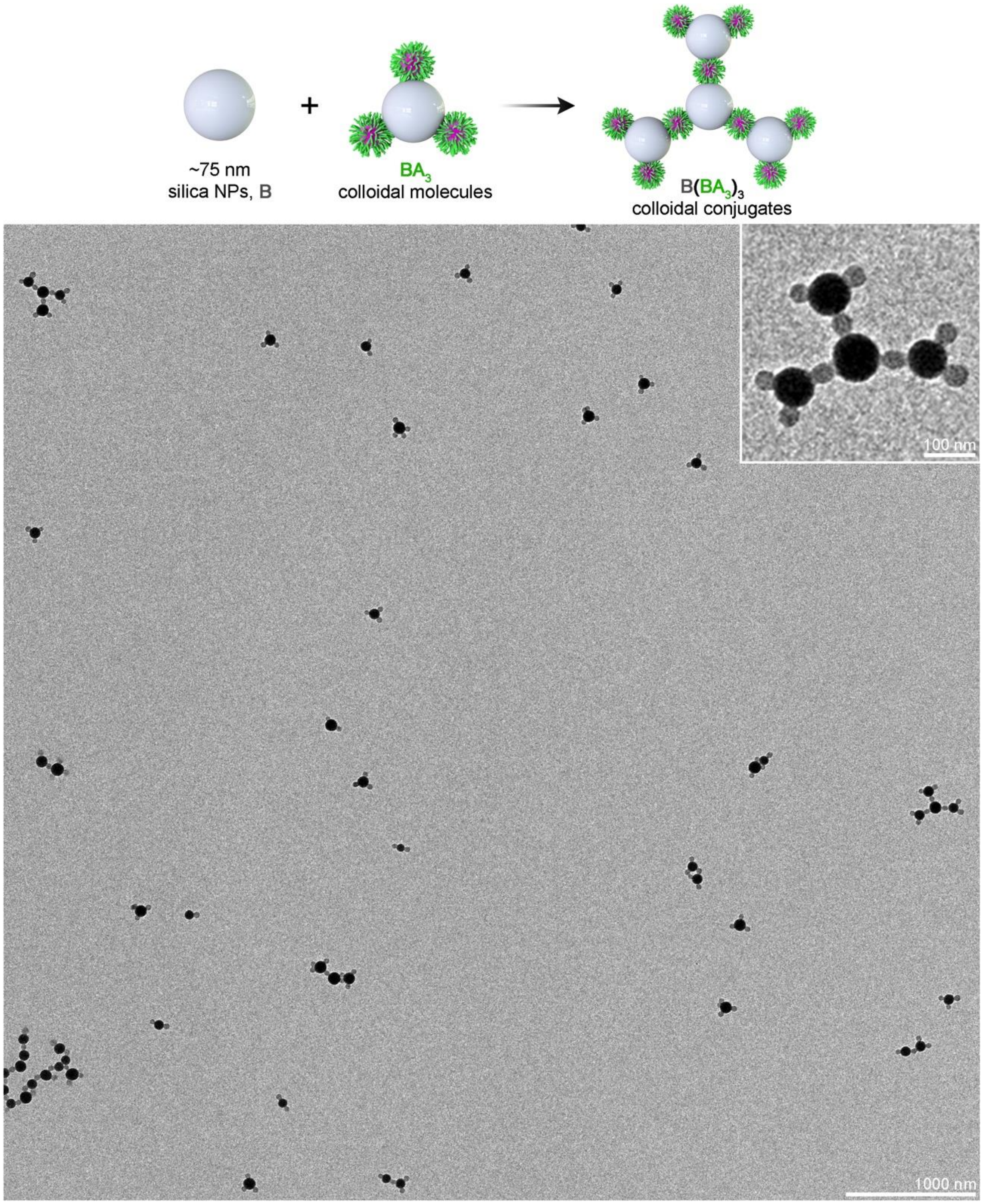

Supplementary Figure 30. Wide-field TEM image of B(BA$_3$)$_3$ colloidal conjugates. B(BA$_3$)$_3$ colloidal conjugates were assembled by 75-nm silica NPs and BA$_3$ colloidal molecules (assembled by $S_{2090}V_{380}$ micelles and 75-nm silica NPs).

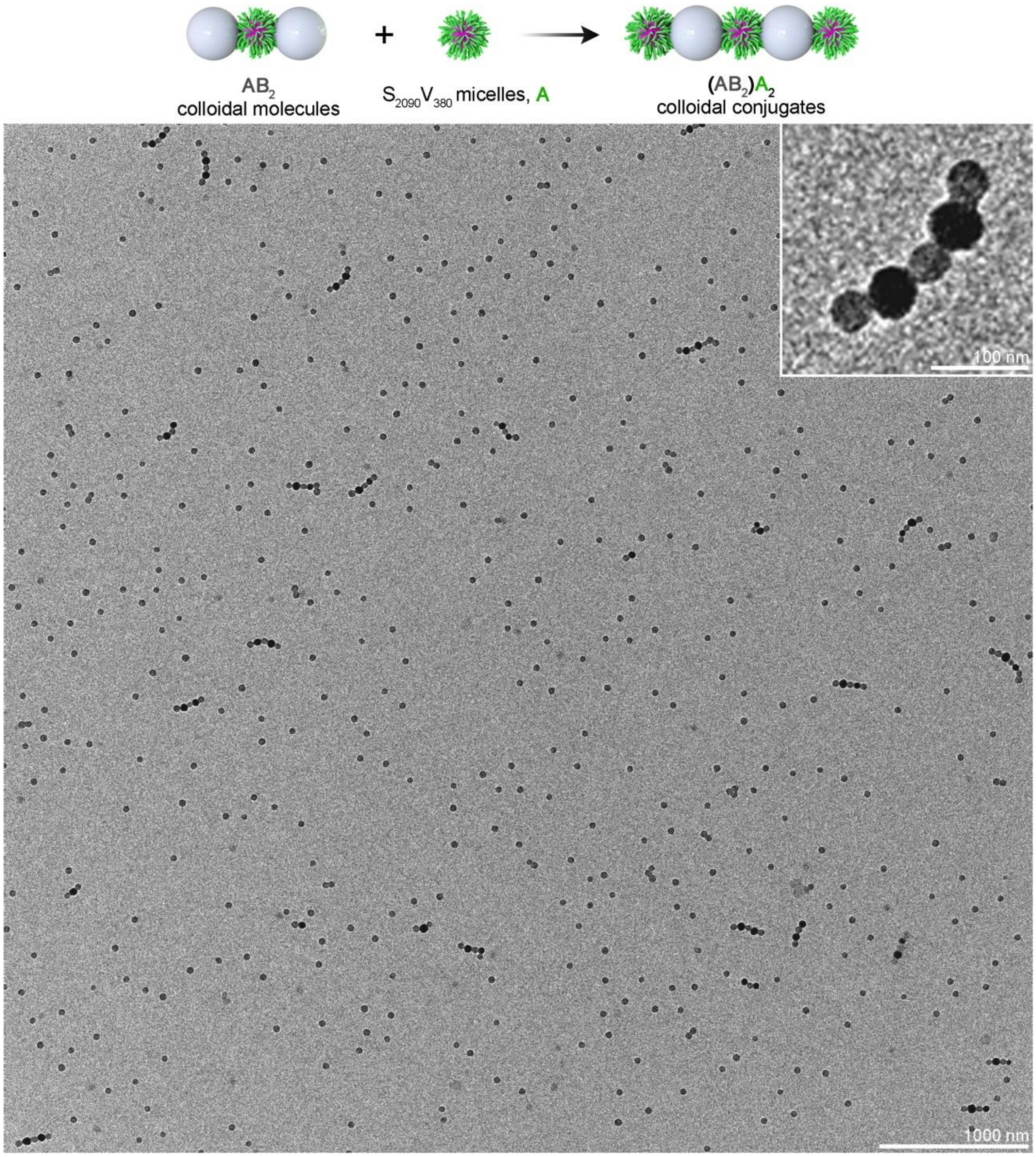

Supplementary Figure 31. Wide-field TEM image of $(AB_2)A_2$ colloidal conjugates. $(AB_2)A_2$ colloidal conjugates were assembled by AB_2 colloidal molecules (assembled by $S_{2090}V_{380}$ micelles and 50-nm silica NPs) and $S_{2090}V_{380}$ micelles.

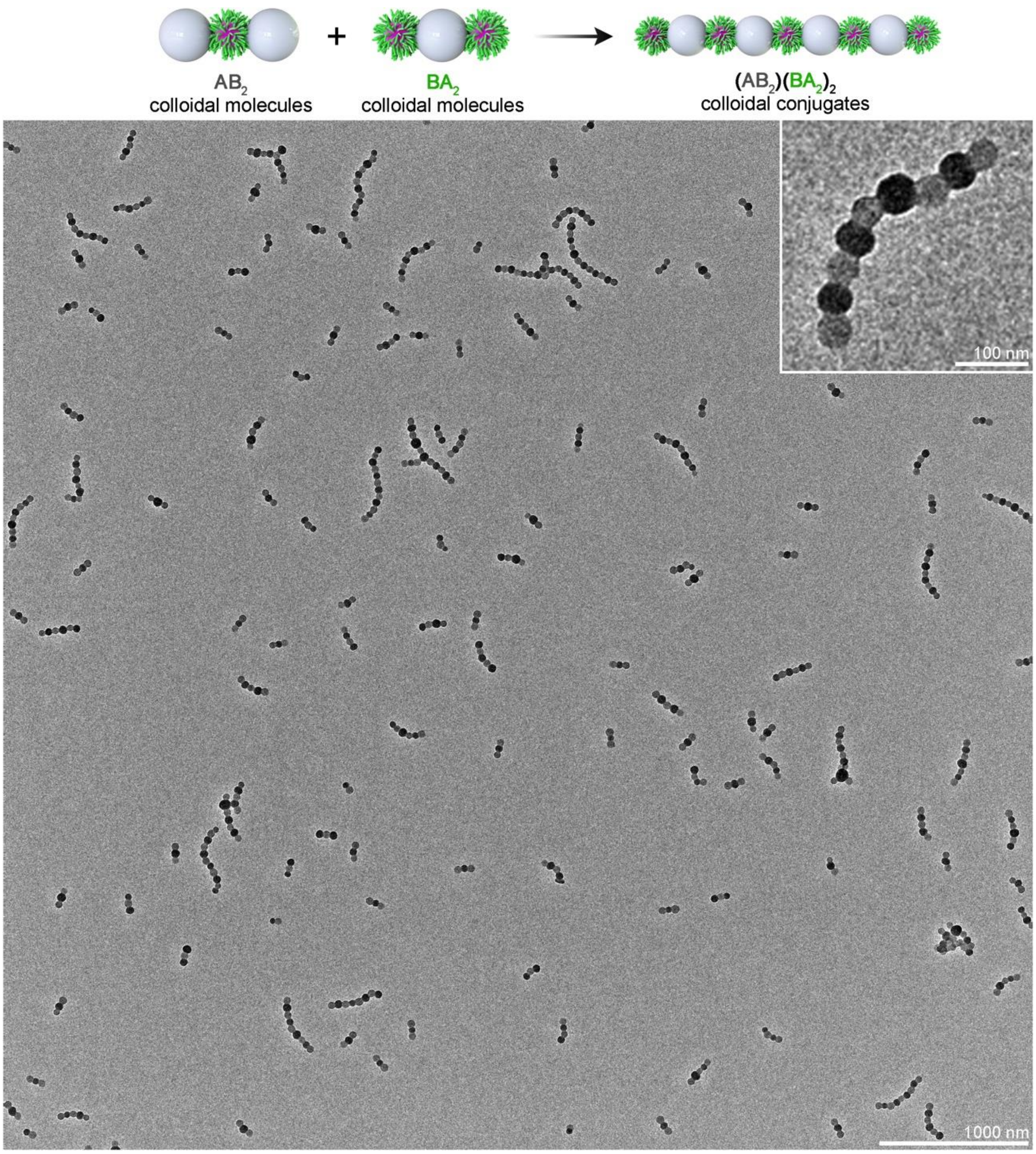

Supplementary Figure 32. Wide-field TEM image of $(AB_2)(BA_2)_2$ colloidal conjugates. $(AB_2)(BA_2)_2$ colloidal conjugates were assembled by AB_2 colloidal molecules (assembled by $S_{2090}V_{380}$ micelles and 50-nm silica NPs) and BA_2 colloidal molecules (assembled by $S_{2090}V_{380}$ micelles and 50-nm silica NPs).

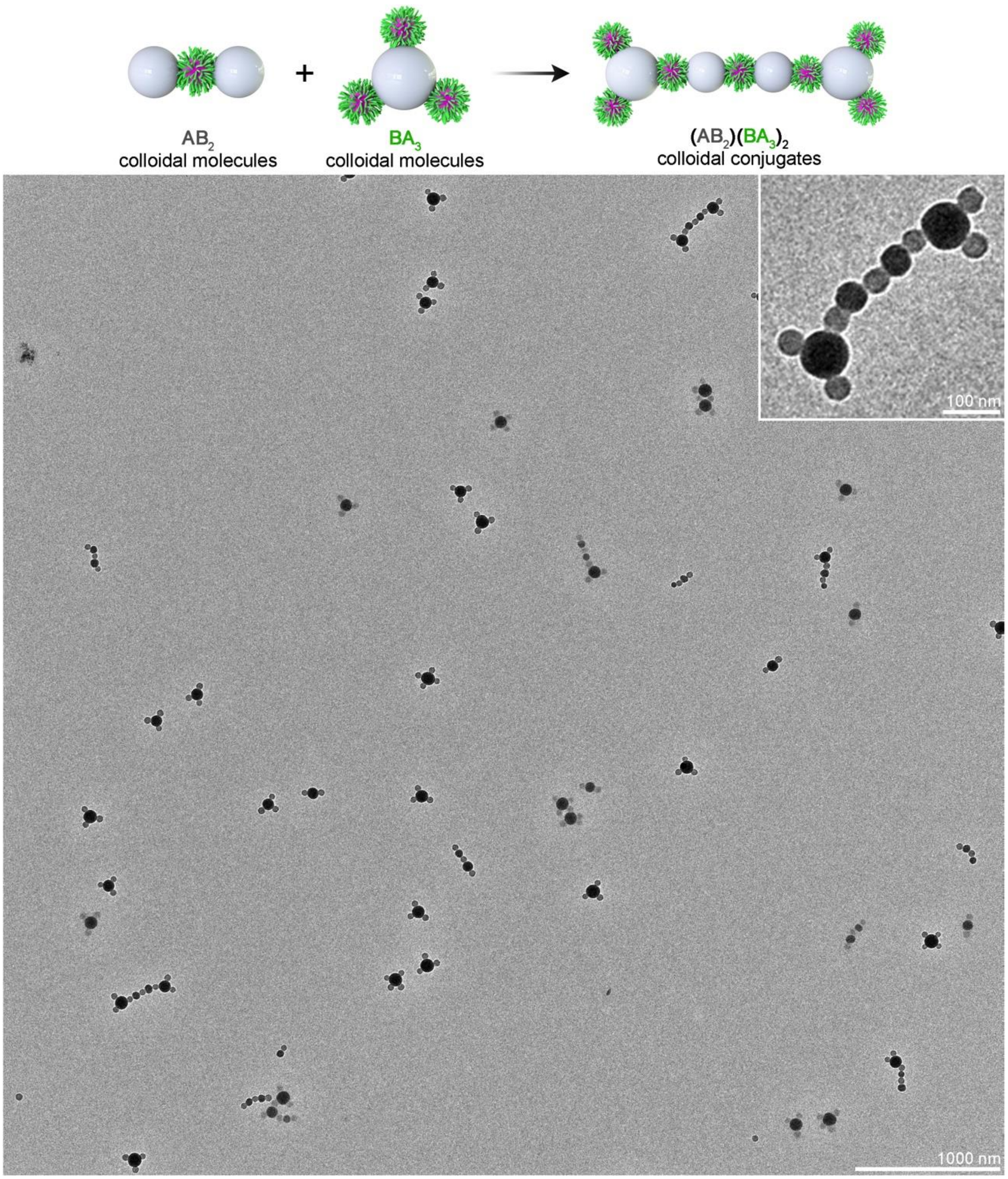

Supplementary Figure 33. Wide-field TEM image of $(AB_2)(BA_3)_2$ colloidal conjugates. $(AB_2)(BA_3)_2$ colloidal conjugates were assembled by AB_2 colloidal molecules (assembled by $S_{2090}V_{380}$ micelles and 50-nm silica NPs) and BA_3 colloidal molecules (assembled by $S_{2090}V_{380}$ micelles and 75-nm silica NPs).

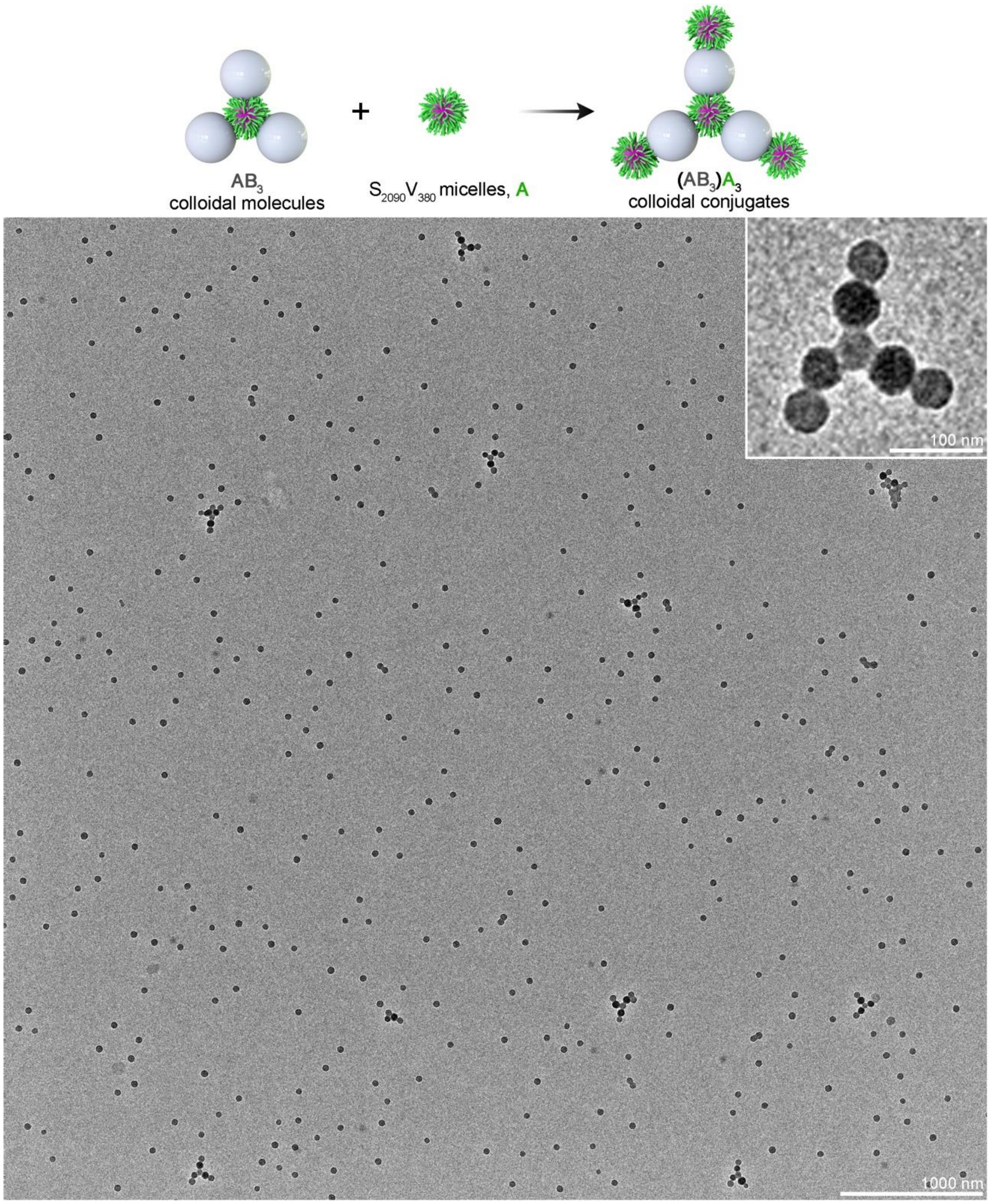

Supplementary Figure 34. Wide-field TEM image of $(AB_3)A_3$ colloidal conjugates. $(AB_3)A_3$ colloidal conjugates were assembled by AB_3 colloidal molecules (assembled by $S_{2090}V_{380}$ micelles and 50-nm silica NPs) and $S_{2090}V_{380}$ micelles.

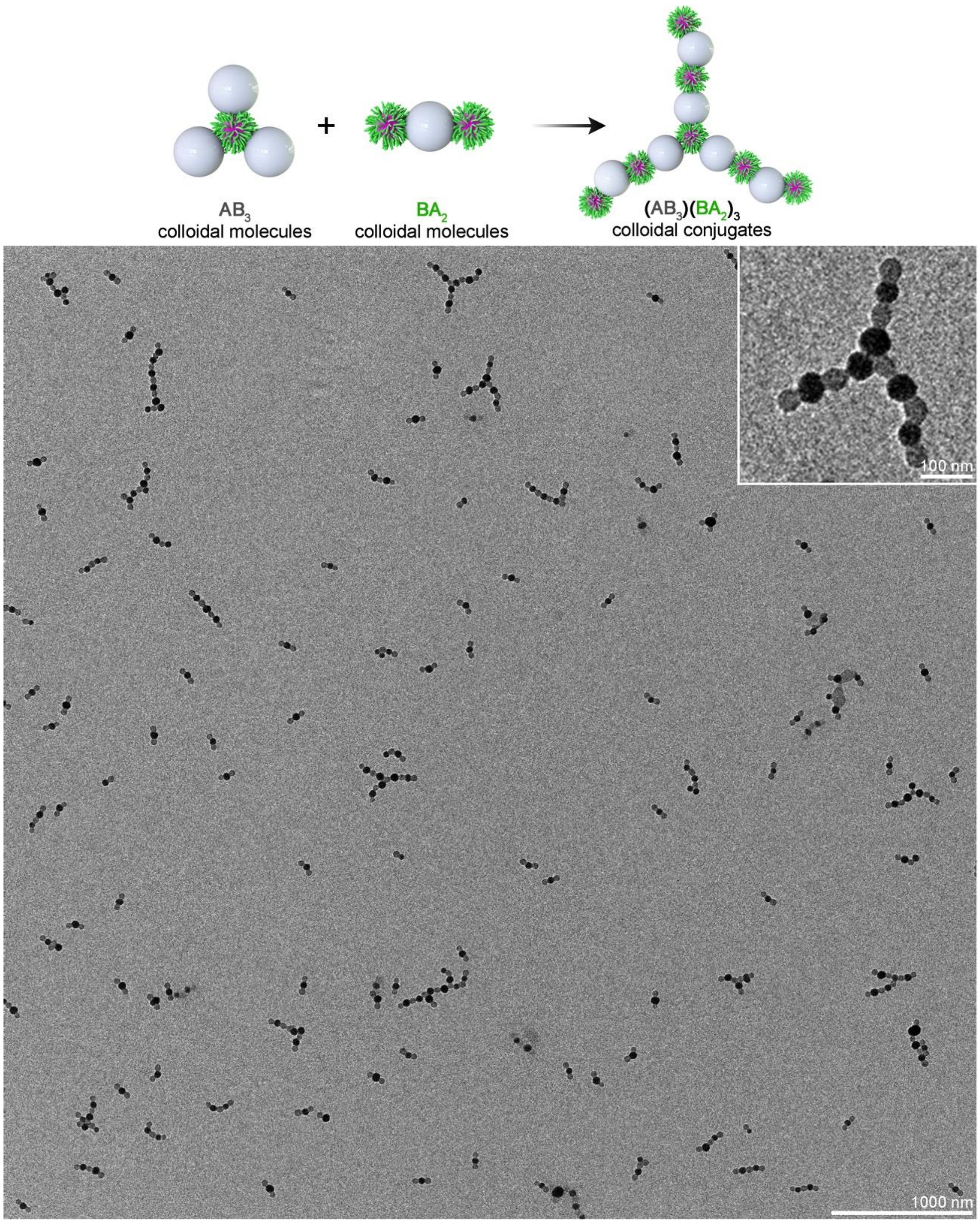

Supplementary Figure 35. Wide-field TEM image of $(AB_3)(BA_2)_3$ colloidal conjugates. $(AB_3)(BA_2)_3$ colloidal conjugates were assembled by AB_3 colloidal molecules (assembled by $S_{2090}V_{380}$ micelles and 50-nm silica NPs) and BA_2 colloidal molecules (assembled by $S_{2090}V_{380}$ micelles and 50-nm silica NPs).

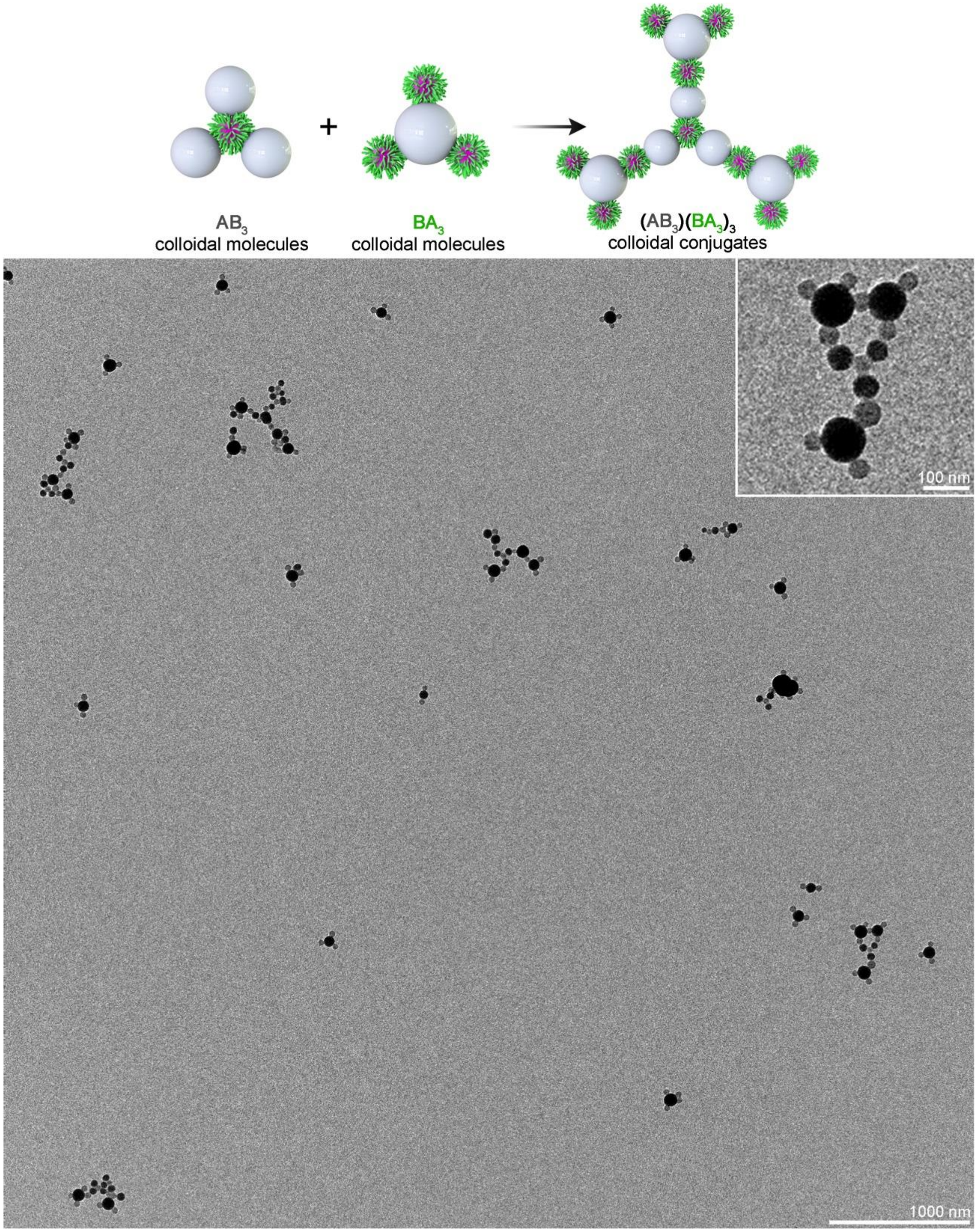

Supplementary Figure 36. Wide-field TEM image of $(AB_3)(BA_3)_3$ colloidal conjugates. $(AB_3)(BA_3)_3$ colloidal conjugates were assembled by AB_3 colloidal molecules (assembled by $S_{2090}V_{380}$ micelles and 50-nm silica NPs) and BA_3 colloidal molecules (assembled by $S_{2090}V_{380}$ micelles and 75-nm silica NPs).

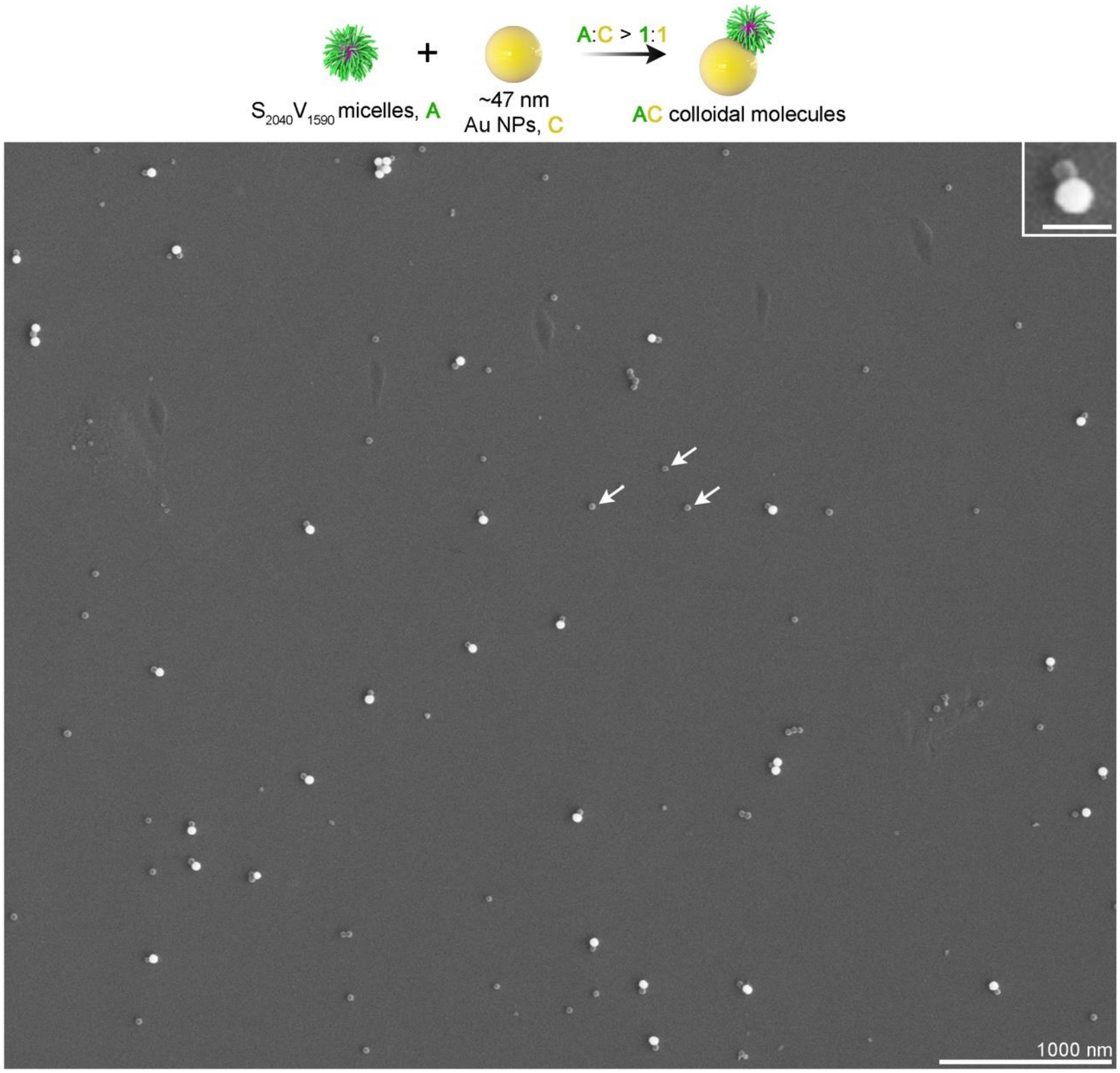

Supplementary Figure 37. Wide-field SEM image of AC dimers assembled by $S_{2040}V_{1590}$ micelles and 47-nm Au NPs. The white arrows denote the excessive, unassociated $S_{2040}V_{1590}$ micelles. Inset scale bars = 100 nm.

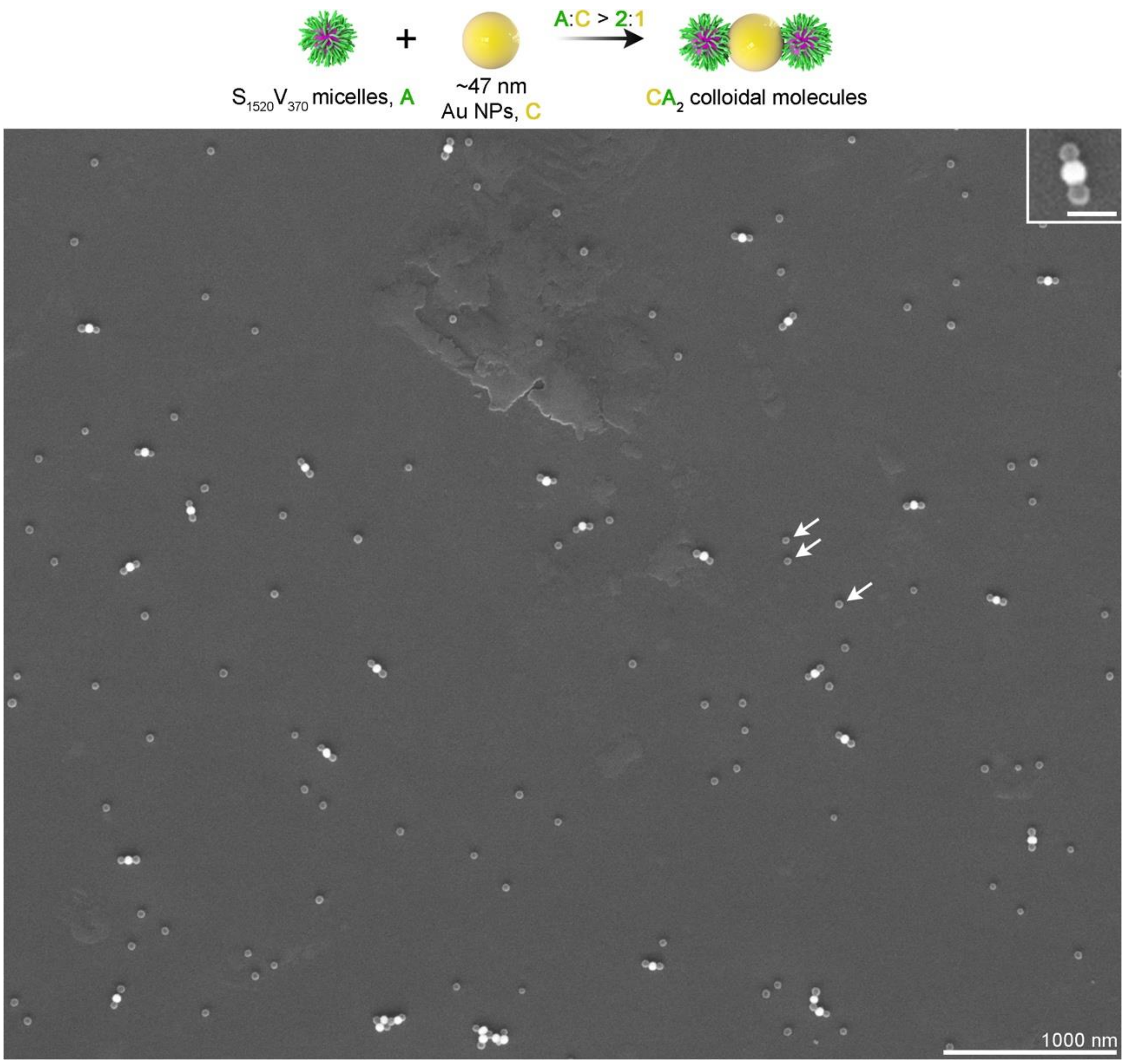

Supplementary Figure 38. Wide-field SEM image of CA_2 trimers assembled by $S_{1520}V_{370}$ micelles and 47-nm Au NPs. The white arrows denote the excessive, unassociated $S_{1520}V_{370}$ micelles. Inset scale bars = 100 nm.

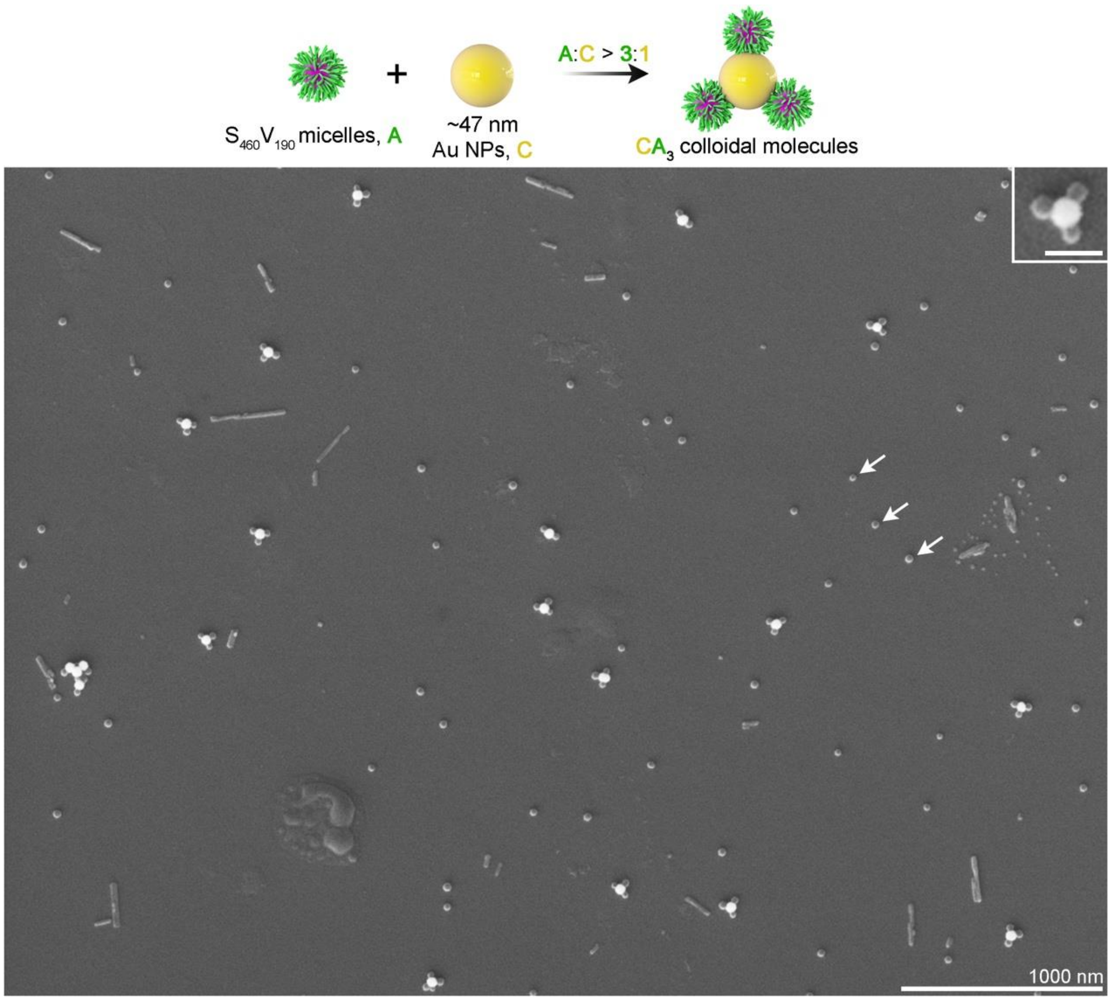

Supplementary Figure 39. Wide-field SEM image of CA_3 tetramers assembled by $S_{460}V_{190}$ micelles and 47-nm Au NPs. The white arrows denote the excessive, unassociated $S_{460}V_{190}$ micelles. Inset scale bars = 100 nm.

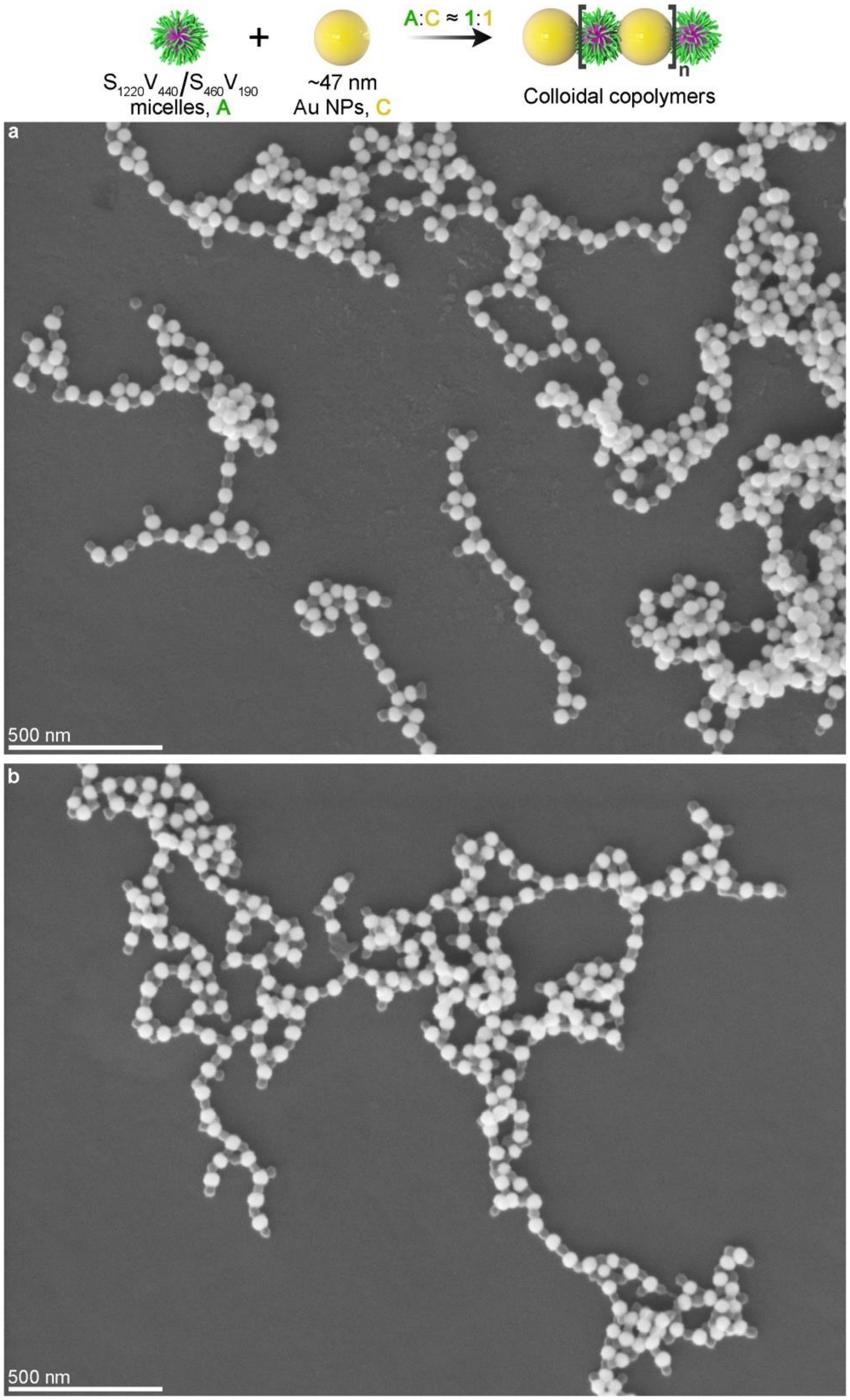

Supplementary Figure 40. 1D alternating colloidal copolymers assembled by SV micelles (a) and 47-nm Au NPs (C) in A:C ≈ 1:1. (**A**) Wide-field SEM image of the resultant alternating colloidal copolymers formed by $S_{1220}V_{440}$ micelles and 47-nm Au NPs. (**b**) Wide-field SEM image of the resultant alternating colloidal copolymers formed by $S_{460}V_{190}$ micelles and 47-nm Au NPs.

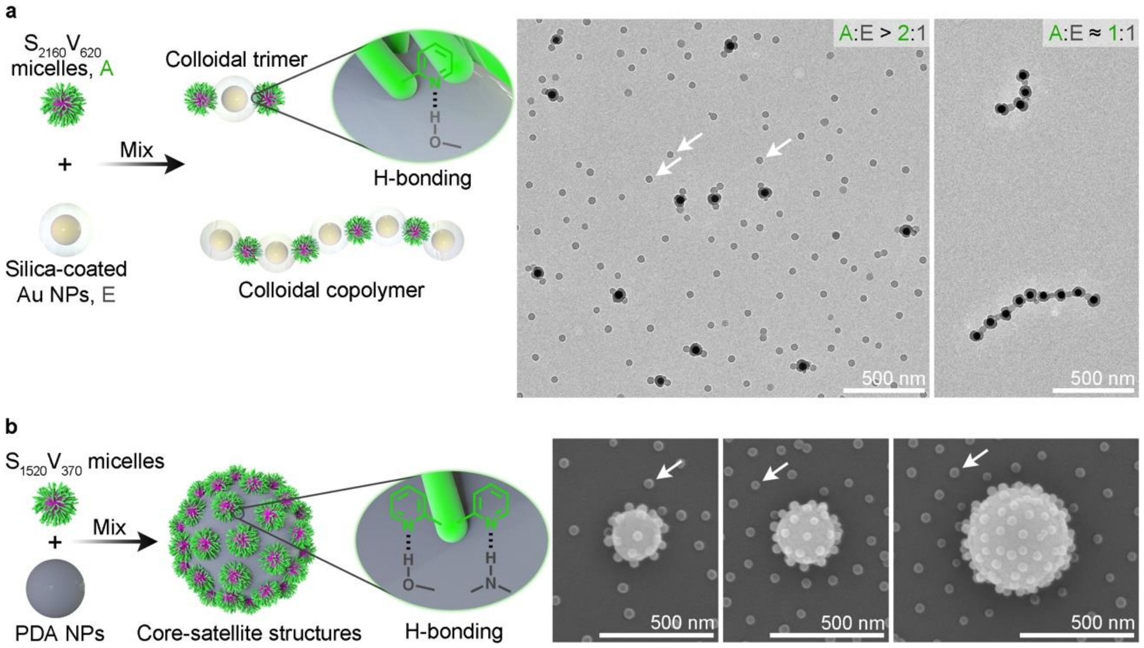

Supplementary Figure 41. H-bonding driven co-assembly of SV micelles and other hard NPs. (**a**) Schematic illustration and representative TEM images of micelles-capped colloidal trimers (left) and alternating colloidal copolymers (right) assembled by $S_{2160}V_{620}$ micelles and 77-nm core/shell structured silica-coated Au NPs in different feeding ratios. The white arrows denote the excessive, unassociated $S_{2160}V_{620}$ micelles. (**b**) Schematic illustration and representative SEM images of the micelles-surrounded core-satellite nanostructures assembled by PDA NPs (number-average diameter = 210 nm, 250 nm, and 440 nm, respectively, from left to right) and $S_{1520}V_{370}$ micelles. The white arrows denote the excessive, unassociated $S_{1520}V_{370}$ micelles.

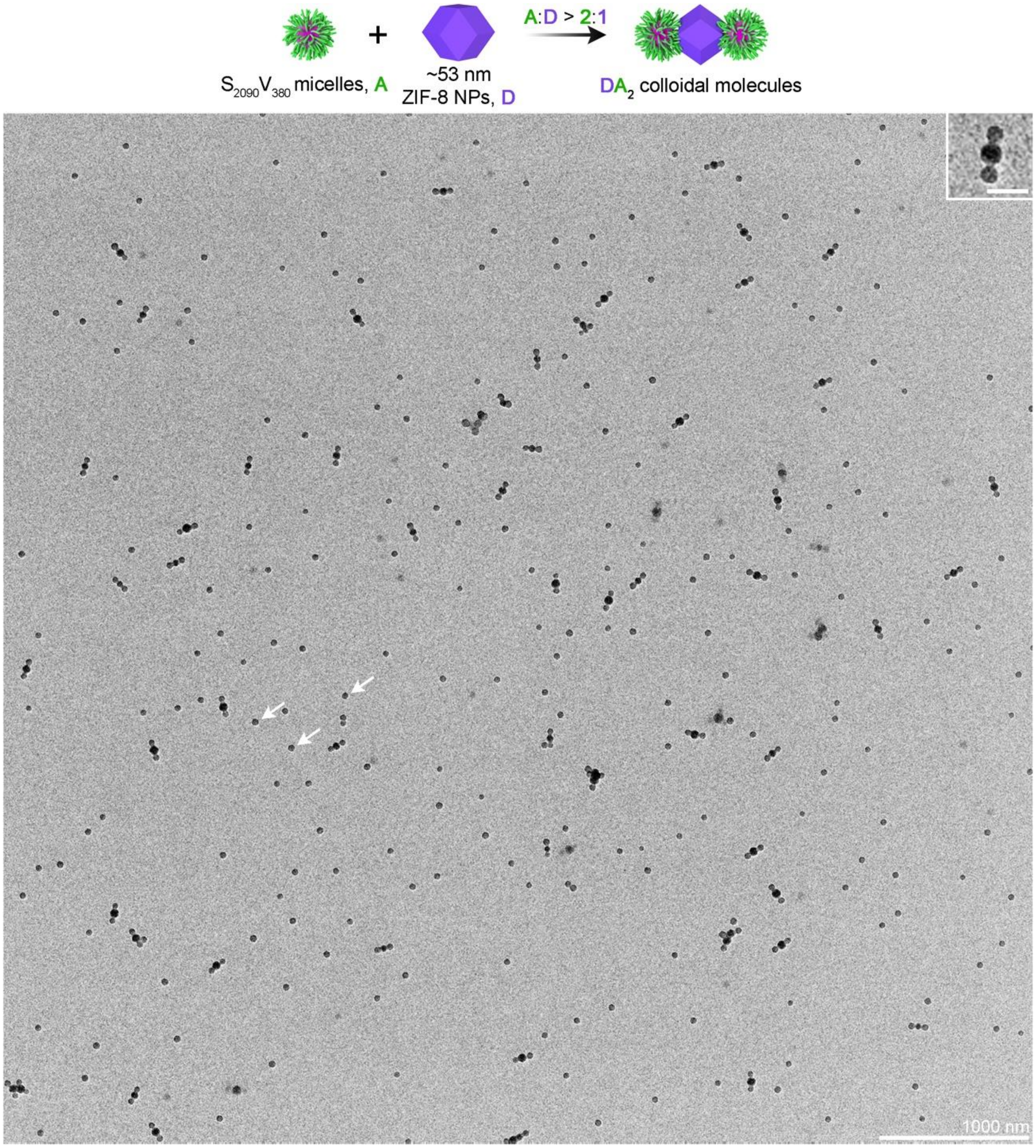

Supplementary Figure 42. Wide-field TEM image of DA_2 trimers assembled by $S_{2090}V_{380}$ micelles and 53-nm ZIF-8 NPs. The white arrows denote the excessive, unassociated $S_{2090}V_{380}$ micelles. Inset scale bars = 100 nm.

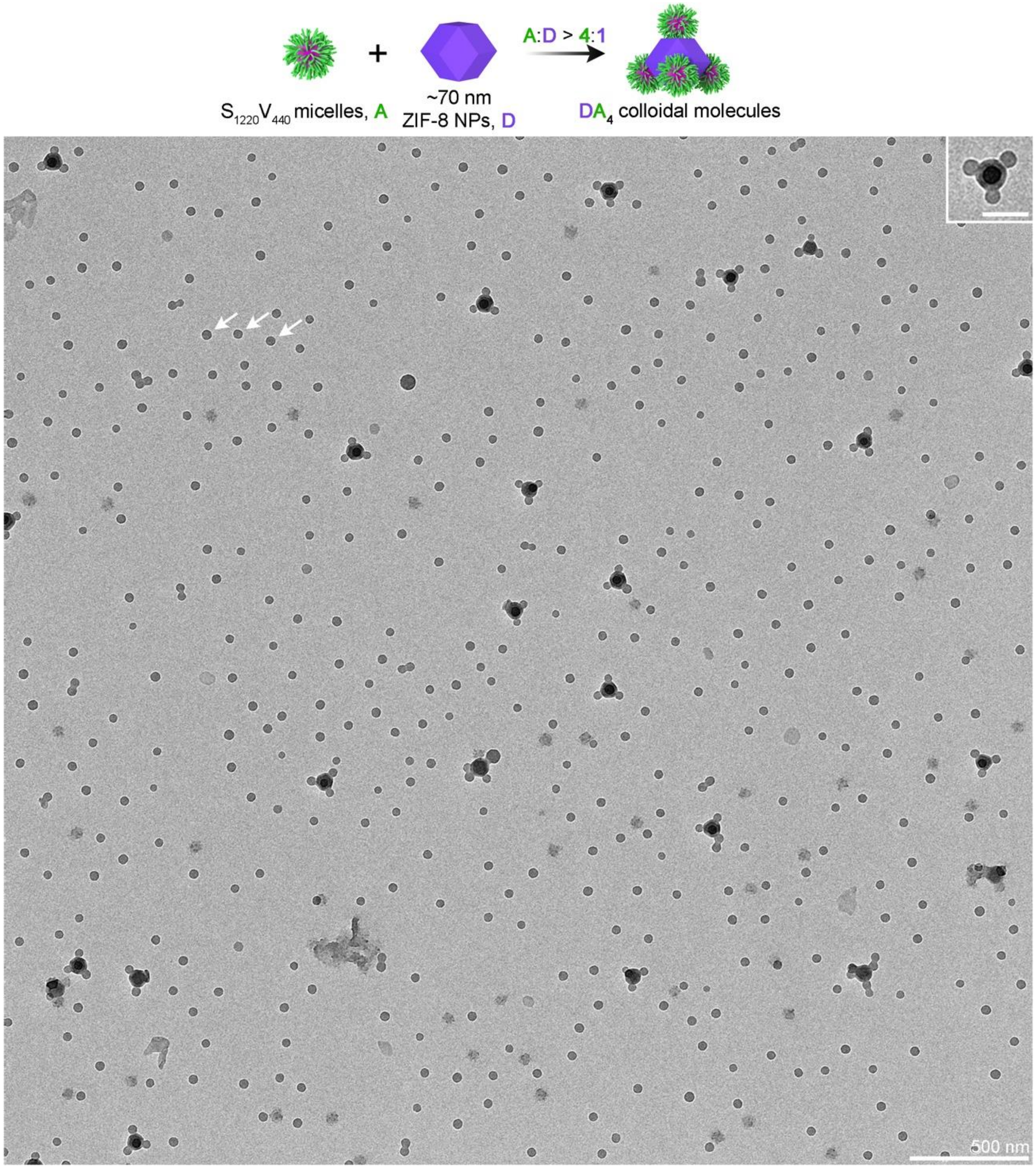

Supplementary Figure 43. Wide-field TEM image of DA$_4$ pentamers assembled by S$_{1220}$V$_{440}$ micelles and 70-nm ZIF-8 NPs. The white arrows denote the excessive, unassociated S$_{1220}$V$_{440}$ micelles. Inset scale bars = 100 nm.

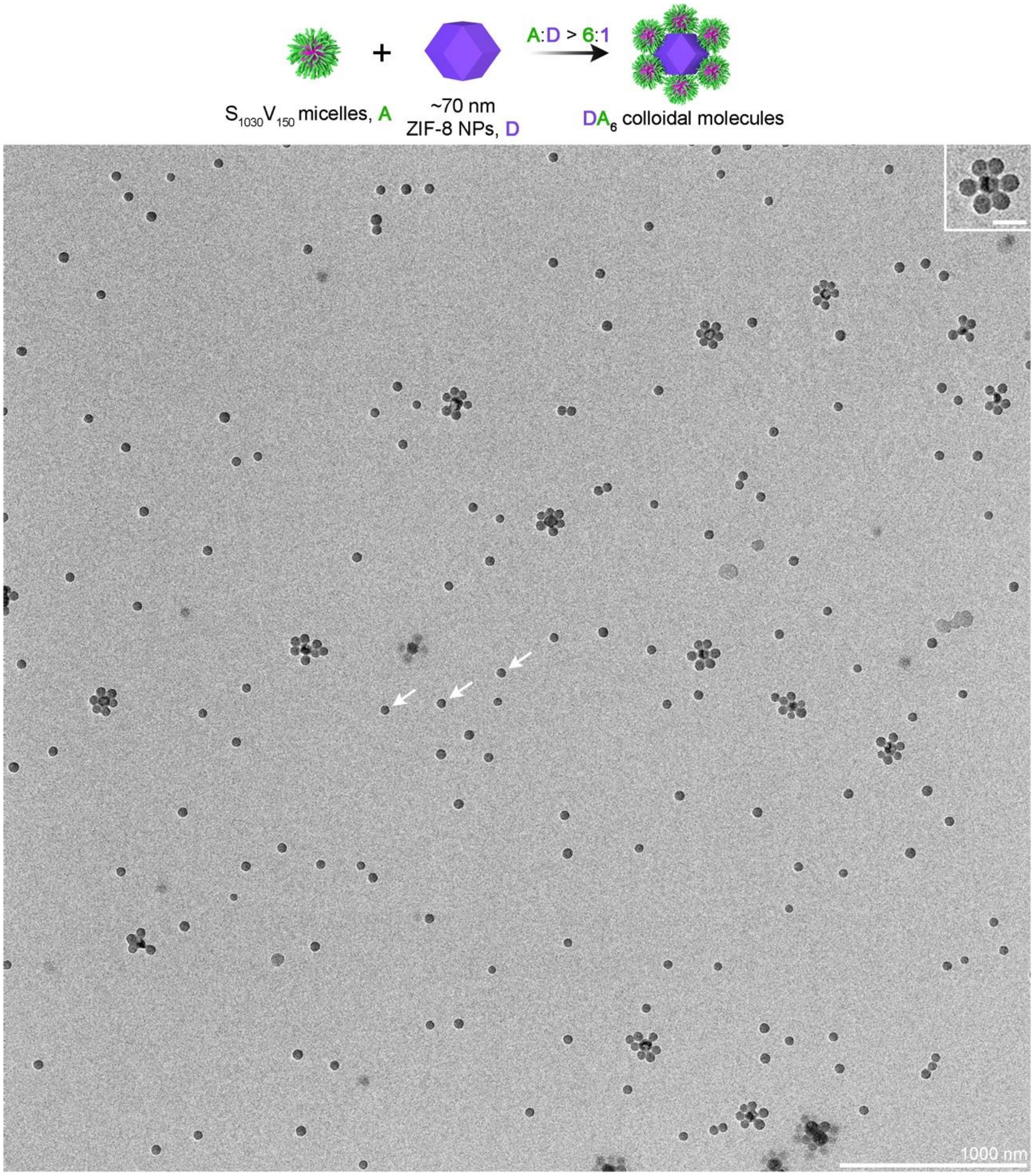

Supplementary Figure 44. Wide-field TEM image of DA_6 heptamers assembled by $S_{1030}V_{150}$ micelles and 70-nm ZIF-8 NPs. The white arrows denote the excessive, unassociated $S_{1030}V_{150}$ micelles. Inset scale bars = 100 nm.

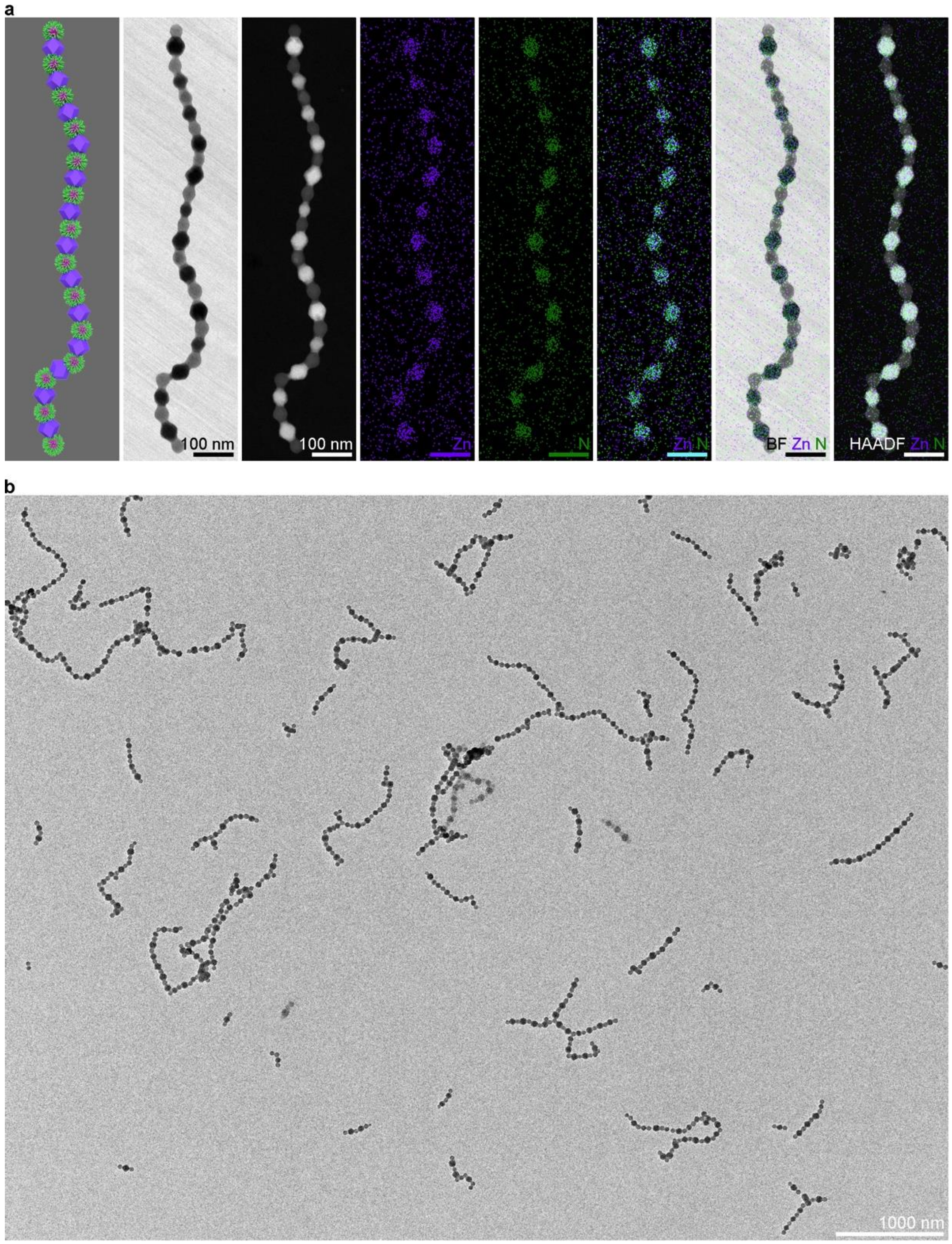

Supplementary Figure 45. 1D alternating colloidal copolymers assembled by $S_{2090}V_{380}$ micelles (A) and 53-nm ZIF-8 NPs (D) in A:D ≈ 1:1. (**a**) Schematic illustration and representative BF-STEM, HAADF-STEM and corresponding EDS elemental mapping images (Zn is colored in blue and N is colored in green) of the assembled alternating colloidal copolymers. (**b**) Wide-field TEM image of the assembled alternating colloidal copolymers.

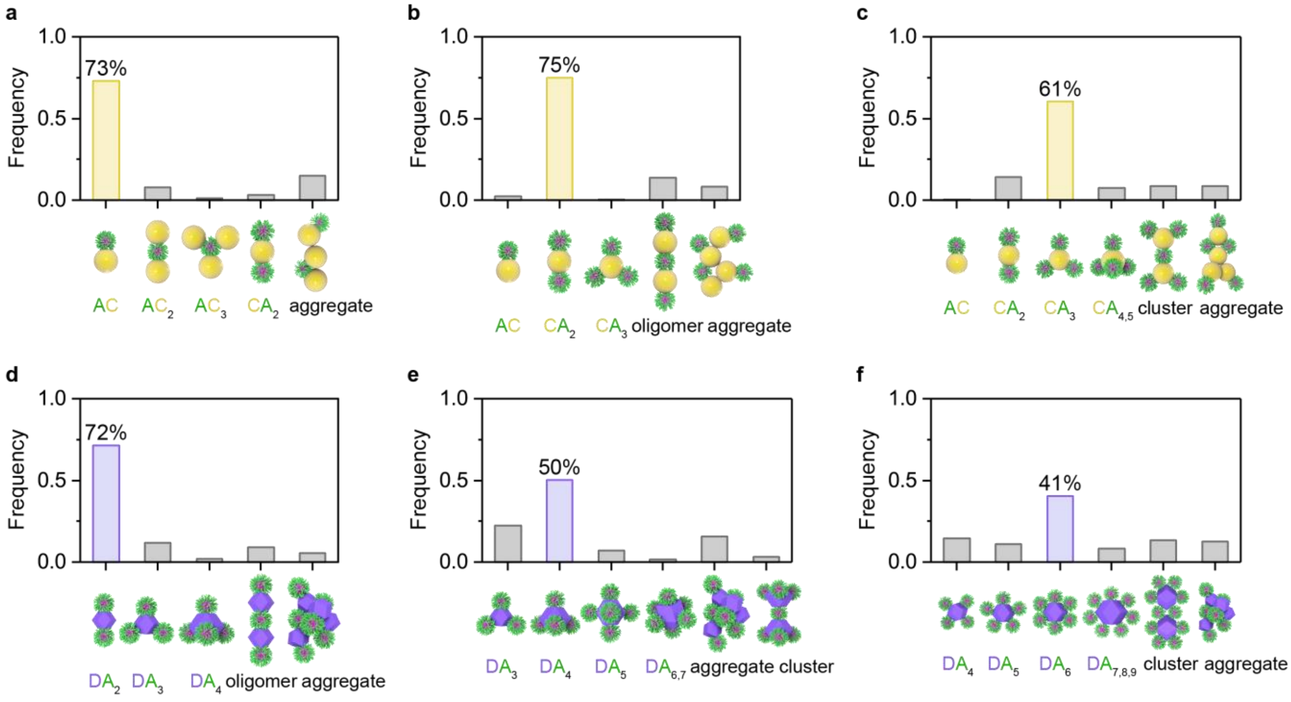

Supplementary Figure 46. Statistical analysis of the assembled colloidal architectures. (**a**) AC dimers assembled by $S_{2040}V_{1590}$ micelles and 47-nm Au NPs (corresponding to Supplementary Fig. 37). (**b**) CA_2 trimers assembled by $S_{1520}V_{370}$ micelles and 47-nm Au NPs (corresponding to Supplementary Fig. 38). (**c**) CA_3 tetramers assembled by $S_{460}V_{190}$ micelles and 47-nm Au NPs (corresponding to Supplementary Fig. 39). (**d**) DA_2 trimers assembled by $S_{2090}V_{380}$ micelles and 53-nm ZIF-8 NPs (corresponding to Supplementary Fig. 42). (**e**) DA_4 pentamers assembled by $S_{1220}V_{440}$ micelles and 70-nm ZIF-8 NPs (corresponding to Supplementary Fig. 43). (**f**) DA_6 heptamers assembled by $S_{1030}V_{150}$ micelles and 70-nm ZIF-8 NPs (corresponding to Supplementary Fig. 44).

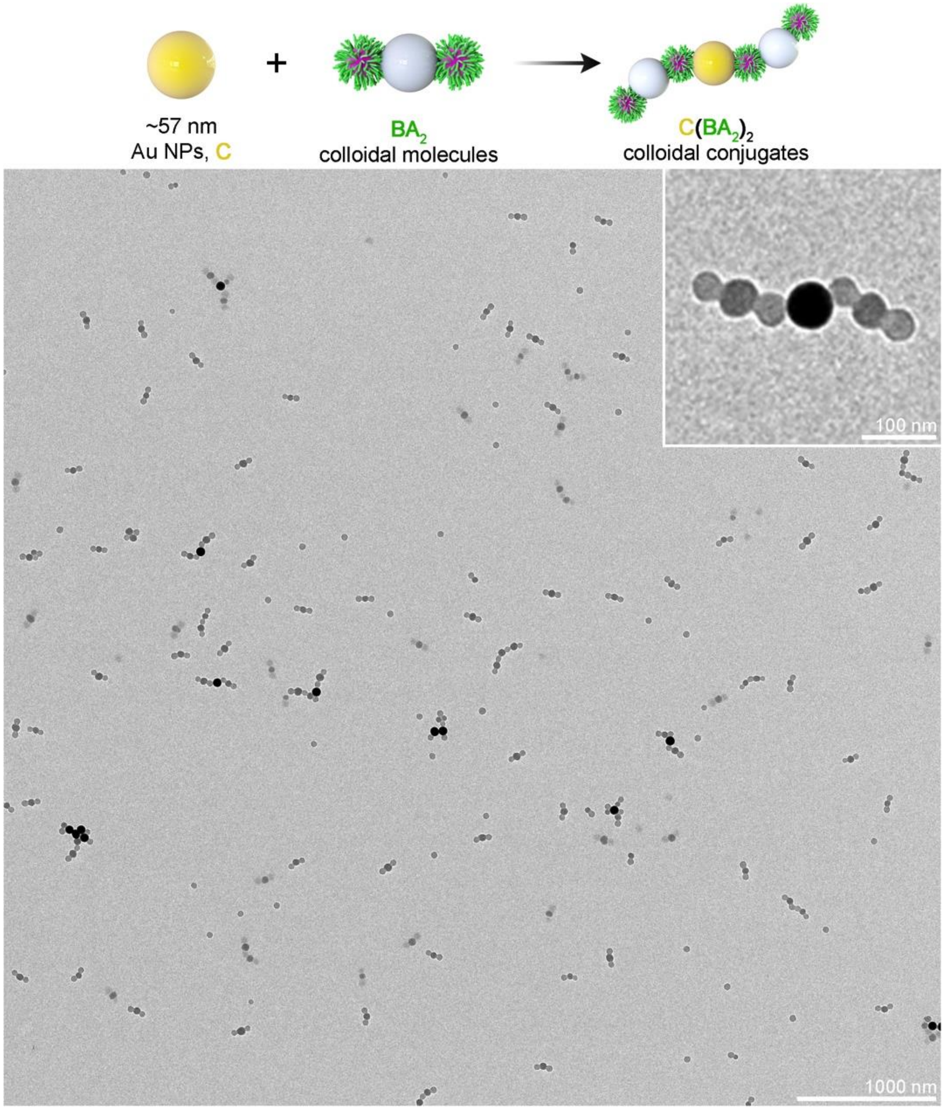

Supplementary Figure 47. Wide-field TEM image of $C(BA_2)_2$ colloidal conjugates. $C(BA_2)_2$ colloidal conjugates were assembled by 57-nm Au NPs and BA_2 colloidal molecules (assembled by $S_{2090}V_{380}$ micelles and 50-nm silica NPs).

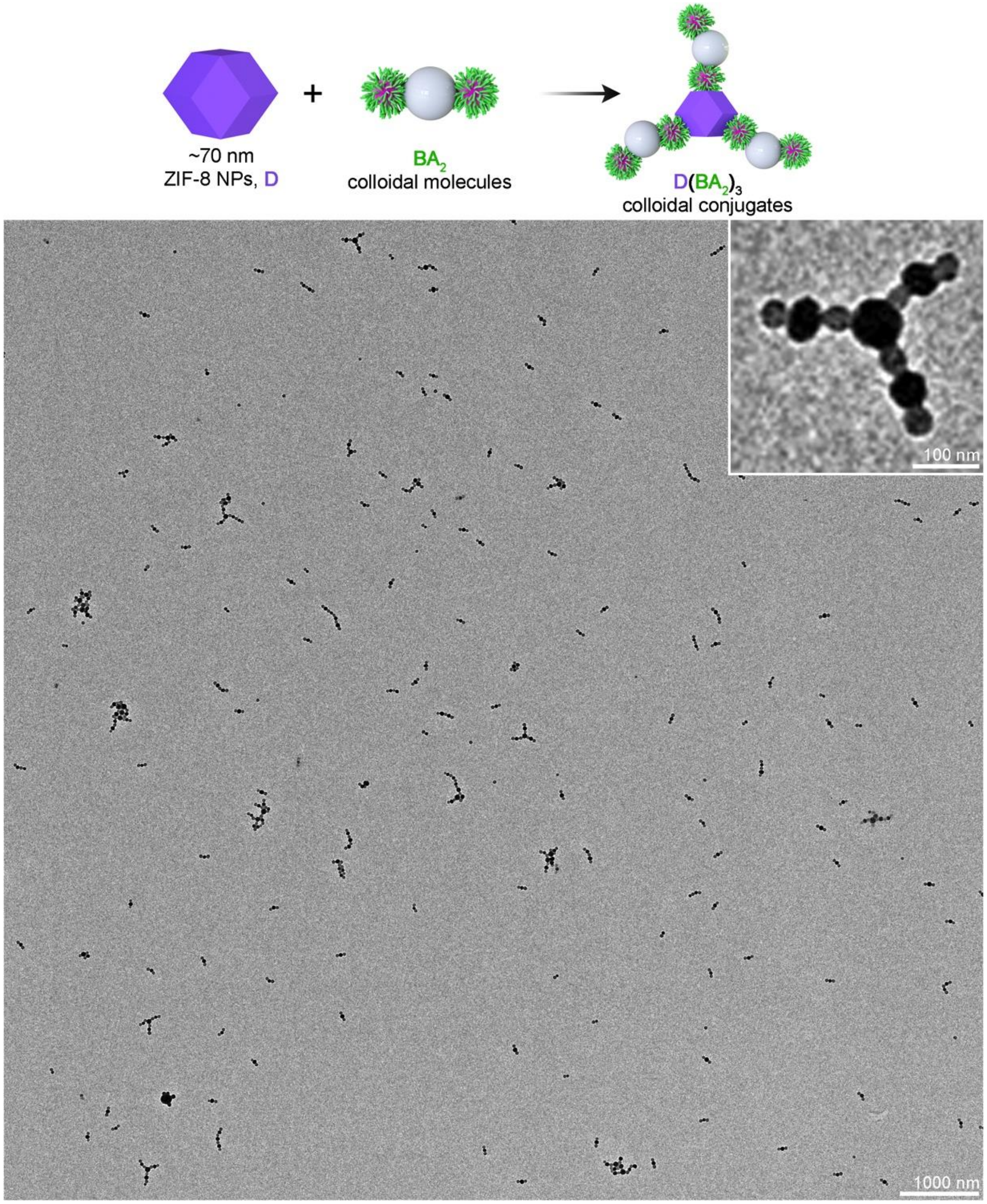

Supplementary Figure 48. Wide-field TEM image of D(BA$_2$)$_3$ colloidal conjugates. D(BA$_2$)$_3$ colloidal conjugates were assembled by 70-nm ZIF-8 NPs and BA$_2$ colloidal molecules (assembled by $S_{2090}V_{380}$ micelles and 50-nm silica NPs).

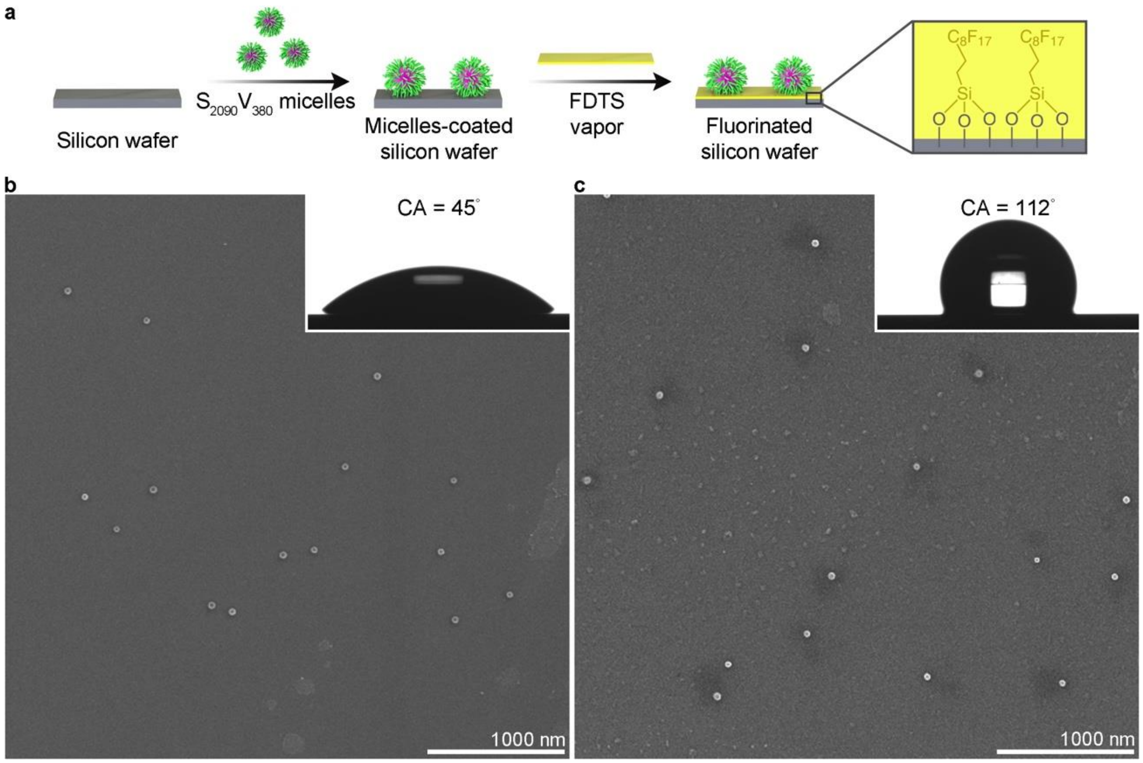

Supplementary Figure 49. Surface fluorination of $S_{2090}V_{380}$-micelles-coated silicon wafer. (**a**) Schematic illustration of the surface coating of $S_{2090}V_{380}$ micelles onto the silicon wafer surface and subsequent FDTS-based surface fluorination treatment. (**b** and **c**) SEM images of $S_{2090}V_{380}$-micelles-coated silicon wafer (**b**) before and (**c**) after surface fluorination treatment. After fluorination treatment, the $S_{2090}V_{380}$-micelles-coated silicon wafer surface transformed from hydrophilic to hydrophobic, as evidenced by the obvious increase in the CA value from 45° to 112° (inset photographs).

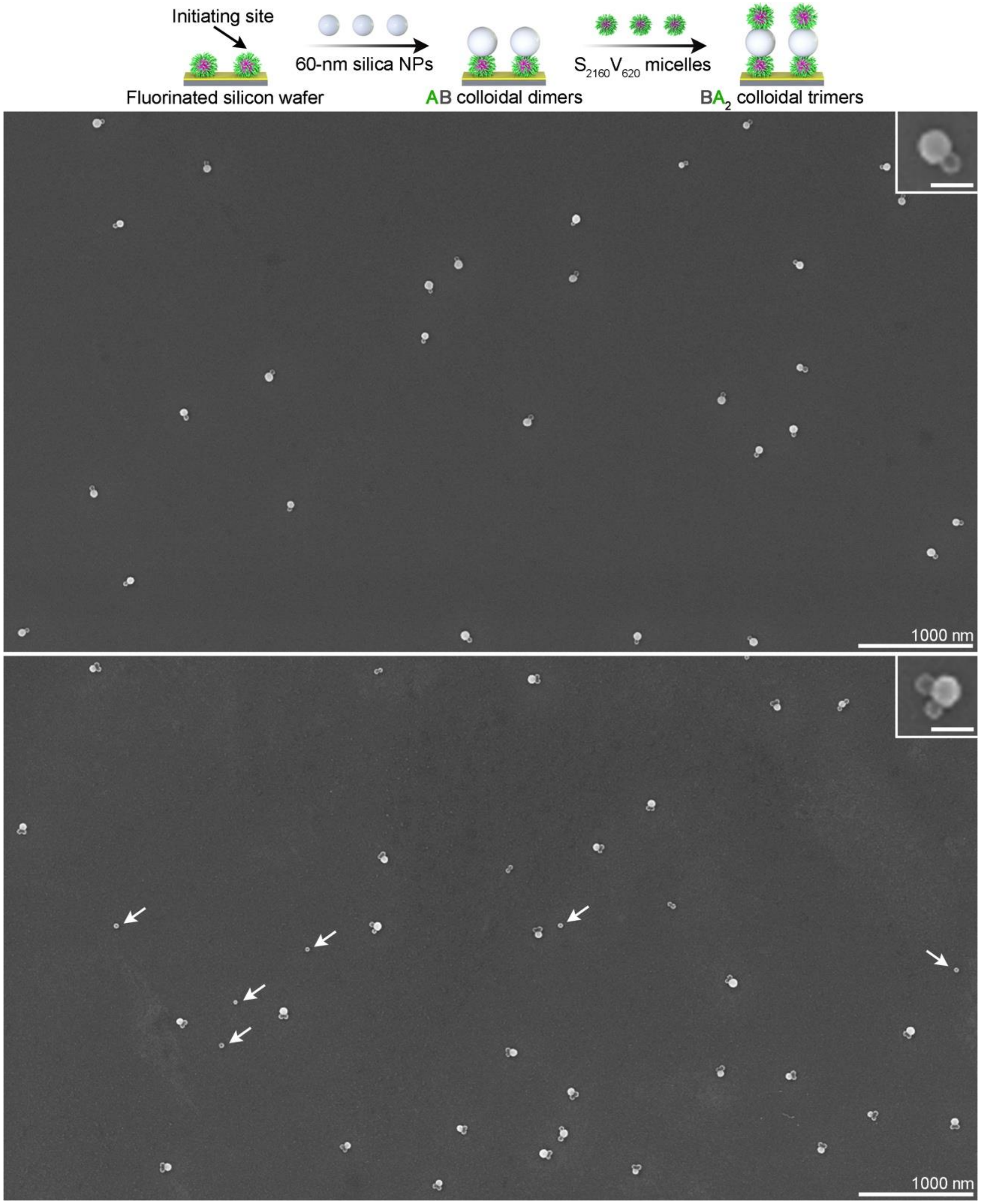

Supplementary Figure 50. Initiated growth of AB dimers and BA_2 trimers from silicon wafer surface. Immersion of the $S_{2090}V_{380}$-micelles-coated, fluorinated silicon wafer (Supplementary Fig. 49) in an ethanol solution of 60-nm silica NPs yields AB dimers (top). Further immersion in an ethanol solution of $S_{2160}V_{620}$ micelles results in the formation of BA_2 trimers (bottom). The white arrows denote a small amount of unassociated $S_{2160}V_{620}$ micelles that deposited on the silicon wafer surface, which is probably due to the alcoholysis of the Si-O-Si bonds and the leaching of FDTS from the surface. Inset scale bars = 100 nm.

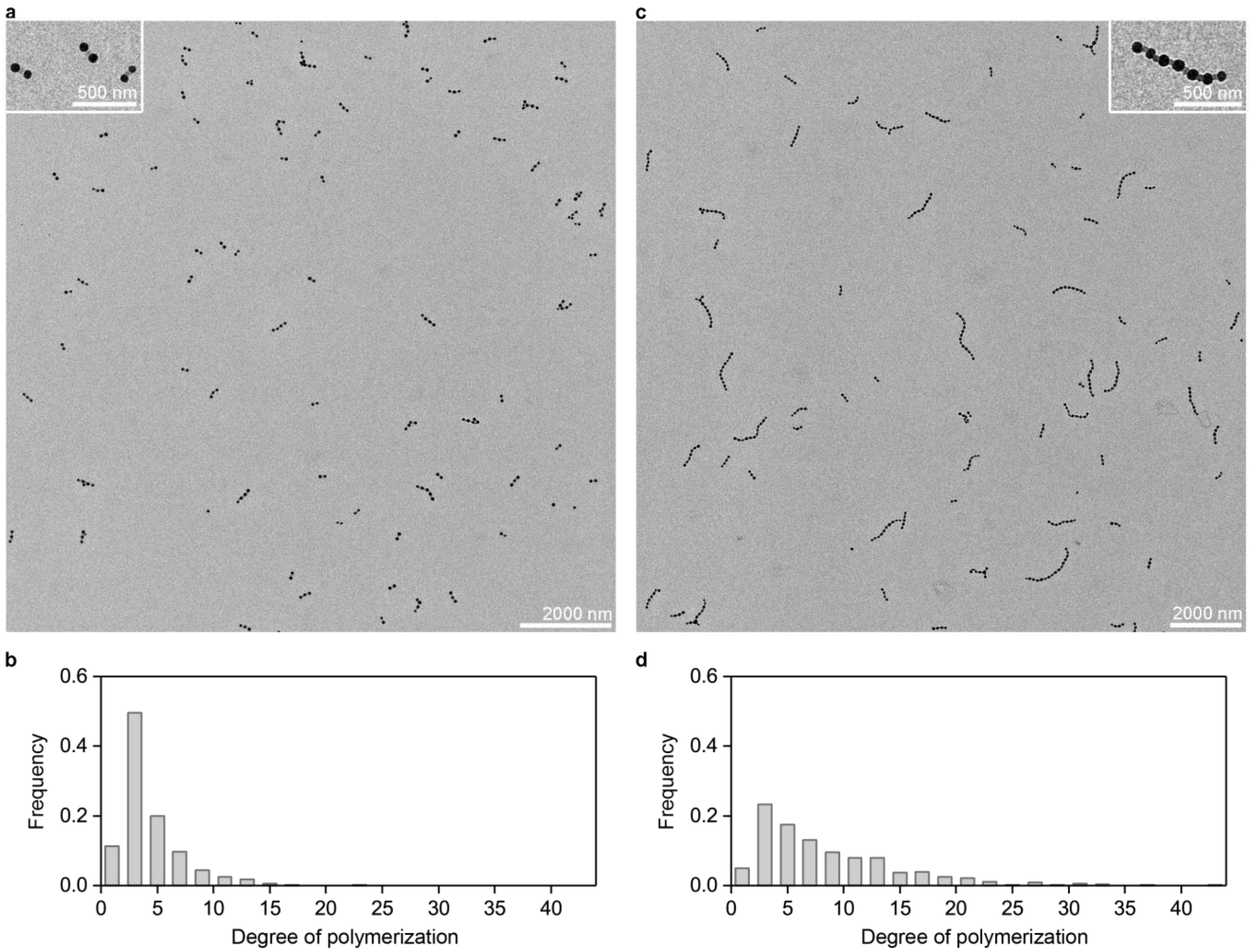

Supplementary Figure 51. Silica-NPs-capped colloidal chains with variable degree of polymerization assembled by $S_{2090}V_{380}$ micelles and 67-nm silica NPs. (**a** and **b**) Wide-field TEM image and statistical analysis of relatively short colloidal oligomers used for the fabrication of relatively short colloidal brushes. (**c** and **d**) Wide-field TEM image and statistical analysis of relatively long colloidal oligomers used for the fabrication of relatively long colloidal brushes.

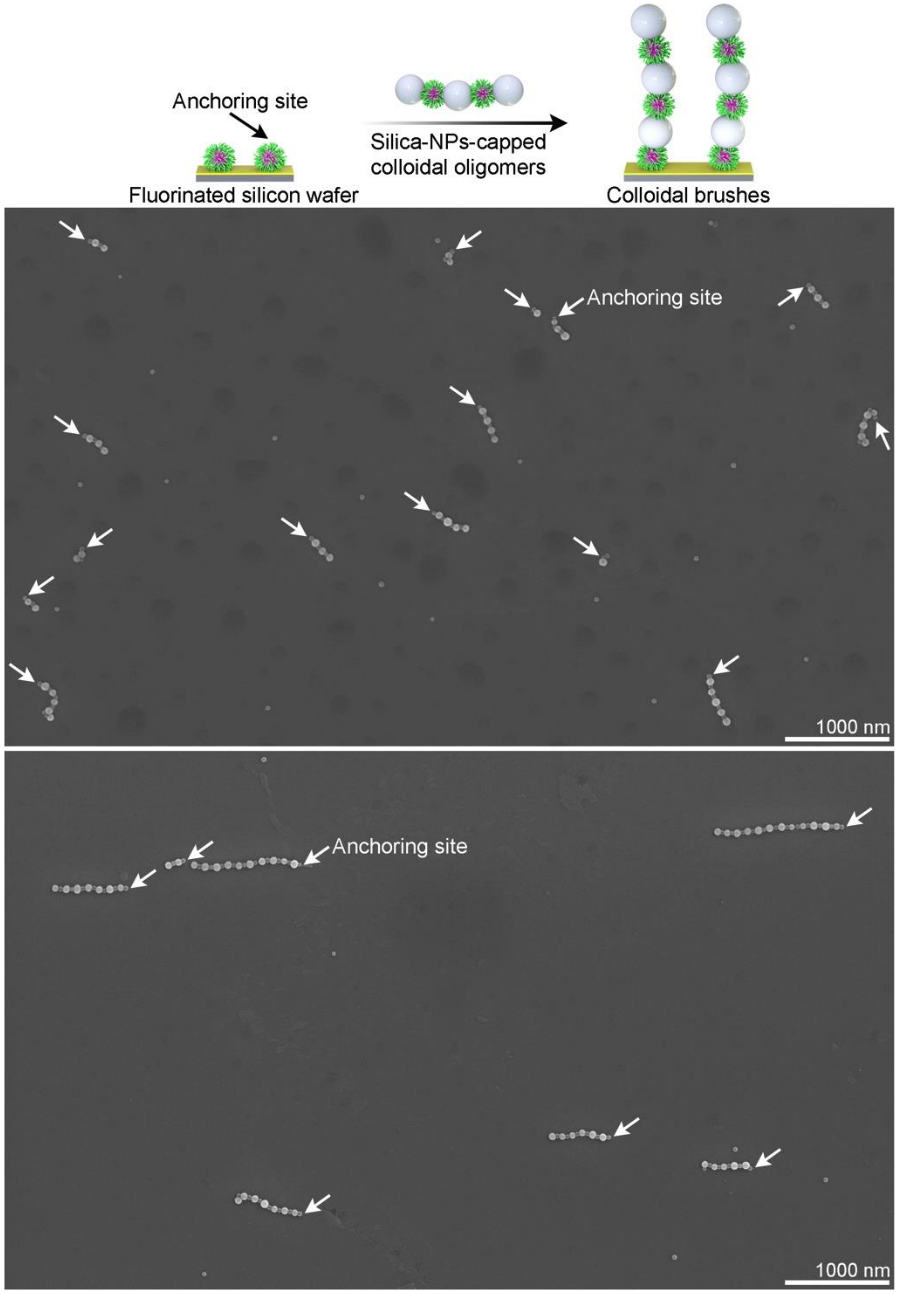

Supplementary Figure 52. Fabrication of colloidal brushes through a "grafting-to" approach. Relatively short (top) or long (bottom) colloidal brushes are grafted onto the silicon wafer surface by immersing the $S_{2090}V_{380}$-micelles-coated, fluorinated silicon wafer (Supplementary Fig. 49) into an ethanol solution of silica-NPs-capped short or long colloidal oligomers (formed by $S_{2090}V_{380}$ micelles and 67-nm silica NPs, Supplementary Fig. 51), respectively. The white arrows denote the micellar anchors preformatively immobilized on the silicon wafer surface.

Supplementary Methods

Preparation of silica NPs

Silica NPs with various diameters were synthesized by a modified Stöber method[1]. In a typical experiment, 1 mL of aqueous ammonia (28%) and 20 mL of ethanol were mixed thoroughly for 30 min, followed by the rapid injection of 0.06 mL of tetraethyl orthosilicate (TEOS) under vigorous stirring. After stirred for 30 min, the solution was allowed to age for 24 h at room temperature. Subsequently, the resulting solution was dialyzed against ethanol to remove the remaining ammonia before analysis.

Preparation of Au NPs

Au NPs with various diameters were synthesized through a seed-mediated growth method assisted with mild oxidation as previously reported[2]. To prepare the seed solution, 0.6 mL of an ice-cold, freshly prepared aqueous solution of $NaBH_4$ (0.01 M) was rapidly injected into a mixture containing 0.25 mL of an aqueous solution of $HAuCl_4$ (0.01 M) and 9.75 mL of an aqueous solution of cetyltrimethylammonium bromide (CTAB) (0.1 M) under vigorous stirring. After stirred for 2 min, the resultant mixture was mildly stirred for 3 h at room temperature. Then, 4 mL of an aqueous solution of $HAuCl_4$ (0.01 M), 9.75 mL of an aqueous solution of CTAB (0.1 M), and 190 mL of deionized water were mixed together, followed by the sequential addition of 15 mL of an aqueous solution of ascorbic acid (0.1 M) and 0.12 mL of the seed solution. The resultant mixture was gently shaken and left undisturbed overnight at room temperature. Subsequently, the as-synthesized small Au NPs were concentrated by 4 folds through centrifugation and redispersion in deionized water.

To prepare the targeted Au NPs, in a typical experiment, 4 mL of the above solution of small Au NPs was added into 30 mL of an aqueous solution of cetyltrimethylammonium chloride (CTAC, 0.025 M), followed by the sequential addition of 0.75 mL of an aqueous solution of ascorbic acid (0.1 M) and 1.5 mL of an aqueous solution of $HAuCl_4$ (0.01 M). The resultant mixture was kept under mild stirring for 3 h at 45 °C. After 3 h, the mixture was centrifuged and redispersed into 30 mL of an aqueous solution of CTAB (0.02 M). Then, 0.2 mL of an aqueous solution of $HAuCl_4$ (0.01 M) was added into the above solution and the resultant mixture was kept under mild stirring for 2 h at 45 °C. The obtained Au NPs were concentrated by 20 folds through centrifugation and redispersion in deionized water.

Preparation of core/shell structured silica-coated Au NPs

Silica-coating of Au NPs was carried out as previously reported with slight modifications[3]. In a typical experiment, 20 µL of an aqueous solution of NaOH (0.1 M) was added to 4 mL of the as-prepared 20-fold concentrated Au NPs solution, followed by the addition of 150 µL of a methanol solution of TEOS (20%, v/v). The resultant mixture was gently stirred for 1 h and left undisturbed for 24 h at room temperature. Subsequently, the mixture was washed with methanol by multiple centrifugation-redispersion cycles. The obtained silica-coated Au NPs were collected and redispersed in ethanol.

Preparation of PDA NPs

PDA NPs with various diameters were synthesized according to a previous literature[4]. In a typical experiment, 6 mL of ethanol, 14 mL of deionized water, and 0.3 mL of aqueous ammonia (28%) were mixed thoroughly for 30 min, followed by the rapid injection of 1.5 mL of an aqueous solution of dopamine hydrochloride (50 mg/mL) under vigorous stirring. After stirring for 24 h at room temperature, the resulting PDA NPs were collected by centrifugation and redispersion in ethanol.

Preparation of ZIF-8 NPs

ZIF-8 NPs were prepared as previously reported with slight modifications[5]. In a typical experiment, 5 mL of an aqueous solution of $Zn(CH_3COO)_2 \cdot 2H_2O$ (60 mg/mL) was added to 5 mL of an aqueous solution containing 2-methylimidazole (2.72 M) and CTAB (0.54 mM) with gentle stirring for a few seconds. After 15 s, the mixture was left undisturbed at room temperature for 2 h. Subsequently, the resulting ZIF-8 NPs were washed with deionized water and finally redispersed in deionized water.

Preparation of PS-*b*-P2VP block copolymers

All polymerizations were carried out in an inert atmosphere glovebox. All other manipulations were carried out under an open atmosphere unless otherwise stated. PS_{2090}-*b*-$P2VP_{380}$ (M_n = 257,600 g/mol, M_w/M_n = 1.15), PS_{2160}-*b*-$P2VP_{620}$ (M_n = 290,200 g/mol, M_w/M_n = 1.12), PS_{2040}-*b*-$P2VP_{750}$ (M_n = 291,300 g/mol, M_w/M_n = 1.14), PS_{2120}-*b*-$P2VP_{1230}$ (M_n = 350,100 g/mol, M_w/M_n = 1.14), PS_{2040}-*b*-$P2VP_{1590}$ (M_n = 379,600 g/mol, M_w/M_n = 1.17), PS_{2000}-*b*-$P2VP_{1910}$ (M_n = 409,100 g/mol, M_w/M_n = 1.22), PS_{2210}-*b*-$P2VP_{2510}$ (M_n = 494,100 g/mol, M_w/M_n = 1.23), PS_{1520}-*b*-$P2VP_{370}$ (M_n = 197,200 g/mol, M_w/M_n = 1.06), PS_{1220}-*b*-$P2VP_{440}$ (M_n = 173,300 g/mol, M_w/M_n = 1.12), PS_{1030}-*b*-$P2VP_{150}$ (M_n = 123,000 g/mol, M_w/M_n = 1.09), PS_{460}-*b*-$P2VP_{190}$ (M_n = 67,900 g/mol, M_w/M_n = 1.15) diblock copolymers were synthesized through sequential anionic polymerization in tetrahydrofuran (THF) in an inert argon atmosphere glovebox as reported in our previous work[6]. In a typical procedure, a solution of *n*-butyllithium (*n*-BuLi) in hexane (0.28 mL, 2.5 M) was added to 30 mL of dried THF under stirring and the mixture was allowed to age at room temperature overnight to remove the reactive impurities. Subsequently, 1.4 mL of styrene (12.18 mmol) was added to above solution at -78 °C, followed by the rapid injection of a solution of *sec*-butyllithium (*sec*-BuLi) in hexane (3.8 μL, 1.3 M) under vigorous stirring to initiate the polymerization of styrene. After 6 h, an aliquot (1/10) of the mixture was removed out and terminated by *p*-*tert*-butylphenol (PTBP). Subsequently, an excess amount (4.7 μL, 26.68 μmol) of diphenylethylene (DPE) was added into the mixture. A red color gradually developed within a few minutes. Then the solution was cooled to -78 °C and a solution of 2-vinylpyridine (0.14 mL, 1.3 mmol) and LiCl (1.88 mg, 44.46 μmol) in 1 mL of dried THF was added. After 1.5 h, PTBP was added to the resultant solution to terminate the living chain ends. Finally, the PS-*b*-P2VP diblock copolymer was isolated by precipitation into hexane and further into deionized water. The molecular weight of diblock copolymer was determined by combining the number-average molecular weight (M_n) of the first polystyrene block from GPC measurements with the block ratio obtained by integration of the corresponding ^{1}H NMR spectrum.

Preparation of PS-*b*-P2VP micelles

In a typical experiment, 100 mg of PS_{2090}-*b*-$P2VP_{380}$ ($S_{2090}V_{380}$) diblock copolymer solid was firstly dissolved in 10 mL of THF to yield a 10 mg/mL stock solution. Subsequently, to 0.9 mL of the above solution was added dropwise 33 mL of methanol via a syringe pump with an injection rate of 1.5 mL/h under mild stirring. The resulting solution was dialyzed against ethanol to remove THF, followed by filtration with a syringe filter (Titan, polytetrafluoroethylene membrane with a 0.22 μm pore size) to remove impurities. Finally, the solution was diluted to 30 mL by the addition of ethanol before use.

Characterization

Transmission electron microscopy (TEM) micrographs were obtained on a Thermo Scientific Talos L120C G2 microscope operated at 120 kV. High-resolution TEM micrographs, bright-field scanning transmission electron microscopy (BF-STEM) micrographs, high-angle annular dark-field STEM

(HAADF-STEM) micrographs and corresponding energy dispersive X-ray spectroscopy (EDS) elemental mapping micrographs were obtained on a Thermo Scientific Talos F200X microscope operated at 200 kV. Samples were prepared by placing one drop (ca. 10 μL) of solution on a carbon film-coated copper grid, which was placed on a piece of filter paper to remove excess solvent. No staining of the samples was necessary. Images were analyzed using the ImageJ software package (version 1.52a) developed at the US National Institute of Health. Scanning electron microscopy (SEM) micrographs were obtained on a TESCAN MAIA3 microscope operated at 5.0 kV. Samples were prepared by placing one drop (ca. 10 μL) of the solution on a clean silicon wafer. Before use, the silicon wafers were sonicated in acetone and deionized water for 30 min, respectively, and dried under a gentle stream of nitrogen. Then, the silicon wafers were subjected to oxygen-plasma treatment for 10 min. Before SEM characterization, the samples were sputter-coated with a thin layer of platinum for 15 s. Atomic force microscopy (AFM) height micrographs were obtained at ambient conditions using a Bruker Bio-FastScan atomic force microscope. Samples were prepared by placing one drop (ca. 10 μL) of the solution on a clean silicon wafer. Images were analyzed by NanoScope Analysis (version 1.80, an open source software program developed for AFM images). Gel permeation chromatography (GPC) measurements were performed on a Tosoh HLC-8320GPC gel permeation chromatograph. THF or DMF was used as the eluent at a flow rate of 1.0 mL/min. Sample was dissolved in the eluent overnight and filtered with a filter (Titan, polytetrafluoroethylene membrane with a 0.22 μm pore size) before analysis. Calibration was conducted using polystyrene standards. ^{1}H nuclear magnetic resonance (NMR) spectrum was recorded with a Bruker AVANCE III HD 500 NMR spectrometer. The samples were prepared by dissolving the polymers in $CDCl_3$. Static water contact angle (CA) measurements were conducted on a KRÜSS DSA100 Drop Shape Analyzer equipped with a high-speed camera at room temperature. 2 μL of water was dropped via the sessile drop method and the angle was measured by the circle fitting method. For each sample, at least five locations were tested in order to obtain the average CA value.

Supplementary References

1. Stöber, W., Fink, A. & Bohn, E. Controlled growth of monodisperse silica spheres in the micron size range. *J. Colloid Interface Sci.* **26**, 62–69 (1968).
2. Ruan, Q., Shao, L., Shu, Y., Wang, J. & Wu, H. Growth of monodisperse gold nanospheres with diameters from 20 nm to 220 nm and their core/satellite nanostructures. *Adv. Optical Mater.* **2**, 65–73 (2014).
3. Wu, W.-C. & Tracy, J. B. Large-scale silica overcoating of gold nanorods with tunable shell thicknesses. *Chem. Mater.* **27**, 2888–2894 (2015).
4. Liu, Y. et al. Dopamine-melanin colloidal nanospheres: an efficient near-infrared photothermal therapeutic agent for in vivo cancer therapy. *Adv. Mater.* **25**, 1353–1359 (2013).
5. Avci, C. et al. Self-assembly of polyhedral metal–organic framework particles into three-dimensional ordered superstructures. *Nat. Chem.* **10**, 78–84 (2018).
6. Zhu, H. et al. Blooming of block copolymer micelles into complex nanostructures on a surface. *Macromolecules* **52**, 3479–3485 (2019).
